# Distinguishing Short‐Term Versus Long‐Term Responses in Cover‐Class Structured Community Dynamics: A Test With Grassland Drought Response

**DOI:** 10.1111/ele.70182

**Published:** 2025-08-13

**Authors:** Aryaman Gupta, Samuel J. L. Gascoigne, György Barabás, Benjamin Wong Blonder, Man Qi, Erola Fenollosa, Rachael Thornley, Christina Hernandez, Andy Hector, Roberto Salguero‐Gómez

**Affiliations:** ^1^ Department of Biology University of Oxford Oxford UK; ^2^ Mathematical Institute University of Oxford Oxford UK; ^3^ School of Biological Sciences University of Aberdeen, Zoology Building Aberdeen UK; ^4^ Division of Biology, Department of Physics, Chemistry and Biology (IFM) Linköping University Linköping Sweden; ^5^ Institute of Evolution, Centre for Ecological Research Budapest Hungary; ^6^ Department of Environmental Science, Policy, and Management University of California Berkeley California USA

**Keywords:** disturbance, extreme precipitation, functional group, grasslands, integral projection model (IPM), interspecific interactions, pseudospectra, stress gradient hypothesis, transient instability

## Abstract

Climate change is increasing the magnitude and frequency of precipitation extremes. Consequently, grassland community dynamics are destabilising and becoming harder to predict since models typically simulate long‐term (asymptotic) behaviour, potentially neglecting short‐term (transient) behaviour. Here, we use cover data from an experiment performed over 8 years to model short‐ and long‐term responses of three functional groups (grasses, legumes, and non‐leguminous forbs) to precipitation extremes. We use Integral Projection Models (IPMs) and pseudospectral theory to track transient grassland community dynamics driven by response lags and interannual shifts. We show that the cover‐class structure and inter‐cover‐class interactions of functional groups make them transiently unstable but asymptotically stable, that is, disturbances are initially amplified before eventually dissipating. We also show that grasses dominate under irrigation, while legumes and forbs dominate under drought. We demonstrate that the pseudospectra of IPMs enable computationally and data‐wise inexpensive assessment of whether transient dynamics drive community responses to disturbances.

## Graphical abstract

We use Integral Projection Models (IPMs) and pseudospectral theory to track short‐term (transient) dynamics of a grassland subject to experimental precipitation shifts. We show that the cover‐class structure of functional groups makes them transiently unstable but asymptotically stable, that is, disturbances initially amplify before dissipating. The pseudospectra of community matrices from those IPMs enable computationally and data‐light assessment of transient community responses to disturbances.

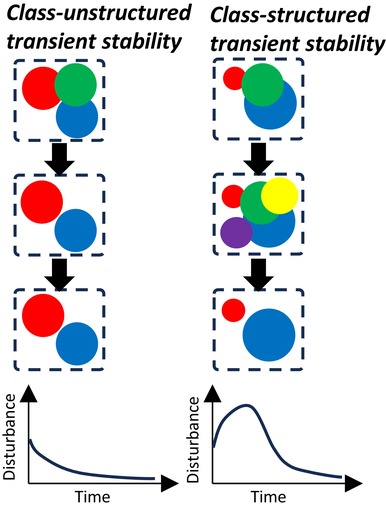

## 1 Introduction

Understanding how abiotic disturbances shape communities is a pressing concern for ecology (Sutherland et al. 2013; Hou and Wang 2023). Climate change is expected to increase the frequency and severity of disturbances such as precipitation shifts (Knapp et al. 2015), increasingly destabilising communities (Gaüzère et al. 2018). Hence, community models must capture these increasingly uncertain conditions for a range of data resolutions (Römer et al. 2024).

Community models commonly assume all individuals of interacting taxa are identical (de Roos 2020). Thus, these models often track performance via a single metric (e.g., number of individuals) and do not incorporate class‐structure, that is, how a trait like size or cover is distributed across individuals (but see Moll and Brown 2008; Fujiwara et al. 2011; Barabás et al. 2014; de Roos 2020; Johnson et al. 2024; Romero et al. 2025). However, population class‐structure can significantly shape communities, by e.g., determining a community's stability (Romero et al. 2025) or the niche overlap between species (Frank et al. 2011). Hence, we need models that explicitly incorporate class‐structure to capture its effect on community stability.

Integral Projection Models (IPMs; Easterling et al. 2000) are widely used to study structured population dynamics (Levin et al. 2022). Despite their potential to link interacting species' vital rates (e.g., survival, growth, reproduction) to population structures, IPMs have rarely been used in community ecology (Adler et al. 2010; Kayal et al. 2019; Peirce et al. 2023). This lack of adoption is partly because modelling interactions between structured populations is computationally demanding (Rossberg and Farnsworth 2011) and requires individual‐level data (Freckleton et al. 2011). Thus, to study population‐community interactions using IPMs, these data and computational costs must be addressed.

**FIGURE 1:**
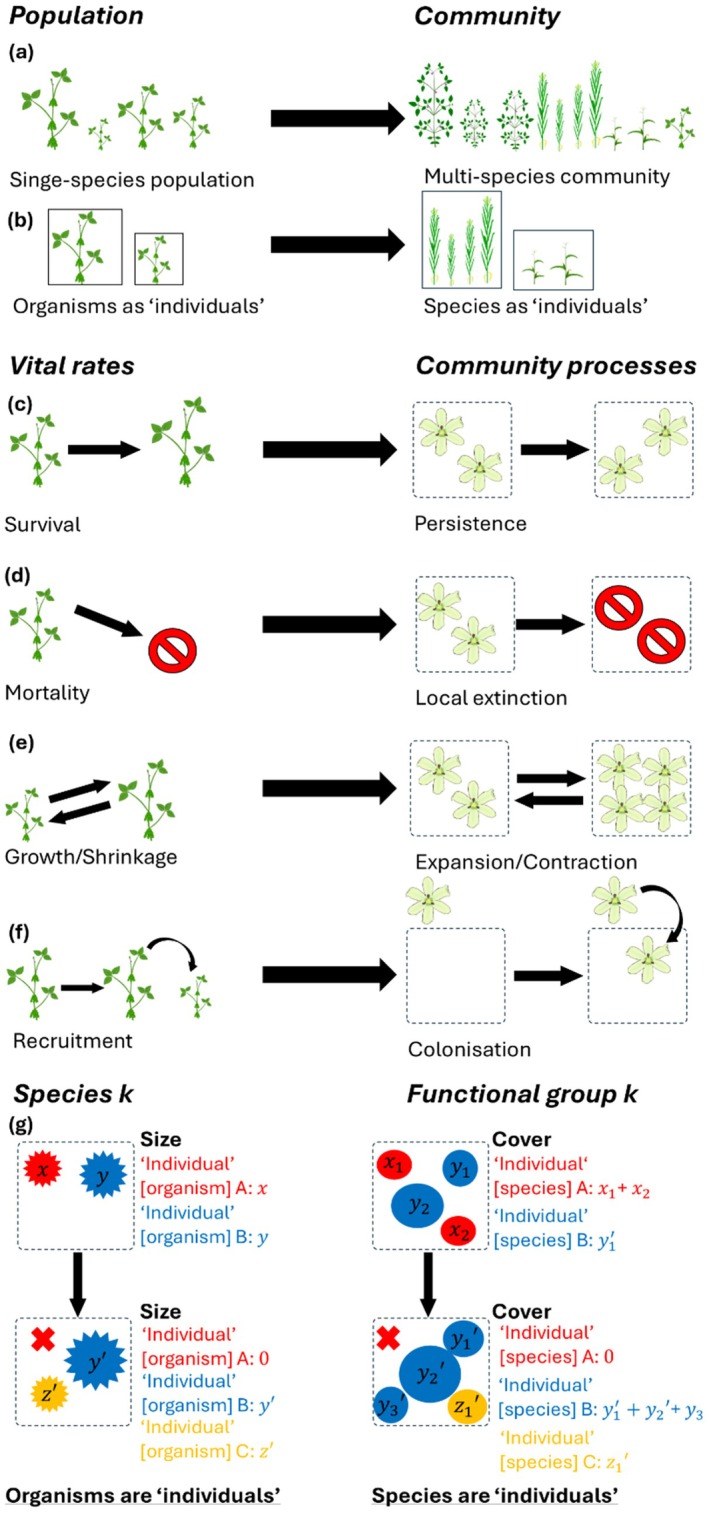
The shift in perspective from population vital rates to community processes. In this study, we deploy this shift to examine the impacts of experimentally manipulated precipitation on the cover‐class structure and transient dynamics of communities. The shift implies mapping how vital rates within a population draw analogous processes at the community level. This analogy is drawn by examining single versus multiple species populations (a, b), and their corresponding shifts in population to community processes (c–f). Further, instead of considering the performance of individual organisms in a species, we consider the performance of individual species in a functional group (g). (a) Instead of a single‐species population, here we consider a community as the population. (b) Instead of individual organisms, here we consider species as individuals. (c) Instead of survival of individuals, we consider persistence of species. (d) Instead of mortality of individuals, we consider local extinction of species. (e) Instead of growth and shrinkage of individuals, we consider the expansion and contraction of species' covers. (f) Instead of recruitment of new offspring, we consider colonisation of new species. (g) Instead of taking organisms as individuals to track how a species changes over time, we take species as individuals to track how a functional group changes over time. Under the (demographic) perspective of organisms as individuals, organism A experiences mortality (shifting from size *x* to size 0), organism B experiences growth (from size y to size *y*’), and organism C is recruited by the population (initial size *z*’). Under the perspective of species as individuals, species A experiences local extinction (shifting from cover *x*_1_ + *x*_2_ to cover 0), species B experiences growth (from cover *y*_1_ + *y*_2_ to cover *y*_1_’ + *y*_2_’ + *y*_3_’), and organism C colonises the plot (initial cover z_1_’). Flower icon by DBCLS https://togotv.dbcls.jp/en/pics.html is licensed under CC‐BY 4.0 Unported https://creativecommons.org/licenses/by/4.0/. All other icons by Daniel Carvalho https://figshare.com/authors/Plant_Illustrations/3773596 are licensed under CC‐BY 4.0 Unported https://creativecommons.org/licenses/by/4.0/.

Community dynamics can be modelled using IPMs by treating any *species* as an ‘*individual’*, whose ‘cover’ (as a community ecologist would quantify in the field) equals its ‘size’ (as a population ecologist would do in a population; Figure 1a,b; Ghiselin 1974). In other words, we treat any *community* as a ‘population of species’. Species experience community processes analogous to vital rates (Figure 1c–f). Thus, instead of representing individual organisms changing in size in a single‐species population (i.e., size‐class structure), we use IPMs to represent individual species changing in cover in a functional group (i.e., cover class‐structure, Figure 1g). To infer inter‐functional‐group interactions, we apply the standard theoretical coexistence approach of measuring how changes in the abundances of functional groups affect each other (Godoy 2019). This ‘species‐as‐individuals’ perspective has the downside of abstracting important intra‐functional‐group interactions (Rubio and Swenson 2024). However, it has the upside of capturing interactions between large numbers of species with a small number of functional‐group parameters (Chalmandrier et al. 2021) with comparable predictive performance to species‐level models (Tredennick et al. 2017). This ‘species‐as‐individual’ perspective is one of our study's main proposals that enables us to analyse structured community dynamics, by upscaling IPMs from populations to communities.

Communities are commonly assumed to exist near equilibrium (Morozov et al. 2024), a convenience that enables researchers to predict their long‐term (asymptotic) responses to perturbations (Van Meerbeek et al. 2021). Stability analyses of community equilibria are typically implemented by examining the eigenvalues of the community matrix ***M***, whose entries represent inter‐species interaction strengths (Kot 2001). However, sometimes more important than asymptotic outcomes are the short‐term (transient) behaviours after perturbation (Mcdonald et al. 2016), driven by for example, response lags, multiple timescales, or interannual abundance shifts (Hastings et al. 2018). If a community is transiently unstable, that is, if it may initially amplify a perturbation before decaying (Craine and Dybzinski 2013), repeated perturbations can drive a community far enough from its initial equilibrium to escape it (Morozov et al. 2024). For example, in the US, transient instabilities due to delayed responses to drought have caused a regime shift from asymptotically stable grasslands to shrublands (Bestelmeyer et al. 2018).

Pseudospectral theory can be used to analyse how perturbations from equilibrium develop transiently, before asymptotic dynamics take over (Trefethen and Embree 2005). For any disturbance of magnitude ϵ, the ϵ‐pseudospectrum of a matrix ***M*** is the region of the complex plane containing the union of eigenvalues of all structural perturbations of ***M*** by size ϵ, that is, all matrices ***M* + *E*** with the matrix norm (i.e., size) of ***E*** smaller than ϵ (Trefethen and Embree 2005). The pseudospectra of ***M*** enable us to calculate a metric that indicates how much a community may transiently amplify a disturbance (see Methods). Here, for the first time, pseudospectral theory is used with IPMs to examine how cover‐class structure shapes transient community stability.

Because grasslands frequently experience major disturbances such as precipitation shifts, they regularly undergo transient dynamics out of equilibrium. These dynamics could be explained either by the community moving among alternative fixed stable states, or by the community tracking equilibria that are constantly moving (Lohier et al. 2016; Hastings et al. 2018). Hence, observational data do not offer direct insights into equilibria or transient dynamics (Dudney et al. 2017; Kleinhesselink 2020). Nevertheless, long‐term empirical studies on grassland communities suggest that they typically fluctuate close enough to equilibrium that matrix models broadly capture their dynamics (Wilson and Roxburgh 1992; Yandi et al. 2023).

Thus, to test if our framework accurately captures our study system, we generate directional hypotheses. From the stress gradient hypothesis (Bertness and Callaway 1994), interspecific interactions should be competitive under increased precipitation and facilitative under drought (Lima et al. 2022). Hence, under increased precipitation, an equilibrium should be attained where grasses are more abundant than legumes and non‐leguminous forbs (subsequently ‘forbs’), as grasses typically overshadow forbs and legumes (Hautier et al. 2009). The greater abundance of grasses in this case should stabilise the community due to their ability to quickly grow and recover post‐disturbance when they are not water‐limited (Li et al. 2024). Under drought, an equilibrium should be attained where legumes and forbs are more abundant than grasses due to their drought‐tolerance (Hallett et al. 2017; Liu et al. 2024), where this drought‐tolerance stabilises the community. If our model produces results consistent with these a priori predictions, that would serve as internal validation in our model's novel predictions regarding how stage‐structure shapes transient dynamics, which are: (i) These equilibria should be transiently unstable due to recovery lags and interannual abundance shifts (Dudney et al. 2017; Fischer et al. 2020); (ii) Cover‐class structure should shape transient instability as species across cover‐classes vary by competitiveness (Hallett et al. 2023).

**FIGURE 2:**
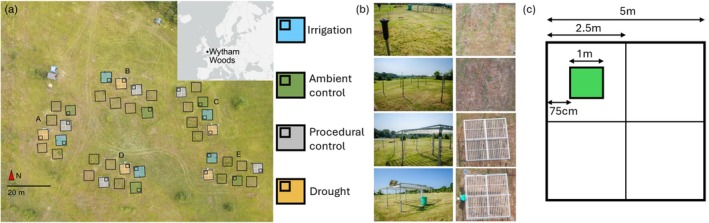
Schematic of the DroughtNet experimental treatments implemented at RainDrop, Wytham Woods (adapted from Jackson et al. 2024). (a) Distribution of DroughtNet treatments across the site, spatially arranged into five blocks (A–E). The treatments are: Irrigation (+50% precipitation; blue), ambient control (green), procedural controls (grey), and drought (−50% precipitation; orange). Each square represents the 5 × 5 m permanent plot, and each smaller square within indicates the randomly chosen position of a quadrat from which community data were collected every June from 2016 to 2023. (b) Photographs of the treatment structures for each treatment, from a ground level (left) and aerial (right) view. (c) Design of each 5 × 5 m permanent plot, which is subdivided into four quarters (each 2.5 × 2.5 m). Percent‐cover (abundance) readings for each species were taken using a 1 × 1 m quadrat placed at the centre of the quarter chosen for observation.

To assess the short‐ and long‐term class‐structured stability of grasslands in response to precipitation shifts with coarse cover data, we analyse pseudospectra of class‐structured community matrices generated using functional‐group IPMs. The IPMs are parametrised with data from a grassland located at Wytham Woods (UK; Figure 2a). There, communities with grasses, legumes and forbs were exposed to irrigation, control, and drought treatments (Figure 2). We test these hypotheses, to be inferred from our model: (H1.1) Interspecific interactions will be more competitive under irrigation than control; (H1.2) Interactions will be more facilitative under drought than control; (H2.1) Under irrigation, grasses will be more abundant than legumes and forbs; (H2.2) Under drought, legumes and forbs will be more abundant than grasses; (H3) Communities will be transiently unstable; (H4) Cover‐class structure will shape transient community stability.

## 2 Materials and Methods

### 2.1 Experimental Setup

We conducted our study at the RainDrop (Rainfall and Drought platform) ecological experiment site (https://www.ecologicalcontinuitytrust.org/raindrop). The study site is a calcareous chalkland grassland (Gibson and Brown 1991) located at the Upper field site at Wytham Woods, in Oxford, UK (51°46′7.57″ N, 1°19′49.58″ W, 84–167 m a.s.l.; Figure 2a). The experiment consists of five randomised blocks (A–E) of four 5 × 5 m treatment plots (*n* = 20 plots): irrigation (+50% ambient precipitation, via sprinklers), an ambient control (no treatment and no roof structure), procedural control (a roof structure with inverted gullies which let all ambient precipitation through), and drought (−50% ambient precipitation, via a roof structure with gullies; Figure 2a,b). Within each plot, we monitored communities within a 1 × 1 m quadrat placed in the middle of one of the plot's four quarters (determined randomly), to avoid edge effects (Figure 2c; following Smith et al. 2024).

The procedural control was established to evaluate whether the shelter structure has any effect beyond the intended precipitation reduction. However, analysis of data from our site shows that procedural control plots have the same community composition and precipitation responses as ambient control plots (Jackson et al. 2024). Thus, we merge data from procedural and ambient controls and refer to them as‘control’.

### 2.2 Data Collection

Biotic data for three functional groups (grasses, legumes, and forbs) were collected in the summer (June–July) for 8 years (2016–2023) to parametrise our models of cover‐class structured community stability. The data collected at each plot included species identification (including functional group: legume, grass, or forb) and species percent‐cover from 0% to 100% (a proxy for abundance). Each year, for each plot and block, all species in the 1 × 1 m quadrat were identified during the growing season peak (mid‐to‐late June). Species names were recorded following the International Plant Name Index (IPNI 2024). Percent‐cover (referred to subsequently as ‘abundance’) data were assessed as the average of estimates given by three observers to the nearest ±0.5%. Due to the three‐dimensional structure of the community, whereby covers of different species can overlap, the sum of abundance values of all species in a quadrat can exceed 100%. After collecting these data, we summed the abundance values of all species within each functional group separately. We denote the overall abundance of each functional group *k* by *N*_*k*_.

### 2.3 Model Construction

To test whether interspecific interactions were competitive under irrigation (H1.1) and facilitative under drought (H1.2), we constructed models of our community analogues to vital rates for each of the 3 functional groups × 3 treatments. Using version 1.1–35.5 of the *lme4* package (Bates et al. 2015) in version 4.2.2 of R (R core team 2022), we constructed generalised linear models of persistence (continued existence of a species in a plot), expansion (increased cover), and colonisation (new species entering the plot) for each functional group × treatment condition. These models are analogous, respectively, to survival, growth/shrinkage, and reproduction vital rate models for a single‐species demographic model (Merow et al. 2014, Figure 1). Colonisation was treated as independent from the abundance of species within the quadrat, analogous to the treatment of reproduction in a demographic model. We tested the effects of inter‐ and intra‐functional‐group interactions on functional‐group persistence, expansion, and colonisation by including overall abundances of grasses (*N*_*g*_), legumes (*N*_*l*_) and forbs (*N*_*f*_) as fixed explanatory variables. Before analysis, individual abundance, *N*_*g*_, and *N*_*f*_ values were log transformed to ensure linear and quadratic models could fit the data. Since *N*_*l*_ was zero in two instances, *e* ^−1^ ≈*0.37%* was added to each *N*_*l*_ value before log‐transformation. To construct models of persistence, expansion and colonisation, regressions were performed on every possible pair (*t*, *t* + *1*) of abundance data. The random effects of years or blocks were not considered, as they were not included in our IPMs. Under a given treatment, persistence *s*_*k*_ *(x)* was modelled as a continuous variable between 0 and 1 using a logit link function, where *(x)* is (logged) cover in year *t* for functional group *k*, by the base model
(1)

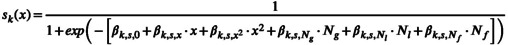

Expansion *g*_*k*_ (*x*, *y*) was modelled using a Gaussian distribution *G* (μ_*g,k*_ (*x*), σ_*g,k*_(*x*)), where *y* is the (logged) cover in year *t* + *1*, by the base model

(2) $$g_{k}\left(x,y\right)=G\left(y\;|\;\mu_{g,k}\left(x\right),\sigma_{g,k}\left(x\right)\right)=\frac{1}{\sigma_{g,k}\left(x\right)\sqrt{2\pi }}\exp\left[\frac{-{\left(y-\mu_{g,k}\left(x\right)\right)}^{2}}{2\sigma_{g,k}{\left(x\right)}^{2}}\right]$$

where the mean *μ*_*g*,*k*_ (*x*) and standard deviation *σ*_*g,k*_ (*x*) were given by base models

(3) $$\begin{array}{c} \mu_{g,k}\left(x\right)=\beta_{k,\mu_{g},0}+\beta_{k,\mu_{g},x}\cdot x+\beta_{k,\mu_{g},x^{2}}\cdot x^{2}+\beta_{k,\mu_{g},N_{g}}\cdot N_{g}\\ \;+\beta_{k,\mu_{g},N_{l}}\cdot N_{l}+\beta_{k,\mu_{g},N_{f}}\cdot N_{f} \end{array}$$

(4) $$\begin{array}{c} \sigma_{g,k}\left(x\right)=\beta_{k,\sigma_{g},0}+\beta_{k,\sigma_{g},x}\cdot x+\beta_{k,\sigma_{g},x^{2}}\cdot x^{2}+\beta_{k,\sigma_{g},N_{g}}\cdot N_{g}\\ \;+\beta_{k,\sigma_{g},N_{l}}\cdot N_{l}+\beta_{k,\sigma_{g},N_{f}}\cdot N_{f} \end{array}$$

Colonisation *f*_*k*_(*y*) was assumed to be lognormally distributed, and was thus modelled using a Gaussian distribution *G* (*μ*_*f*_, *σ*_*f*_) by base models

(5) $$f_{k}\left(y\right)=G\left(\mu_{f,k},\sigma_{f,k}\right)=\frac{1}{\sigma_{f,k}\sqrt{2\pi }}\exp\left[\frac{-{\left(y-\mu_{f,k}\right)}^{2}}{2{\sigma_{f,k}}^{2}}\right]$$

where the mean *μ*_*f,k*_ and standard deviation *σ*_*f*,*k*_ were given by base models

(6) $$\mu_{f,k}=\beta_{k,\sigma_{g},0}+\beta_{k,\sigma_{g},N_{g}}\cdot N_{g}+\beta_{k,\sigma_{g},N_{l}}\cdot N_{l}+\beta_{k,\sigma_{g},N_{f}}\cdot N_{f}$$

(7) $$\sigma_{f,k}=\beta_{k,\sigma_{g},0}+\beta_{k,\sigma_{g},N_{g}}\cdot N_{g}+\beta_{k,\sigma_{g},N_{l}}\cdot N_{l}+\beta_{k,\sigma_{g},N_{f}}\cdot N_{f}$$

To test our hypotheses that interactions would be competitive under irrigation (H1.1) and facilitative under drought (H1.2), we compared a set of candidate models, constructed using the MuMIn package (Bartoń 2024). Equations ((1), (2), (3), (4), (5), (6), (7)) describe our base model for each candidate model set. The base model included (only for persistence/expansion) linear and squared abundance of individual species of the functional group, and (for colonisation too) overall abundances of grasses, legumes, and forbs. Our use of individual species abundances to construct a functional group model is analogous to using individual organism sizes to construct a single‐species demographic model (Figure 1a,b,g). The candidate model set consisted of the base model and all possible models derived by excluding all possible combinations of the variables of interest from the base model. For each functional group × treatment, this procedure generated 2^5^ = 32 candidate models of persistence and expansion, and 2^3^ = 8 models of colonisation. From our candidate model set, models with lowest AICc (within two units) were identified. Then, the model with lowest AICc that was biologically plausible (e.g., without infinite growth) were chosen (see Tables S1–S45). Table S46 shows our final selected models. For excluded terms, the *β* coefficient was set to 0 in the above equations. To find support for H1.1, coefficients of *N*_*k*_ terms would need to be negative in functional‐group models under irrigation. To find support for H1.2, coefficients of *N*_*k*_ terms would need to be positive in functional‐group models under drought.

To test our hypotheses about how our treatments would influence community dynamics (H2.1 and H2.2), we used our models selected by lowest AICc value to construct IPMs (following Easterling et al. 2000) for each functional group × treatment. Our IPMs link the cover‐class distribution of individual species in a functional group at time t to the cover‐class distribution of individual species at time *t* + *1*. For a functional group *k*, the IPM describing the relationship between the number *n*_*k*_ (*x*, *t*) of ‘individuals’ (species here) of cover‐value *x* at time *t* and the number *n*_*k*_ (*y*, *t* + *1*) of individuals of cover‐value *y* at time *t* + *1*, is

(8) $$n_{k}\left(y,t+1\right)=\int_{L}^{U}\left[s_{k}\left(x\right)g_{k}\left(x,y\right)+f_{k}\left(y\right)\right]n_{k}\left(x,t\right)\mathrm{dx}=\int_{L}^{U}K_{k}\left(x,y\right)n_{k}\left(x,t\right)\mathrm{dx}$$

Here, *L* = *log* 1 = 0 and *U* = *log* (100) ≈4.605 are the lower and upper bounds of the observed abundance for an individual species. These bounds are sufficiently small and large, respectively, to avoid eviction. The first term *s*_*k*_ *(x)·g*_*k*_ *(x, y)* quantifies changes in abundance (Figure 1e) conditional on persistence (Figure 1c). The second term *f*_*k*_ *(y)* quantifies colonisation (Figure 1f). The resulting *kernel surface* ***K***_*k*_ (*x, y*) = *s*_*k*_(*x*)·*g*_*k*_ (*x, y*) *+ f*_*k*_(*y*) represents all possible transitions for an individual species from abundance × at time *t* to abundance *y* at time *t* + *1*.

To simulate how the influence of each functional group on the other groups changes over time (H2.1 and H2.2), all three functional‐group IPMs were discretised and run for 300 annual‐length time‐steps to find equilibrium values of *N*_*g*_, *N*_*l*_, and *N*_*f*_ for each treatment (see Supporting Information). To find support for H2.1, the equilibrium value of *N*_*g*_ under irrigation would need to be greater than the equilibrium values of *N*_*l*_ and *N*_*f*_. Conversely, to find support for H2.2, the equilibrium values of *N*_*l*_ and *N*_*f*_ under drought would need to be greater than the equilibrium value of *N*_*g*_.

### 2.4 Pseudospectral Analysis

After completing the IPM simulation runs, we tested our hypotheses regarding whether our study system exhibits transient instability (H3), and on whether cover‐class structure influences transient stability (H4). To test these hypotheses, for each treatment we performed pseudospectral analysis on class‐structured community matrix models constructed using our final IPMs (at *t* = 300) for each functional group. To contextualise this analysis, we briefly explain pseudospectral theory below.

**FIGURE 3:**
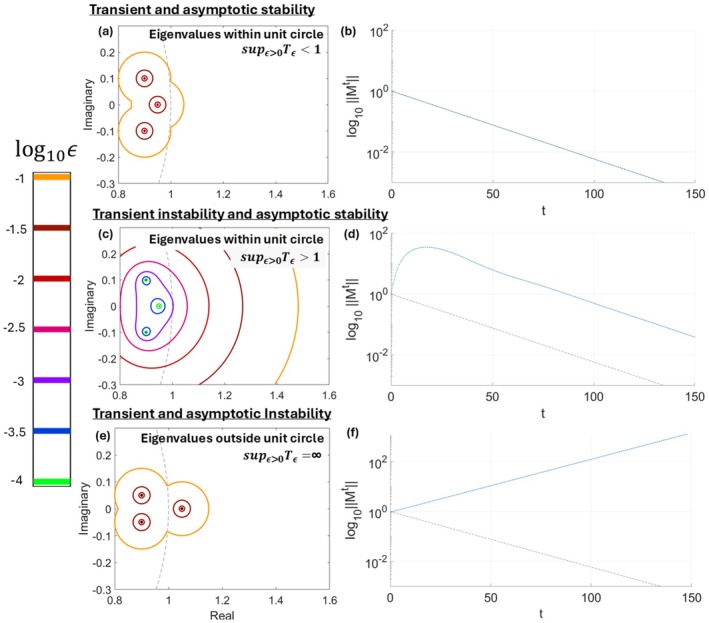
Examples of matrices and their pseudospectra that do or do not exhibit transient instability. In (a), the dots represent the eigenvalues of a community matrix ***M***, and the coloured lines are the boundaries of their ϵ‐pseudospectrum, where the colours on the scale to the left represent the ϵ value for each pseudospectrum. Here, each value ϵ represents the degree of noise that the matrix ***M*** of interaction coefficients between cover‐classes and functional groups is subject to due to structural perturbations. The grey dashed line is the boundary of the unit circle, where $x^{2}+y^{2}=1$, which represents the threshold between dynamical perturbation expansion and contraction. Here, $T_{\epsilon }=\frac{{⍴}_{\epsilon }\left(M\right)-1}{\epsilon }$, where ρ_*ϵ*_(***M***) is the largest of the distances between any point of the ϵ‐pseudospectrum and the origin of the complex plane. Then, sup_ϵ>0_ *T*_*ϵ*_, is the greatest value *T*_*ϵ*_ takes across all possible ϵ > 0. (a) The pseudospectrum for a matrix such that sup_ϵ>0_ *T*_*ϵ*_ < 1. The community corresponding to this matrix is transiently and asymptotically stable. (b) A plot of how dynamical perturbations may change for a transiently and asymptotically stable matrix such as the one in panel a. The y – axis represents the magnitude $\left\|M^{t}\right\|$ (on the log_10_ scale), and the *x*‐axis represents time t following an initial perturbation. Following the blue line, any perturbations (here, shifts in composition) immediately decay. (c) The pseudospectrum of a matrix such that sup_ϵ>0_ *T*_*ϵ*_ > 1 and such that all eigenvalues all lie within the unit circle. Hence, the community corresponding to this matrix is transiently unstable but asymptotically stable. (d) A plot of how perturbations may change for a transiently unstable and asymptotically stable matrix such as the one in panel c. Following the blue line, perturbations in this case may initially amplify before eventually starting to decay once *t* ≈20. (e) The pseudospectrum of a matrix whose eigenvalues lay outside the unit circle. The community corresponding to this matrix is transiently and asymptotically unstable, i.e., perturbations grow forever. (f) A plot for how a perturbation changes for an asymptotically unstable matrix such as the one in panel e. Following the blue line, any perturbation in this case is amplified exponentially and never decays.

Pseudospectral theory was first used in ecology by Barabás and Allesina (2015). This theory analyses how perturbations from equilibrium develop transiently, before asymptotic dynamics take over (Trefethen and Embree 2005). Let ***M*** be a square matrix, such as an IPM after discretisation (Easterling et al. 2000). For any small ϵ, the ϵ‐pseudospectrum of ***M*** is the subset of the complex plane containing the union of the eigenvalues of all structural perturbations of ***M*** by size ϵ, that is, all matrices ***M* + *E*** with the matrix norm of ***E*** smaller than ϵ (Trefethen and Embree 2005). Crucially, pseudospectra enable us to calculate a lower bound sup_ϵ>0_ *T*_*ϵ*_ on a community's potential degree of transient instability after a dynamical perturbation, i.e., a perturbation to an equilibrium of **M**, before it attains its asymptotic behaviour (Supporting Information). Here

(9) $$\begin{array}{c} T_{\epsilon }=\frac{{⍴}_{\epsilon }\left(M\right)-1}{\epsilon }, \end{array}$$

where ρ_*ϵ*_(***M***) is the largest of the distances between any point of the ϵ‐pseudospectrum and the origin of the complex plane. Then, sup_ϵ>0_ *T*_*ϵ*_, is the greatest value *T*_*ϵ*_ takes across all possible ϵ> 0. To interpret sup_ϵ>0_ *T*_*ϵ*_, we illustrate three mock‐examples of pseudospectra in Figure 3. If sup_ϵ>0_ *T*_*ϵ*_ < 1 (Figure 3a), the community does not exhibit transient instability, i.e., any perturbations (here, shifts in composition) immediately decay (Figure 3b). If sup_ϵ>0_ *T*_*ϵ*_ > 1 and no eigenvalue crosses beyond the unit circle (Figure 3c), the community exhibits transient instability (Figure 3d), i.e., perturbations may be initially amplified before decaying. Finally, if an eigenvalue crosses the unit circle (Figure 3e), the community exhibits transient and asymptotic instability (Figure 3f), i.e., any perturbations grow indefinitely.

Finally, to determine whether our communities exhibit transient instability (H3) and whether cover class‐structure influences community stability (H4), we constructed a cover‐class structured community matrix for each treatment using the functional‐group IPMs for that treatment (Supporting Information). The cover‐class structured community matrix for each treatment represents interaction strengths between individuals (species) of any functional group and cover‐class with individuals of any other functional group and cover‐class. We then calculated the pseudospectra for these community matrices using the *Eigtools* package (Wright 2002). To find support for H3 and H4, the pseudospectra of the community matrix for each treatment would need to have sup_*ϵ > 0*_ *T*_*ϵ*_ > 1, and the matrix eigenvalues would have to lay within the unit circle. H3 would then be supported since sup_*ϵ > 0*_ *T*_*ϵ*_ > 1 would indicate transient instability, while the eigenvalues laying within the unit circle would indicate asymptotic stability. H4 would then be supported since different perturbations to the initial cover‐class structure would produce different transient stability outcomes. For example, perturbations produced by linear combinations of eigenvectors would be transiently and asymptotically stable. Yet, since sup_*ϵ > 0*_ *T*_*ϵ*_ > 1, other perturbations would be transiently unstable (Trefethen and Embree 2005).

## 3 Results

### 3.1 Inter‐Functional Group Interaction Shifts

Inter‐functional‐group interaction coefficients were exclusively negative (and thus competitive) under irrigation (Table 1): increases in the abundance of a functional group suppress the abundance of another, thus supporting H1.1. Under drought, several positive interaction coefficients were recorded, interspersed with negative coefficients (Table 1). Hence, some facilitative interactions emerged, with species pairs positively impacting each other. This partially supports H1.2. Specifically, forbs facilitate the persistence and expansion of legumes and colonisation by other forbs, while grasses facilitate the expansion and colonisation of forbs, and colonisation of legumes (Table 1).

**TABLE 1:** Selected interaction coefficients for persistence, expansion and colonisation of each treatment group × functional group.

Community vital rate	Treatment	Irrigation			Control			Drought		
	Func. group, interaction term	Grasses	Legumes	Forbs	Grasses	Legumes	Forbs	Grasses	Legumes	Forbs
Persistence	Log (*N*_g_)				−0.611		*−0.481*	*−0.56*	***−1.703***	−0.147
	Log (*N*_l_ + e^−1^)	***−1.01***	***−1.491***	**−0.274**	***−0.678***	***−0.008***	−0.038	*−0.22*		
	Log (*N*_f_)	−0.299	−0.475	***−0.678***	**−0.418**		***−0.591***		0.497	***−0.728***
Expansion, mean	Log (*N*_g_)	*−0.347*			***−0.425***		***0.236***	−0.13		**0.163**
	Log (*N*_l_ + e^−1^)		−0.193	−0.038		−0.059	**−0.057**	−0.19		***−0.075***
	Log (*N*_f_)								0.187	
Colonisation, mean	Log (*N*_g_)				0.386		**0.25**		0.38	0.13
	Log (*N*_l_ + e^−1^)						−0.011		*−0.146*	
	Log (*N*_f_)			**0.179**			***0.171***			***0.341***

*Note:* This table demonstrates a shift in inter‐functional group interactions from competitive to facilitative as precipitation decreases. Cell colours vary from orange (for negative values) to cyan (for positive values) for overall abundance terms *N*_f_ to indicate, for a given community vital rate, whether the overall abundance of *k* has a facilitative or competitive effect. The increasing presence of cyan cells further to the right of the table indicates that interactions shift from being primarily competitive to facilitative as water availability decreases (from irrigation to control to drought). Coefficients with *p* < 0.001 are in bold and italics; coefficients with *p* < 0.01 are in bold, and those with *p* < 0.1 are in italics. Table S46 is an extended version of this table which also includes all other parameters.

### 3.2 Predicted Relative Abundances of Functional Groups

**FIGURE 4:**
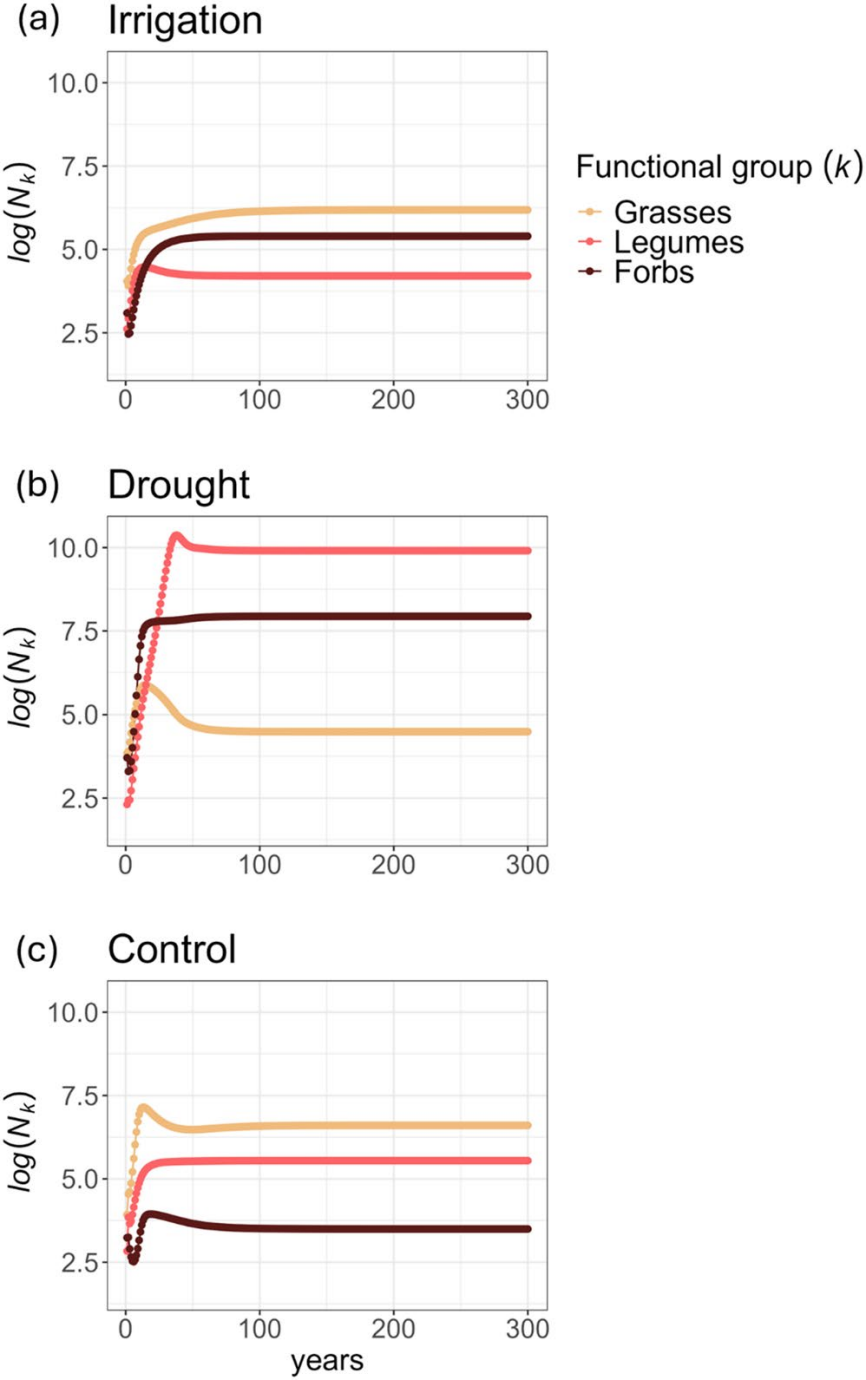
Multi‐functional group IPM predictions of functional‐group abundance for each treatment, for 300 years. All functional groups reach a steady abundance value (i.e., log (*N*_*k*_) changes by < 10^−6^) by the last 10 years. (a) Under the irrigation treatment, grasses dominate over (i.e., have a greater overall abundance than) forbs, which in turn dominate over legumes. (b) Under the control treatment, grasses dominate over legumes, which in turn dominate over forbs. (c) Under the drought treatment, Legumes dominate over forbs, which in turn dominate over grasses.

Our functional‐group IPM simulations supported our hypotheses about how precipitation shifts would alter communities (H2.1, H2.2). Under irrigation, grasses would be dominant, with an overall abundance roughly an order of magnitude larger than forbs and legumes (Figure 4a), thus supporting H2.1. Under drought, after 7 years, forbs would dominate over grasses, and after 15 years, legumes would also dominate over grasses (Figure 4c), thus supporting H2.2. However, although our IPMs predicted the relative levels of abundance between functional groups in line with prior work on grasslands, they consistently predicted much higher abundances than empirically observed. The overestimation can be observed in Figure 4, since the overall abundance values of all functional group × treatments are higher at *t* = 300 than at *t* = 0. Abundance values at *t* = 0 represent the observed average for each functional group × treatment.

### 3.3 Transient Instability Driven by Cover Class‐Structure

**FIGURE 5:**
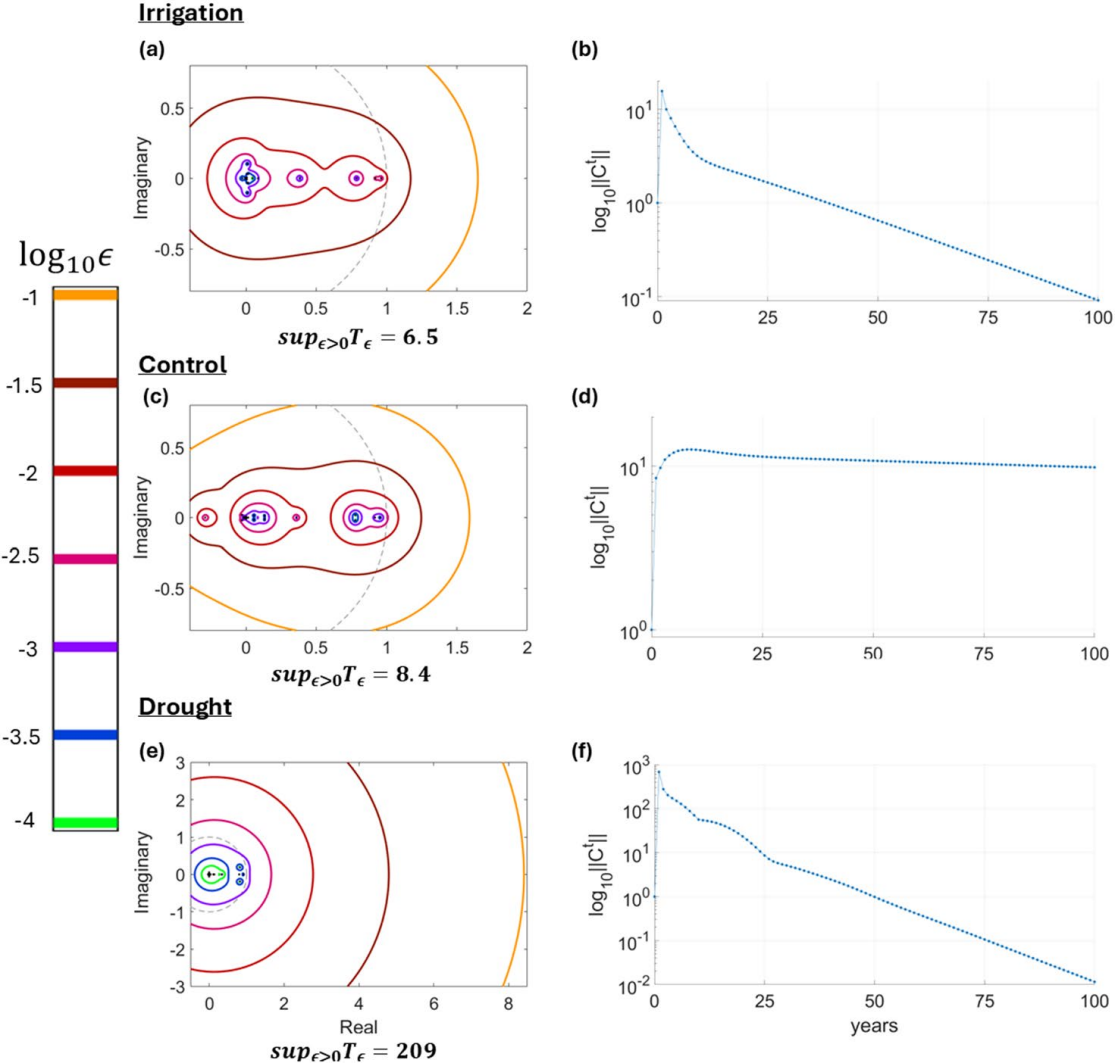
Pseudospectra and eigenvalues of the class‐structured community matrix produced using the functional‐group IPMs at the end of the simulation run for each treatment. Panels (a, c), and (e) are the pseudospectra, respectively, for our class‐structured community matrices of the irrigation, control, and drought treatments. Panels (b, d), and (f) are plots of how, respectively, a transiently unstable perturbation may change for the class‐structured community matrix C of the irrigation, control, and drought treatments. The y axis of these plots represents the magnitude $\left\|C^{t}\right\|$ on the log_10_ scale. For every treatment overall (a, c, e), no eigenvalues lie outside the unit circle. Therefore, they are all asymptotically stable—i.e., any perturbation is eventually suppressed as t→∞, as indicated by asymptotic decay in plots in b, d, and f. However, the pseudospectra are all such that sup_ϵ>0_ *T*_*ϵ*_ > 1 (a, c, e). This implies that every treatment is transiently unstable—i.e., they can all transiently amplify small perturbations, before perturbations are eventually suppressed, as indicated by the transient amplification seen in plots b, d, and f. To see the values of *T*_*ϵ*_ plotted against *ϵ* for each treatment, see Figure S1.

Our pseudospectral analysis supported H3 by predicting that all treatments would be transiently unstable before attaining long‐term stability as indicated by their eigenvalues (Figure 5a–f). Transient instability was predicted across all treatments because sup_ϵ > 0_ *T*_*ϵ*_ > 1 for every community matrix (Figure 5a,c,e). Then, asymptotic stability was predicted across all treatments because the eigenvalues of every community matrix lay within the unit circle (Figure 5a,c,e). Furthermore, our analysis supported H4 by indicating that, for all treatments, transient stability depends on cover‐class structure. If a perturbation from the initial cover‐class distribution consists solely of e.g., a linear combination of eigenvectors, that perturbation would be transiently stable. Conversely, since sup_ϵ > 0_ *T*_*ϵ*_ > 1, it follows that some perturbations would be transiently unstable.

## 4 Discussion

Using 8 years of abundance data on the effects of precipitation shifts on grasslands, we examined how cover‐class structure shapes transient community stability using a novel, relatively low‐data method. By examining data from our field site, we found that interactions between functional groups are almost exclusively competitive under irrigation, while some interactions are facilitative under drought. Using these data, we parametrised interlinked functional‐group Integral Projection Models (IPMs; Easterling et al. 2000). Our functional‐group IPM simulations correctly predicted that grasses dominate over legumes and forbs under irrigation, and that legumes and forbs dominate over grasses under drought. Most importantly, by analysing the pseudospectra of the community matrices generated from these IPMs, we gained novel insights into the transient dynamics of grassland communities. We found that all treatments were transiently unstable, potentially amplifying perturbations initially before eventually suppressing them, as predicted by asymptotic eigenvalue analysis (Kot 2001). Furthermore, we found that the cover‐class structure of communities can shape their transient stability.

Put together, our findings suggest that grassland community interactions and equilibria dynamically respond to shifting precipitation (Lohier et al. 2016; Bektaş 2024). Our models predict that grasslands experience significant shifts in functional‐group composition and cover‐class structure as precipitation levels change, driven by shifts in interactions between functional group cover‐classes (Li et al. 2024; Liu et al. 2024). Our models also showed that interactions between functional group cover‐classes, as represented by our class‐structured community matrices, drive transient instability across precipitation levels (Dudney et al. 2017; Hallett et al. 2023). This transient instability, coupled with asymptotic stability, potentially explains how grassland communities shift their compositions and interactions in response to changing precipitation while maintaining stability (Fukami et al. 2005; Fischer et al. 2020).

Our approach includes two key advances. First, we demonstrated how IPMs can be scaled up from populations to communities. We do this by employing a' species‐as‐individual’ perspective to overcome the excessive computational and data‐collection costs of demographic analyses, which typically require extensive data on individual‐level trajectories of survival, development, and reproduction (Salguero‐Gomez and Gamelon 2021). This scaling‐up offers a comparatively data‐ and parameter‐light tool to mechanistically understand how community analogues to population vital rates drive emergent patterns such as disturbance‐driven succession or range shifts (García‐Cervigón et al. 2021; Peirce et al. 2023). Furthermore, this scaling‐up could be applied to phenomena for which demographic‐level IPMs already exist, such as forest species colonisation (Zhao et al. 2021; Drees et al. 2023) and reef responses to extreme weather (Kayal et al. 2019; Cresswell et al. 2024). Second, the application of pseudospectral theory to analyse the transient stability of IPM‐derived class‐structured community models is new, as previously they have only been deployed for unstructured community matrix models (Barabás et al. 2014; Kostić et al. 2016; Caravelli and Staniczenko 2015). As the usage of IPMs continues to grow (Levin et al. 2022), their pseudospectra can demonstrate how interactions between e.g., size or cover‐classes shape the transient stability of populations and communities. Indeed, using IPMs with pseudospectra could enable conservationists to assess class‐structure driven transient instability in vulnerable communities as an early‐warning signal (Oro and Martínez‐Abraín 2023).

Pseudospectra offer a valuable complement to eigenvalues for assessing community properties, by informing us about transient stability in addition to their asymptotic stability. Revealing how communities respond in the short‐ and mid‐term to disturbances uncovers potentially crucial dynamics that cannot be observed in asymptotic, eigenvalue‐based predictions. If a community is exposed to a perturbation and is shown via its pseudospectra to exhibit transient instability, further perturbations could drive the community away from its initial equilibrium to a new one (Caravelli and Staniczenko 2015). A promising avenue of future work would be to analyse which perturbation regimes shift transiently unstable systems far enough from asymptotically stable equilibria to escape them altogether.

Pseudospectra offer several benefits in relation to alternative transient instability metrics. Since pseudospectra can be found for any matrix (Trefethen and Embree 2005), they are easily applied to existing models. The applicability of pseudospectra to existing matrix models means they are far less data‐intensive than e.g., demographic models (Tredennick et al. 2017) or models which reconstruct the geometry of entire dynamical state‐spaces (Sánchez‐Pinillos et al. 2024). As large datasets now exist for IPMs (Levin et al. 2022) and MPMs (Salguero‐Gómez et al. 2015; Salguero‐Gómez et al. 2016), pseudospectra offer an exciting opportunity for geographically and taxonomically wide‐ranging macroecological assessment of how transient instability shapes ecosystems. Further, while standard matrix stability metrics measure outcomes right after a perturbation is applied (reactivity) and a long time after (asymptotic stability), pseudospectra give us insight into what happens in between (Trefethen and Embree 2005). Finally, pseudospectra of structured population models, like IPMs (Easterling et al. 2000) and matrix population models (Caswell 2001) can capture the role that interactions between cover classes play in generating transient instability (de Roos 2020). By contrast, multivariate analyses of factors shaping populations use uncoupled linear models for each population stage without inter‐stage interactions (Adler and HilleRisLambers 2008). This model structure produces uniform stability predictions over the short‐ and long‐term and thus cannot distinguish short‐term instability from long‐term stability (Trefethen and Embree 2005).

Our findings are pertinent to recent theoretical work indicating that size‐class structures of populations can significantly influence the stability of their communities (de Roos 2020). These works demonstrate that structured populations support more stable interaction webs than unstructured ones. We showed by contrast that for our system, coarse cover‐class structure at the functional‐group level drives a limited degree of transient instability. Ecologically, this cover‐class structure driven instability is likely driven by the differences in competitive ability by cover‐class (Hallett et al. 2023). Even in grasslands where functional‐group level composition is stable, shifts often occur between cover‐class structure equilibria, e.g., from equilibria with many small‐cover species to equilibria with a few large‐cover species (Fukami et al. 2005).

We found support for the stress gradient hypothesis (Bertness and Callaway 1994). Under irrigation, functional groups tend to suppress each other. Under drought, some functional groups facilitate each other, although competitive interactions persist. These results suggest our stress gradient was relatively weak. Consequently, interaction shifts were possibly limited by weak stress amelioration and niche differences (Qi et al. 2018). This aligns with the fact that our study system is very drought tolerant, as calcareous grasslands typically are (Fry et al. 2018).

Despite these advances, our model consistently over‐predicted abundance across functional group × treatments. There are at least five possible explanations: (1) our study site is presently recovering from the effects of long‐term grazing, (2) our model does not account for inhibitory abiotic effects, (3) our model does not account for higher‐order interactions, (4) our model abstracts important intra‐functional‐group interactions, (5) our model assumes that our communities are near‐equilibrium assumptions, despite grasslands being frequently out of equilibrium. For (1), Jackson et al. (2024) showed that at our study site, there was a treatment‐independent increase in species richness with time. Therefore, models which best fit our data may implicitly reflect how our grassland communities would develop if their post‐recovery secondary succession never ceases. For (2), potential abiotic inhibitors include limitations of space or nutrients, whose physical limitation, prior to any additional limitation due to inter‐functional group interactions, may induce density‐dependence and thus constrain functional‐group growth (Craine and Dybzinski 2013). Without these inhibitors, our simulated functional groups possibly grew larger than their real‐life counterparts before inter‐functional group interactions constrained them (Senthilnathan 2023). For (3), the inclusion of higher‐order interactions, whereby the interaction of two species is mediated by a third species, produces more realistic coexistence predictions than models with only linear, additive interaction terms (Mayfield and Stouffer 2017). For (4), Rubio and Swenson (2024) argue that abstracting interactions to a functional‐group level can cause the interactions of dominant species within the group to overshadow less dominant ones. Thus, this abstraction may lead to overprediction of the functional group's expansion capacity. For (5), few studies have explicitly evaluated how grassland dynamics conform to near‐equilibrium assumptions (Wilson and Roxburgh 1992). Yet, little work has been done to precisely determine the range of conditions where near‐equilibrium assumptions hold (Roxburgh and Wilson 2000). Hence, future studies can build upon our framework by (i) implementing it for a range of ecosystems at differing levels of succession, (ii) including abiotic constraint terms, (iii) including higher‐order interactions into community analogues of vital rate models, (iv) incorporating parameters that capture intra‐functional group variance, and (v) examining the range of conditions where near‐equilibrium assumptions hold true for grasslands.

Increasingly, it is recognised that instability is the norm, not the exception, in ecological communities (Capdevila et al. 2021; Morozov et al. 2024). Hence, our results provide clear motivation to examine transient instability further, since it can significantly shape community outcomes by transiently driving them between equilibria or out of equilibrium (Hastings et al. 2018; Morozov et al. 2024). As climate change drives instability in ecosystems worldwide (Pecl et al. 2017), there is a growing call for treating ecosystems as'dynamic regimes’ rather than'equilibrium regimes’ (Coulson 2021, Sánchez‐Pinillos et al. 2023). However, in addressing this call, theory and empirical work have occurred separately with little interaction (Dakos and Kéfi 2022; Oro and Martínez‐Abraín 2023). Our study bridges this separation by examining models parametrised by field data (rather than simulated communities). Future studies should continue bridging this divide. In an increasingly changing Earth system, the tools to assess ecological communities must reflect this instability for a range of data resolutions (Römer et al. 2024) to secure our mechanistic understanding and conservation practice (Sánchez‐Pinillos et al. 2024).

## Author Contributions

Initial conceptualisation was by Roberto Salguero‐Gómez with input from György Barabás and Aryaman Gupta. The experimental site was established by Andy Hector. Data were collected by Aryaman Gupta, Roberto Salguero‐Gómez, Andy Hector, Erola Fenollosa, and Man Qi, among others (listed in acknowledgements). Aryaman Gupta wrote the code for the models for this paper with input from Roberto Salguero‐Gómez and Samuel J.L. Gascoigne. Roberto Salguero‐Gómez, Benjamin Wong Blonder, Rachael Thornley, Christina Hernandez, Samuel J.L. Gascoigne, Erola Fenollosa, and Man Qi assisted in developing the concepts behind our models in greater detail. Aryaman Gupta and Roberto Salguero‐Gómez wrote the first draft. György Barabás aided in developing and refining the mathematical underpinnings of our modelling framework. All co‐authors then assisted in refining and editing the draft before submission.

## Peer Review

The peer review history for this article is available at https://www.webofscience.com/api/gateway/wos/peer‐review/10.1111/ele.70182.

## Supporting information


Data S1.

## Data Availability

The data and code used for this study have been uploaded on Zenodo (DOI: 10.5281/zenodo.13896326) and will be restricted so that only peer‐reviewers can access it until the review process is complete. Upon the publication of this manuscript in a journal, the data will immediately be made freely available.
