# Supplementary material for: Distinguishing Short‐Term Versus Long‐Term Responses in Cover‐Class Structured Community Dynamics: A Test With Grassland Drought Response
Source: Ecol Lett. 2025 Aug 13;28(8):e70182. doi: 10.1111/ele.70182 (PMC12344748; doi:10.1111/ele.70182)

**Supplementary Online Materials**

Variation in precipitation drives differences in interactions and transient short-term instability between grassland functional groups: a stage-structured community approach

**Contents**

[**Details of the study site** 3](#_Toc187336096)

[**Experimental setup** 3](#_Toc187336097)

[**Model selection** 4](#_Toc187336098)

[**Table S1 – Model selection for grasses under irrigation, persistence** 4](#_Toc187336099)

[**Table S2 – Model selection for grasses under irrigation, expansion, mean** 5](#_Toc187336100)

[**Table S3 – Model selection for grasses under irrigation, expansion, s.d.** 6](#_Toc187336101)

[**Table S4 – Model selection for grasses under irrigation, colonisation, mean** 7](#_Toc187336102)

[**Table S5 – Model selection for grasses under irrigation, colonisation, s.d.** 7](#_Toc187336103)

[**Table S6 – Model selection for legumes under irrigation, persistence** 7](#_Toc187336104)

[**Table S7 – Model selection for legumes under irrigation, expansion, mean** 8](#_Toc187336105)

[**Table S8 – Model selection for legumes under irrigation, expansion, s.d.** 9](#_Toc187336106)

[**Table S9 – Model selection for legumes under irrigation, colonisation, mean** 10](#_Toc187336107)

[**Table S10 – Model selection for legumes under irrigation, colonisation, s.d.** 10](#_Toc187336108)

[**Table S11 – Model selection for forbs under irrigation, persistence** 10](#_Toc187336109)

[**Table S12 – Model selection for forbs under irrigation, expansion, mean** 11](#_Toc187336110)

[**Table S13 – Model selection for forbs under irrigation, expansion, s.d.** 12](#_Toc187336111)

[**Table S14 – Model selection for forbs under irrigation, colonisation, mean** 13](#_Toc187336112)

[**Table S15 – Model selection for forbs under irrigation, colonisation, s.d.** 13](#_Toc187336113)

[**Table S16 – Model selection for grasses under control, persistence** 13](#_Toc187336114)

[**Table S17 – Model selection for grasses under control, expansion, mean** 14](#_Toc187336115)

[**Table S18 – Model selection for grasses under control, expansion, s.d.** 15](#_Toc187336116)

[**Table S19 – Model selection for grasses under control, colonisation, mean** 16](#_Toc187336117)

[**Table S20 – Model selection for grasses under control, colonisation, s.d.** 16](#_Toc187336118)

[**Table S21 – Model selection for legumes under control, persistence** 16](#_Toc187336119)

[**Table S22 – Model selection for legumes under control, expansion, mean** 18](#_Toc187336120)

[**Table S23 – Model selection for legumes under control, expansion, s.d.** 19](#_Toc187336121)

[**Table S24 – Model selection for legumes under control, colonisation, mean** 19](#_Toc187336122)

[**Table S25 – Model selection for legumes under control, colonisation, s.d.** 20](#_Toc187336123)

[**Table S26 – Model selection for forbs under control, persistence** 20](#_Toc187336124)

[**Table S27 – Model selection for forbs under control, expansion, mean** 21](#_Toc187336125)

[**Table S28 – Model selection for forbs under control, expansion, s.d.** 22](#_Toc187336126)

[**Table S29 – Model selection for forbs under control, colonisation, mean** 22](#_Toc187336127)

[**Table S30 – Model selection for forbs under control, colonisation, s.d.** 23](#_Toc187336128)

[**Table S31 – Model selection for grasses under drought, persistence** 23](#_Toc187336129)

[**Table S32 – Model selection for grasses under drought, expansion, mean** 24](#_Toc187336130)

[**Table S33 – Model selection for grasses under drought, expansion, s.d.** 25](#_Toc187336131)

[**Table S34 – Model selection for grasses under drought, colonisation, mean** 26](#_Toc187336132)

[**Table S35 – Model selection for grasses under drought, colonisation, s.d.** 26](#_Toc187336133)

[**Table S36 – Model selection for legumes under drought, persistence** 26](#_Toc187336134)

[**Table S37 – Model selection for legumes under drought, expansion, mean** 27](#_Toc187336135)

[**Table S38 – Model selection for legumes under drought, expansion, s.d.** 28](#_Toc187336136)

[**Table S39 – Model selection for legumes under drought, colonisation, mean** 29](#_Toc187336137)

[**Table S40 – Model selection for legumes under drought, colonisation, s.d.** 29](#_Toc187336138)

[**Table S41 – Model selection for forbs under drought, persistence** 29](#_Toc187336139)

[**Table S42 – Model selection for forbs under drought, expansion, mean** 30](#_Toc187336140)

[**Table S43 – Model selection for forbs under drought, expansion, s.d.** 32](#_Toc187336141)

[**Table S44 – Model selection for forbs under drought, colonisation, mean** 33](#_Toc187336142)

[**Table S45 – Model selection for forbs under drought, colonisation, s.d.** 33](#_Toc187336143)

[**Table S46. Selected models for persistence, expansion and colonisation of each treatment group** $\boldsymbol{\times}$ **functional group.** 33](#_Toc187336144)

[**Details of model implementation to find overall abundance equilibrium** 35](#_Toc187336145)

[**Mathematical details of our pseudospectral analysis** 36](#_Toc187336146)

[**Construction of the overall community matrices for each treatment** 37](#_Toc187336147)

[**References** 41](#_Toc187336148)

# **Details of the study site**

Our site is a calcareous chalk grassland, with alkaline soil of shallow depth (300mm-500mm). According to the Köppen-Geiger climate classification, the site has a temperate maritime climate (Cfb) (Peele et al., 2007). During the years when our data were collected, 2016-2023, the mean annual temperature on the site was 11.5 ℃, with a daily temperature range between -5 ℃ and 26 ℃. The annual total precipitation was 686 mm, with a daily precipitation range of between 0-40 mm (Fenollosa et al. 2024). The study site is mowed twice a year, first mid-growing season (late July), and then at the end of the growing season (late September), as per European grassland management regulations (Török et al., 2018). Further details of our study site are found in Fenollosa et al. (2024) and Jackson et al. (2024).

# **Experimental setup**

In the drought plots, a rainwater shelter syphons off 50% of the naturally occurring precipitation via gulleys into storage containers by the side of the plot (Fig. 2b). The water in these containers is then sprayed onto the irrigation plots using sprinklers that are connected to the containers by pipes and a pump system (Fig. 2b). This design ensures that the changes in precipitation for the experimental plots are proportional to background levels of precipitation at the site. The ambient control had no structure and no experimental treatment (Fig. 2b). For the procedural control, shelters with inverted gullies are used (which let 100% precipitation fall through; Fig. 2b) to evaluate whether the shelter structure has any effect on the plot beyond the intended reduction in precipitation. Potential confounding effects included changes in boundary layer dynamics that could influence vegetation via changes in temperature, humidity or light availability.

# **Model selection**

Tables S1 – S45 provide the AICc values for each possible combination of log-transformed linear or quadratic relationships of percentage-cover, and overall cover of grasses (*N_g_*), legumes (*N_l_*), and forbs (*N_f_*) to include in our survival, persistence and expansion models for each functional group × treatment. To select from these models, the models with the least AICc value were first chosen, and IPMs were run using them. If they produced biologically unrealistic results (*e.g.* unbounded growth, or standard deviation eventually becoming negative), models with the next lowest AICc value was chosen, and this process of iteration was repeated until a suite of models was found which produced realistic results. For each table, the model that was selected through this process is indicated in bold.

## **Table S1 – Model selection for grasses under irrigation, persistence**

| (Intercept) | I((log(size))^2) | log(Nf) | log(Ng) | log(Nl + exp(-1)) | log(size) | df | logLik | AICc | delta | weight |
| --- | --- | --- | --- | --- | --- | --- | --- | --- | --- | --- |
| 3.124855 | -0.14649 | NA | NA | -1.01695 | 0.872829 | 4 | -147.73 | 303.6108 | 0 | 0.208568 |
| **4.007948** | **-0.15646** | **-0.29884** | **NA** | **-1.01033** | **0.94093** | **5** | **-146.787** | **303.8001** | **0.189364** | **0.189726** |
| 1.870752 | -0.17499 | -0.39524 | 0.584034 | -0.99731 | 0.976156 | 6 | -145.995 | 304.3071 | 0.696363 | 0.147243 |
| 3.155395 | NA | NA | NA | -0.99321 | 0.450329 | 3 | -149.277 | 304.6437 | 1.032923 | 0.124438 |
| 4.055239 | NA | -0.29447 | NA | -0.99102 | 0.485162 | 4 | -148.344 | 304.8382 | 1.227449 | 0.112904 |
| 1.712361 | -0.1554 | NA | 0.337602 | -1.00776 | 0.883073 | 5 | -147.443 | 305.1115 | 1.500691 | 0.098487 |
| 2.493525 | NA | -0.36573 | 0.426015 | -0.97733 | 0.473792 | 5 | -147.9 | 306.0248 | 2.414031 | 0.06238 |
| 2.316088 | NA | NA | 0.199338 | -0.98548 | 0.442224 | 4 | -149.171 | 306.492 | 2.881272 | 0.049384 |
| 3.312265 | 0.089516 | NA | NA | -0.94953 | NA | 3 | -153.191 | 312.4723 | 8.861516 | 0.002483 |
| 4.020297 | 0.097706 | -0.23127 | NA | -0.94522 | NA | 4 | -152.602 | 313.353 | 9.74226 | 0.001599 |
| 2.352452 | 0.085087 | NA | 0.229015 | -0.94159 | NA | 4 | -153.049 | 314.2473 | 10.63654 | 0.001022 |
| 2.50822 | 0.091349 | -0.29875 | 0.412296 | -0.93176 | NA | 5 | -152.177 | 314.5786 | 10.96782 | 0.000866 |
| 3.546044 | NA | NA | NA | -0.94043 | NA | 2 | -156.148 | 316.3413 | 12.73053 | 0.000359 |
| 1.714954 | NA | NA | 0.433822 | -0.92911 | NA | 3 | -155.58 | 317.2493 | 13.63852 | 0.000228 |
| 3.968818 | NA | -0.13565 | NA | -0.93612 | NA | 3 | -155.93 | 317.9491 | 14.33837 | 0.000161 |
| 1.798781 | NA | -0.24937 | 0.600743 | -0.91992 | NA | 4 | -154.956 | 318.0623 | 14.45148 | 0.000152 |
| 1.243436 | -0.13395 | -0.37221 | NA | NA | 0.847412 | 4 | -163.005 | 334.1588 | 30.54807 | 4.85E-08 |
| 1.285848 | NA | -0.34814 | NA | NA | 0.445832 | 3 | -164.109 | 334.3084 | 30.69759 | 4.50E-08 |
| -0.232 | -0.14593 | -0.44311 | 0.414617 | NA | 0.867751 | 5 | -162.402 | 335.0293 | 31.41849 | 3.14E-08 |
| 0.219791 | NA | NA | NA | NA | 0.404852 | 2 | -165.589 | 335.222 | 31.61127 | 2.85E-08 |
| 0.130424 | -0.11259 | NA | NA | NA | 0.736326 | 3 | -164.655 | 335.4002 | 31.78945 | 2.61E-08 |
| 0.097767 | NA | -0.40475 | 0.334902 | NA | 0.43512 | 4 | -163.704 | 335.5578 | 31.94708 | 2.41E-08 |
| -0.33444 | NA | NA | 0.135987 | NA | 0.399203 | 3 | -165.517 | 337.1227 | 33.51192 | 1.10E-08 |
| -0.66803 | -0.11667 | NA | 0.19562 | NA | 0.73909 | 4 | -164.511 | 337.1709 | 33.56015 | 1.08E-08 |
| 1.371903 | 0.097456 | -0.29223 | NA | NA | NA | 3 | -167.611 | 341.3117 | 37.70092 | 1.36E-09 |
| 0.4606 | 0.088197 | NA | NA | NA | NA | 2 | -168.694 | 341.4328 | 37.82208 | 1.28E-09 |
| 0.243362 | 0.092634 | -0.34729 | 0.319333 | NA | NA | 4 | -167.236 | 342.622 | 39.01122 | 7.05E-10 |
| -0.11771 | 0.085656 | NA | 0.14183 | NA | NA | 3 | -168.614 | 343.3173 | 39.70654 | 4.98E-10 |
| 0.72085 | NA | NA | NA | NA | NA | 1 | -171.954 | 345.9219 | 42.31116 | 1.35E-10 |
| 1.397429 | NA | -0.21111 | NA | NA | NA | 2 | -171.343 | 346.7308 | 43.12 | 9.03E-11 |
| -0.23255 | NA | -0.30231 | 0.465369 | NA | NA | 3 | -170.503 | 347.0953 | 43.48452 | 7.53E-11 |
| -0.48767 | NA | NA | 0.292534 | NA | NA | 2 | -171.584 | 347.2133 | 43.60256 | 7.10E-11 |

## **Table S2 – Model selection for grasses under irrigation, expansion, mean**

| (Intercept) | I((log(size))^2) | log(Nf) | log(Ng) | log(Nl + exp(-1)) | log(size) | df | logLik | AICc | delta | weight |
| --- | --- | --- | --- | --- | --- | --- | --- | --- | --- | --- |
| **1.943046** | **NA** | **NA** | **-0.3471** | **NA** | **0.616822** | **4** | **-242.726** | **493.6763** | **0** | **0.186668** |
| 2.011259 | 0.042232 | NA | -0.34986 | NA | 0.477404 | 5 | -242.093 | 494.525 | 0.848705 | 0.122117 |
| 1.845046 | NA | 0.116299 | -0.41172 | NA | 0.616254 | 5 | -242.098 | 494.5354 | 0.859089 | 0.121485 |
| 1.907469 | 0.050251 | 0.13854 | -0.42737 | NA | 0.450253 | 6 | -241.218 | 494.9134 | 1.237066 | 0.100565 |
| 0.52733 | NA | NA | NA | NA | 0.600047 | 3 | -244.653 | 495.4408 | 1.76445 | 0.077255 |
| 1.8961 | NA | NA | -0.36519 | 0.044992 | 0.61809 | 5 | -242.554 | 495.4478 | 1.771473 | 0.076984 |
| 1.956237 | 0.04672 | NA | -0.37416 | 0.05968 | 0.464267 | 6 | -241.796 | 496.0699 | 2.393626 | 0.056403 |
| 0.582871 | 0.041186 | NA | NA | NA | 0.463951 | 4 | -244.064 | 496.3529 | 2.676572 | 0.048962 |
| 1.811299 | NA | 0.111186 | -0.42355 | 0.036471 | 0.617307 | 6 | -241.986 | 496.4494 | 2.773095 | 0.046655 |
| 1.864031 | 0.053813 | 0.132864 | -0.44525 | 0.051728 | 0.439979 | 7 | -240.995 | 496.6292 | 2.952881 | 0.042644 |
| 0.390223 | NA | 0.044099 | NA | NA | 0.598647 | 4 | -244.556 | 497.3362 | 3.659866 | 0.029946 |
| 0.479663 | NA | NA | NA | 0.017761 | 0.600202 | 4 | -244.626 | 497.4775 | 3.80121 | 0.027903 |
| 0.396572 | 0.044638 | 0.061419 | NA | NA | 0.450593 | 5 | -243.878 | 498.0941 | 4.417835 | 0.0205 |
| 0.503274 | 0.043465 | NA | NA | 0.030804 | 0.456688 | 5 | -243.984 | 498.3076 | 4.631292 | 0.018425 |
| 0.3634 | NA | 0.041547 | NA | 0.012951 | 0.598841 | 5 | -244.542 | 499.4224 | 5.746107 | 0.010552 |
| 0.344968 | 0.046248 | 0.057112 | NA | 0.025027 | 0.445629 | 6 | -243.826 | 500.1288 | 6.452449 | 0.007412 |
| 2.021431 | 0.162438 | 0.189428 | -0.42801 | NA | NA | 5 | -246.423 | 503.1842 | 9.507889 | 0.001609 |
| 2.176168 | 0.160684 | NA | -0.31968 | NA | NA | 4 | -247.997 | 504.2186 | 10.54228 | 0.000959 |
| 1.951064 | 0.163969 | 0.178979 | -0.45532 | 0.079062 | NA | 6 | -245.923 | 504.3224 | 10.64605 | 0.000911 |
| 2.084298 | 0.16258 | NA | -0.35844 | 0.092057 | NA | 5 | -247.323 | 504.9859 | 11.30956 | 0.000653 |
| 0.863546 | 0.156668 | NA | NA | NA | NA | 3 | -249.547 | 505.2284 | 11.55205 | 0.000579 |
| 0.508355 | 0.156901 | 0.112231 | NA | NA | NA | 4 | -248.945 | 506.1138 | 12.43745 | 0.000372 |
| 0.689405 | 0.157646 | NA | NA | 0.063868 | NA | 4 | -249.218 | 506.661 | 12.98464 | 0.000283 |
| 0.398347 | 0.157678 | 0.102097 | NA | 0.052109 | NA | 5 | -248.729 | 507.7972 | 14.12092 | 0.00016 |
| 1.437759 | NA | NA | NA | NA | NA | 2 | -286.408 | 576.8833 | 83.20698 | 1.60E-19 |
| 1.123323 | NA | 0.099593 | NA | NA | NA | 3 | -286.092 | 578.3172 | 84.64089 | 7.79E-20 |
| 1.754381 | NA | NA | -0.07626 | NA | NA | 3 | -286.349 | 578.8315 | 85.15516 | 6.02E-20 |
| 1.41447 | NA | NA | NA | 0.008721 | NA | 3 | -286.404 | 578.9425 | 85.26616 | 5.70E-20 |
| 1.648514 | NA | 0.125857 | -0.14646 | NA | NA | 4 | -285.893 | 580.0103 | 86.33403 | 3.34E-20 |
| 1.129119 | NA | 0.100146 | NA | -0.00282 | NA | 4 | -286.091 | 580.407 | 86.73069 | 2.74E-20 |
| 1.738836 | NA | NA | -0.08202 | 0.014776 | NA | 4 | -286.337 | 580.8991 | 87.22284 | 2.14E-20 |
| 1.643626 | NA | 0.125127 | -0.14809 | 0.00523 | NA | 5 | -285.891 | 582.1217 | 88.44543 | 1.16E-20 |

## **Table S3 – Model selection for grasses under irrigation, expansion, s.d.**

| (Intercept) | I((log(size))^2) | log(Nf) | log(Ng) | log(Nl + exp(-1)) | log(size) | df | logLik | AICc | delta | weight |
| --- | --- | --- | --- | --- | --- | --- | --- | --- | --- | --- |
| **0.640883** | **NA** | **NA** | **NA** | **NA** | **0.064674** | **3** | **-142.912** | **291.9577** | **0** | **0.124982** |
| 0.677099 | 0.016892 | NA | NA | NA | NA | 3 | -143.088 | 292.31 | 0.35231 | 0.104796 |
| 0.518499 | NA | NA | NA | 0.045602 | 0.065074 | 4 | -142.37 | 292.9655 | 1.007757 | 0.075512 |
| 0.53858 | 0.01767 | NA | NA | 0.050803 | NA | 4 | -142.42 | 293.0654 | 1.107642 | 0.071833 |
| 0.739011 | NA | NA | NA | NA | NA | 2 | -144.762 | 293.5903 | 1.632522 | 0.055252 |
| 0.763783 | NA | NA | -0.03013 | NA | 0.066131 | 4 | -142.868 | 293.961 | 2.003217 | 0.045904 |
| 0.646916 | 0.004473 | NA | NA | NA | 0.049892 | 4 | -142.891 | 294.0062 | 2.048502 | 0.044877 |
| 0.620057 | NA | 0.006698 | NA | NA | 0.064462 | 4 | -142.905 | 294.0347 | 2.076954 | 0.044243 |
| 0.788794 | 0.017234 | NA | -0.0272 | NA | NA | 4 | -143.052 | 294.3294 | 2.371679 | 0.038181 |
| 0.632675 | 0.016921 | 0.014037 | NA | NA | NA | 4 | -143.058 | 294.3405 | 2.382787 | 0.037969 |
| 0.61985 | NA | NA | NA | 0.044622 | NA | 3 | -144.254 | 294.6416 | 2.683902 | 0.032662 |
| 0.712313 | NA | NA | -0.04997 | 0.049328 | 0.067521 | 5 | -142.253 | 294.8451 | 2.887323 | 0.029503 |
| 0.734149 | 0.018362 | NA | -0.05025 | 0.054756 | NA | 5 | -142.302 | 294.9426 | 2.984889 | 0.028099 |
| 0.522859 | 0.008026 | NA | NA | 0.048011 | 0.038573 | 5 | -142.304 | 294.9463 | 2.988566 | 0.028047 |
| 0.525051 | NA | -0.00234 | NA | 0.045873 | 0.06515 | 5 | -142.37 | 295.0781 | 3.120378 | 0.026258 |
| 0.526457 | 0.017671 | 0.004253 | NA | 0.050314 | NA | 5 | -142.418 | 295.1742 | 3.216469 | 0.025026 |
| 0.698997 | NA | 0.012674 | NA | NA | NA | 3 | -144.738 | 295.6095 | 3.651769 | 0.020131 |
| 0.743556 | NA | NA | -0.00109 | NA | NA | 3 | -144.762 | 295.6576 | 3.699818 | 0.019654 |
| 0.752596 | NA | 0.013276 | -0.03751 | NA | 0.066066 | 5 | -142.844 | 296.0267 | 4.068927 | 0.016341 |
| 0.771156 | 0.004564 | NA | -0.03043 | NA | 0.051062 | 5 | -142.846 | 296.0313 | 4.073599 | 0.016303 |
| 0.620763 | 0.004958 | 0.008622 | NA | NA | 0.048017 | 5 | -142.88 | 296.0983 | 4.140577 | 0.015766 |
| 0.771541 | 0.017429 | 0.021122 | -0.03928 | NA | NA | 5 | -142.991 | 296.3214 | 4.3637 | 0.014102 |
| 0.695133 | NA | NA | -0.01904 | 0.046028 | NA | 4 | -144.237 | 296.6982 | 4.740432 | 0.011681 |
| 0.608356 | NA | 0.004034 | NA | 0.044157 | NA | 4 | -144.251 | 296.7275 | 4.769748 | 0.011511 |
| 0.723221 | 0.008475 | NA | -0.0516 | 0.051993 | 0.039618 | 6 | -142.179 | 296.8346 | 4.876864 | 0.010911 |
| 0.707409 | NA | 0.00643 | -0.05334 | 0.048836 | 0.067476 | 6 | -142.247 | 296.972 | 5.014272 | 0.010186 |
| 0.723713 | 0.018471 | 0.01402 | -0.05784 | 0.053738 | NA | 6 | -142.275 | 297.0271 | 5.06941 | 0.009909 |
| 0.521845 | 0.008044 | 0.000366 | NA | 0.047974 | 0.038502 | 6 | -142.304 | 297.0846 | 5.126817 | 0.009629 |
| 0.731527 | NA | 0.014301 | -0.00907 | NA | NA | 4 | -144.734 | 297.693 | 5.735269 | 0.007103 |
| 0.759395 | 0.005473 | 0.015698 | -0.03921 | NA | 0.047986 | 6 | -142.813 | 298.1035 | 6.145719 | 0.005785 |
| 0.689081 | NA | 0.007953 | -0.02324 | 0.045421 | NA | 5 | -144.228 | 298.7954 | 6.837713 | 0.004093 |
| 0.71624 | 0.009012 | 0.01006 | -0.05698 | 0.051391 | 0.037779 | 7 | -142.165 | 298.9701 | 7.012402 | 0.003751 |

## **Table S4 – Model selection for grasses under irrigation, colonisation, mean**

| (Intercept) | log(Nf) | log(Ng) | log(Nl + exp(-1)) | df | logLik | AICc | delta | weight |
| --- | --- | --- | --- | --- | --- | --- | --- | --- |
| -2.08258 | NA | 0.675944 | NA | 3 | -76.9644 | 160.4186 | 0 | 0.277519 |
| **0.729227** | **NA** | **NA** | **NA** | **2** | **-78.1397** | **160.5193** | **0.100766** | **0.263883** |
| 0.269965 | 0.146273 | NA | NA | 3 | -77.848 | 162.1859 | 1.767326 | 0.114689 |
| 0.366744 | NA | NA | 0.118967 | 3 | -77.9529 | 162.3955 | 1.976971 | 0.103276 |
| -2.12797 | NA | 0.647679 | 0.053488 | 4 | -76.9271 | 162.6876 | 2.269023 | 0.089244 |
| -2.09094 | -0.00459 | 0.681418 | NA | 4 | -76.9642 | 162.7617 | 2.343098 | 0.085999 |
| 0.073947 | 0.124501 | NA | 0.086769 | 4 | -77.7543 | 164.3419 | 3.923299 | 0.039026 |
| -2.1566 | -0.01471 | 0.664094 | 0.055633 | 5 | -76.9249 | 165.1265 | 4.707884 | 0.026363 |

## **Table S5 – Model selection for grasses under irrigation, colonisation, s.d.**

| (Intercept) | log(Nf) | log(Ng) | log(Nl + exp(-1)) | df | logLik | AICc | delta | weight |
| --- | --- | --- | --- | --- | --- | --- | --- | --- |
| **0.782758** | **NA** | **NA** | **NA** | **2** | **-57.0701** | **118.3802** | **0** | **0.426444** |
| 0.886922 | -0.03318 | NA | NA | 3 | -57.0371 | 120.5639 | 2.183675 | 0.143114 |
| 0.570663 | NA | 0.050987 | NA | 3 | -57.0556 | 120.601 | 2.220817 | 0.140481 |
| 0.840977 | NA | NA | -0.01911 | 3 | -57.0595 | 120.6088 | 2.228524 | 0.139941 |
| 0.460595 | -0.06042 | 0.123049 | NA | 4 | -56.9748 | 122.783 | 4.402732 | 0.047187 |
| 0.912355 | -0.03035 | NA | -0.01126 | 4 | -57.0336 | 122.9005 | 4.520299 | 0.044493 |
| 0.592415 | NA | 0.064532 | -0.02563 | 4 | -57.0375 | 122.9083 | 4.528096 | 0.04432 |
| 0.480989 | -0.05727 | 0.12843 | -0.01728 | 5 | -56.9668 | 125.2101 | 6.829907 | 0.014021 |

## **Table S6 – Model selection for legumes under irrigation, persistence**

| (Intercept) | I((log(size))^2) | log(Nf) | log(Ng) | log(Nl + exp(-1)) | log(size) | df | logLik | AICc | delta | weight |
| --- | --- | --- | --- | --- | --- | --- | --- | --- | --- | --- |
| 5.487029 | -0.38151 | NA | NA | -1.50491 | 1.297155 | 4 | -60.4016 | 129.111 | 0 | 0.352733 |
| **6.860951** | **-0.40944** | **-0.47447** | **NA** | **-1.49085** | **1.433169** | **5** | **-59.4581** | **129.3814** | **0.270397** | **0.308127** |
| 3.950385 | -0.4068 | -0.65394 | 0.849021 | -1.50254 | 1.438615 | 6 | -58.9089 | 130.4741 | 1.363157 | 0.178419 |
| 4.31167 | -0.3784 | NA | 0.282852 | -1.50224 | 1.286641 | 5 | -60.3301 | 131.1253 | 2.014357 | 0.128835 |
| 6.913465 | NA | -0.49561 | NA | -1.44702 | 0.33916 | 4 | -64.611 | 137.5296 | 8.418668 | 0.00524 |
| 5.369037 | NA | NA | NA | -1.44326 | 0.29475 | 3 | -65.8061 | 137.7953 | 8.684374 | 0.004588 |
| 5.097032 | NA | NA | NA | -1.25188 | NA | 2 | -66.9346 | 137.96 | 8.849081 | 0.004226 |
| 3.747027 | NA | -0.687 | 0.920087 | -1.45883 | 0.352803 | 5 | -63.8854 | 138.236 | 9.125002 | 0.003681 |
| 6.314582 | NA | -0.41397 | NA | -1.22079 | NA | 3 | -66.0644 | 138.312 | 9.201055 | 0.003544 |
| 3.437144 | NA | -0.5839 | 0.826176 | -1.2186 | NA | 4 | -65.4475 | 139.2027 | 10.09176 | 0.00227 |
| 4.977191 | -0.03831 | NA | NA | -1.18113 | NA | 3 | -66.733 | 139.6493 | 10.5383 | 0.001816 |
| 4.020907 | NA | NA | 0.321921 | -1.43858 | 0.29426 | 4 | -65.7015 | 139.7107 | 10.5997 | 0.001761 |
| 3.759879 | NA | NA | 0.320169 | -1.24835 | NA | 3 | -66.8261 | 139.8354 | 10.72444 | 0.001654 |
| 6.171053 | -0.03013 | -0.39688 | NA | -1.16659 | NA | 4 | -65.9433 | 140.1943 | 11.08338 | 0.001383 |
| 3.383315 | -0.02605 | -0.56512 | 0.806484 | -1.17223 | NA | 5 | -65.3582 | 141.1814 | 12.07047 | 0.000844 |
| 3.66955 | -0.03793 | NA | 0.313808 | -1.17888 | NA | 4 | -66.6287 | 141.5651 | 12.45418 | 0.000697 |
| 2.541188 | -0.43705 | -0.50449 | NA | NA | 1.14575 | 4 | -68.991 | 146.2896 | 17.17863 | 6.56E-05 |
| 1.0435 | -0.39804 | NA | NA | NA | 0.971962 | 3 | -70.2704 | 146.7239 | 17.61298 | 5.28E-05 |
| 1.503151 | -0.4283 | -0.56017 | 0.297562 | NA | 1.116371 | 5 | -68.8672 | 148.1996 | 19.08865 | 2.53E-05 |
| 1.083593 | -0.39835 | NA | -0.00985 | NA | 0.973112 | 4 | -70.2702 | 148.8481 | 19.73717 | 1.83E-05 |
| 1.435184 | -0.12578 | NA | NA | NA | NA | 2 | -73.6713 | 151.4334 | 22.32247 | 5.01E-06 |
| 2.751415 | -0.11598 | -0.42137 | NA | NA | NA | 3 | -72.6387 | 151.4606 | 22.34962 | 4.95E-06 |
| 1.219816 | -0.11517 | -0.50814 | 0.437893 | NA | NA | 4 | -72.368 | 153.0437 | 23.9327 | 2.24E-06 |
| 0.974163 | -0.1259 | NA | 0.111699 | NA | NA | 3 | -73.652 | 153.4872 | 24.37622 | 1.80E-06 |
| 2.654593 | NA | -0.49223 | NA | NA | NA | 2 | -74.7043 | 153.4995 | 24.38854 | 1.78E-06 |
| 1.08876 | NA | NA | NA | NA | NA | 1 | -76.1887 | 154.4074 | 25.29644 | 1.13E-06 |
| 1.098082 | NA | -0.5785 | 0.443927 | NA | NA | 3 | -74.4052 | 154.9937 | 25.8827 | 8.45E-07 |
| 2.668344 | NA | -0.48545 | NA | NA | -0.03167 | 3 | -74.6885 | 155.5602 | 26.44925 | 6.37E-07 |
| 1.169051 | NA | NA | NA | NA | -0.07177 | 2 | -76.1062 | 156.3033 | 27.19238 | 4.39E-07 |
| 0.710857 | NA | NA | 0.091521 | NA | NA | 2 | -76.1748 | 156.4404 | 27.32948 | 4.10E-07 |
| 1.098709 | NA | -0.57183 | 0.448324 | NA | -0.03576 | 4 | -74.385 | 157.0777 | 27.96671 | 2.98E-07 |
| 0.740365 | NA | NA | 0.104306 | NA | -0.07356 | 3 | -76.0883 | 158.3599 | 29.2489 | 1.57E-07 |

## **Table S7 – Model selection for legumes under irrigation, expansion, mean**

| (Intercept) | I((log(size))^2) | log(Nf) | log(Ng) | log(Nl + exp(-1)) | log(size) | | df | logLik | AICc | delta | weight |
| --- | --- | --- | --- | --- | --- | --- | --- | --- | --- | --- | --- |
| **1.044617** | **NA** | **NA** | **NA** | **-0.19373** | | **0.597585** | **4** | **-136.395** | **281.2059** | **0** | **0.15725** |
| 0.57963 | NA | NA | NA | NA | 0.521598 | | 3 | -137.716 | 281.6797 | 0.473764 | 0.124084 |
| -0.1343 | NA | NA | 0.312217 | -0.23326 | 0.597022 | | 5 | -135.647 | 281.9261 | 0.720184 | 0.1097 |
| 0.953468 | -0.10646 | NA | NA | -0.19676 | 0.895506 | | 5 | -135.733 | 282.0981 | 0.892178 | 0.10066 |
| 0.48439 | -0.10302 | NA | NA | NA | 0.80872 | | 4 | -137.113 | 282.6426 | 1.436633 | 0.076671 |
| 0.790964 | NA | 0.096759 | NA | -0.2041 | 0.584579 | | 5 | -136.14 | 282.9122 | 1.706264 | 0.067001 |
| -0.10566 | -0.09507 | NA | 0.283077 | -0.23227 | 0.863121 | | 6 | -135.119 | 283.1322 | 1.926259 | 0.060022 |
| -0.16888 | NA | NA | 0.183468 | NA | 0.512157 | | 4 | -137.447 | 283.3109 | 2.104971 | 0.054891 |
| 0.38274 | NA | 0.068397 | NA | NA | 0.50953 | | 4 | -137.591 | 283.5979 | 2.39199 | 0.047553 |
| -0.21403 | NA | 0.066404 | 0.287231 | -0.23721 | 0.588142 | | 6 | -135.531 | 283.955 | 2.749068 | 0.039778 |
| 0.744189 | -0.10075 | 0.081697 | NA | -0.20535 | 0.86855 | | 6 | -135.552 | 283.9968 | 2.79092 | 0.038954 |
| -0.13968 | -0.09646 | NA | 0.154452 | NA | 0.782505 | | 5 | -136.923 | 284.4768 | 3.270879 | 0.030643 |
| 0.334218 | -0.09919 | 0.053397 | NA | NA | 0.788625 | | 5 | -137.036 | 284.7044 | 3.498467 | 0.027347 |
| -0.17285 | -0.09201 | 0.055183 | 0.26325 | -0.23559 | 0.847181 | | 7 | -135.039 | 285.2813 | 4.075394 | 0.020494 |
| -0.22753 | NA | 0.048483 | 0.163633 | NA | 0.504624 | | 5 | -137.387 | 285.4054 | 4.199473 | 0.019261 |
| -0.18517 | -0.09442 | 0.037094 | 0.13989 | NA | 0.771017 | | 6 | -136.887 | 286.6681 | 5.462187 | 0.010245 |
| 0.826739 | 0.15132 | NA | NA | NA | NA | | 3 | -141.166 | 288.5787 | 7.372752 | 0.003941 |
| 1.152627 | 0.166683 | NA | NA | -0.12677 | NA | | 4 | -140.623 | 289.6621 | 8.456178 | 0.002293 |
| -0.24086 | 0.148479 | NA | 0.259602 | NA | NA | | 4 | -140.656 | 289.7291 | 8.523133 | 0.002217 |
| 0.462914 | 0.145611 | 0.122432 | NA | NA | NA | | 4 | -140.78 | 289.9776 | 8.771652 | 0.001958 |
| -0.22299 | 0.168635 | NA | 0.365181 | -0.17585 | NA | | 5 | -139.682 | 289.9955 | 8.78962 | 0.001941 |
| 0.763848 | 0.162144 | 0.147541 | NA | -0.14609 | NA | | 5 | -140.069 | 290.7698 | 9.563881 | 0.001318 |
| -0.35301 | 0.144519 | 0.094553 | 0.218551 | NA | NA | | 5 | -140.437 | 291.5063 | 10.30036 | 0.000912 |
| -0.35635 | 0.164918 | 0.113193 | 0.321403 | -0.18479 | NA | | 6 | -139.364 | 291.6226 | 10.41664 | 0.00086 |
| 0.428393 | NA | 0.231847 | NA | NA | NA | | 3 | -148.903 | 304.0536 | 22.84766 | 1.72E-06 |
| 1.139726 | NA | NA | NA | NA | NA | | 2 | -150.112 | 304.3462 | 23.14029 | 1.49E-06 |
| -0.31302 | NA | NA | 0.351324 | NA | NA | | 3 | -149.323 | 304.8931 | 23.68722 | 1.13E-06 |
| -0.54345 | NA | 0.197653 | 0.260396 | NA | NA | | 4 | -148.487 | 305.3916 | 24.18571 | 8.81E-07 |
| 0.886515 | NA | NA | NA | 0.089748 | NA | | 3 | -149.842 | 305.9316 | 24.7257 | 6.72E-07 |
| 0.321805 | NA | 0.218377 | NA | 0.052427 | NA | | 4 | -148.813 | 306.0428 | 24.83693 | 6.36E-07 |
| -0.31527 | NA | NA | 0.318314 | 0.04918 | NA | | 4 | -149.248 | 306.9122 | 25.70626 | 4.12E-07 |
| -0.53928 | NA | 0.193077 | 0.245402 | 0.025474 | NA | | 5 | -148.467 | 307.5665 | 26.36057 | 2.97E-07 |

## **Table S8 – Model selection for legumes under irrigation, expansion, s.d.**

| (Intercept) | I((log(size))^2) | log(Nf) | log(Ng) | log(Nl + exp(-1)) | log(size) | df | logLik | AICc | delta | weight |
| --- | --- | --- | --- | --- | --- | --- | --- | --- | --- | --- |
| **0.574748** | **-0.08649** | **NA** | **NA** | **NA** | **0.328548** | **4** | **-82.0032** | **172.423** | **0** | **0.103674** |
| 0.654708 | NA | NA | NA | NA | 0.087492 | 3 | -83.2611 | 172.7697 | 0.346688 | 0.087174 |
| 0.748657 | NA | NA | NA | NA | NA | 2 | -84.41 | 172.9425 | 0.519519 | 0.079957 |
| 0.782958 | -0.08802 | NA | NA | -0.08734 | 0.367069 | 5 | -81.1982 | 173.028 | 0.605023 | 0.076611 |
| 1.242255 | -0.0935 | NA | -0.1652 | NA | 0.356588 | 5 | -81.3517 | 173.335 | 0.912035 | 0.065709 |
| 0.858314 | NA | NA | NA | -0.08483 | 0.120765 | 4 | -82.5205 | 173.4577 | 1.034714 | 0.061799 |
| 1.21395 | NA | NA | -0.13708 | NA | 0.094545 | 4 | -82.8189 | 174.0545 | 1.631504 | 0.045856 |
| 0.713829 | 0.016838 | NA | NA | NA | NA | 3 | -84.0358 | 174.319 | 1.895982 | 0.040176 |
| 0.666317 | -0.08882 | -0.03256 | NA | NA | 0.340801 | 5 | -81.9184 | 174.4684 | 2.045399 | 0.037284 |
| 1.187343 | NA | NA | -0.10609 | NA | NA | 3 | -84.1471 | 174.5417 | 2.118692 | 0.035942 |
| 1.252739 | -0.09307 | NA | -0.12556 | -0.07159 | 0.381434 | 6 | -80.8435 | 174.5806 | 2.157555 | 0.03525 |
| 0.826364 | NA | NA | NA | -0.02754 | NA | 3 | -84.3169 | 174.8812 | 2.458166 | 0.030331 |
| 0.709768 | NA | -0.01913 | NA | NA | 0.090866 | 4 | -83.2323 | 174.8813 | 2.458278 | 0.030329 |
| 0.71791 | NA | 0.010021 | NA | NA | NA | 3 | -84.4018 | 175.0511 | 2.628097 | 0.02786 |
| 0.836306 | -0.08947 | -0.02083 | NA | -0.08515 | 0.373941 | 6 | -81.1635 | 175.2207 | 2.797653 | 0.025596 |
| 1.224706 | NA | NA | -0.09703 | -0.07255 | 0.120939 | 5 | -82.3119 | 175.2553 | 2.832291 | 0.025156 |
| 1.259354 | -0.09427 | -0.01394 | -0.15973 | NA | 0.360907 | 6 | -81.3367 | 175.567 | 3.144039 | 0.021525 |
| 0.877844 | NA | -0.00745 | NA | -0.08403 | 0.121766 | 5 | -82.5162 | 175.6639 | 3.240885 | 0.020508 |
| 0.864593 | 0.023945 | NA | NA | -0.05865 | NA | 4 | -83.6763 | 175.7692 | 3.346214 | 0.019456 |
| 1.19615 | 0.018121 | NA | -0.11728 | NA | NA | 4 | -83.7141 | 175.8448 | 3.421831 | 0.018734 |
| 1.217063 | NA | -0.00257 | -0.13602 | NA | 0.094945 | 5 | -82.8184 | 176.2684 | 3.845416 | 0.015158 |
| 0.721932 | 0.016965 | -0.00273 | NA | NA | NA | 4 | -84.0352 | 176.487 | 4.064046 | 0.013589 |
| 1.157622 | NA | 0.025493 | -0.11782 | NA | NA | 4 | -84.097 | 176.6107 | 4.187689 | 0.012774 |
| 1.188046 | NA | NA | -0.0958 | -0.01533 | NA | 4 | -84.1206 | 176.6579 | 4.234888 | 0.012476 |
| 1.263071 | -0.09354 | -0.00849 | -0.12251 | -0.07108 | 0.383885 | 7 | -80.8379 | 176.8801 | 4.457054 | 0.011164 |
| 0.78012 | NA | 0.017883 | NA | -0.0306 | NA | 4 | -84.2919 | 177.0004 | 4.577394 | 0.010512 |
| 1.221199 | NA | 0.002921 | -0.09813 | -0.07272 | 0.120549 | 6 | -82.3112 | 177.516 | 5.093034 | 0.008123 |
| 1.200889 | 0.023468 | NA | -0.08928 | -0.04665 | NA | 5 | -83.5039 | 177.6395 | 5.216477 | 0.007637 |
| 0.84477 | 0.023714 | 0.007523 | NA | -0.05963 | NA | 5 | -83.6719 | 177.9753 | 5.552285 | 0.006457 |
| 1.180787 | 0.017579 | 0.012952 | -0.12291 | NA | NA | 5 | -83.7014 | 178.0344 | 5.611433 | 0.006268 |
| 1.154535 | NA | 0.028884 | -0.10671 | -0.01888 | NA | 5 | -84.0576 | 178.7469 | 6.323863 | 0.00439 |
| 1.179918 | 0.022884 | 0.017799 | -0.09616 | -0.04806 | NA | 6 | -83.4801 | 179.8539 | 7.430892 | 0.002524 |

## **Table S9 – Model selection for legumes under irrigation, colonisation, mean**

| (Intercept) | log(Nf) | log(Ng) | log(Nl + exp(-1)) | df | logLik | AICc | delta | weight |
| --- | --- | --- | --- | --- | --- | --- | --- | --- |
| **0.427788** | **NA** | **NA** | **NA** | **2** | **-12.4288** | **29.71472** | **0** | **0.464619** |
| -0.21364 | 0.211161 | NA | NA | 3 | -11.7508 | 31.34779 | 1.633076 | 0.205342 |
| -0.3846 | NA | 0.197864 | NA | 3 | -12.2664 | 32.37892 | 2.664207 | 0.122623 |
| 0.458758 | NA | NA | -0.01181 | 3 | -12.4274 | 32.70095 | 2.986235 | 0.104386 |
| -0.71456 | 0.198787 | 0.131159 | NA | 4 | -11.6764 | 34.68607 | 4.971351 | 0.038688 |
| -0.30565 | 0.215217 | NA | 0.030394 | 4 | -11.7411 | 34.81558 | 5.100866 | 0.036263 |
| -0.94927 | NA | 0.277857 | 0.090103 | 4 | -12.2107 | 35.75474 | 6.040025 | 0.022674 |
| -1.42761 | 0.204548 | 0.228884 | 0.112254 | 5 | -11.584 | 38.62264 | 8.907925 | 0.005405 |

## **Table S10 – Model selection for legumes under irrigation, colonisation, s.d.**

| (Intercept) | log(Nf) | log(Ng) | log(Nl + exp(-1)) | df | logLik | AICc | delta | weight |
| --- | --- | --- | --- | --- | --- | --- | --- | --- |
| **0.452952** | **NA** | **NA** | **NA** | **2** | **1.776799** | **1.303546** | **0** | **0.384106** |
| 0.083261 | NA | NA | 0.141008 | 3 | 2.899545 | 2.047065 | 0.743519 | 0.264848 |
| 0.311555 | 0.046549 | NA | NA | 3 | 1.946913 | 3.952328 | 2.648783 | 0.102159 |
| 0.770955 | NA | -0.07745 | NA | 3 | 1.908917 | 4.028321 | 2.724775 | 0.09835 |
| -0.15515 | 0.067124 | NA | 0.154172 | 4 | 3.29795 | 4.737433 | 3.433887 | 0.068991 |
| -0.27544 | NA | 0.070785 | 0.166972 | 4 | 2.984236 | 5.364862 | 4.061316 | 0.050414 |
| 0.678639 | 0.055616 | -0.09611 | NA | 4 | 2.148737 | 7.03586 | 5.732314 | 0.021862 |
| -0.42638 | 0.064544 | 0.055332 | 0.173962 | 5 | 3.35148 | 8.751586 | 7.44804 | 0.009271 |

## **Table S11 – Model selection for forbs under irrigation, persistence**

| (Intercept) | I((log(size))^2) | log(Nf) | log(Ng) | log(Nl + exp(-1)) | log(size) | df | logLik | AICc | delta | weight |
| --- | --- | --- | --- | --- | --- | --- | --- | --- | --- | --- |
| 3.821004 | 0.108906 | -0.82922 | NA | -0.26985 | 0.191941 | 5 | -251.085 | 512.3181 | 0 | 0.168396 |
| 2.292898 | NA | -0.79895 | 0.406474 | -0.32807 | 0.276588 | 5 | -251.086 | 512.3206 | 0.002515 | 0.168184 |
| 2.778272 | 0.096427 | -0.92029 | 0.360219 | -0.31848 | 0.222769 | 6 | -250.17 | 512.548 | 0.22989 | 0.15011 |
| **3.42127** | **NA** | **-0.67772** | **NA** | **-0.27368** | **0.250657** | **4** | **-252.283** | **512.6652** | **0.347152** | **0.141562** |
| 3.800954 | 0.138702 | -0.81224 | NA | -0.26417 | NA | 4 | -252.284 | 512.6677 | 0.349632 | 0.141387 |
| 2.997597 | 0.132809 | -0.87987 | 0.275839 | -0.29998 | NA | 5 | -251.731 | 513.6102 | 1.292103 | 0.088258 |
| 3.223914 | NA | -0.58792 | NA | -0.26816 | NA | 3 | -254.503 | 515.0648 | 2.74671 | 0.042647 |
| 2.316704 | NA | -0.67722 | 0.320458 | -0.30929 | NA | 4 | -253.74 | 515.5794 | 3.261351 | 0.032971 |
| 3.363025 | 0.113288 | -0.91998 | NA | NA | 0.18176 | 4 | -254.451 | 517.0006 | 4.682535 | 0.016201 |
| 3.354631 | 0.141712 | -0.90245 | NA | NA | NA | 3 | -255.532 | 517.1225 | 4.804403 | 0.015243 |
| 2.94552 | NA | -0.76542 | NA | NA | 0.244221 | 3 | -255.758 | 517.5754 | 5.257292 | 0.012154 |
| 3.072429 | 0.110037 | -0.94775 | 0.093273 | NA | 0.189141 | 5 | -254.374 | 518.8974 | 6.579354 | 0.006275 |
| 3.235141 | 0.140854 | -0.91354 | 0.038284 | NA | NA | 4 | -255.518 | 519.136 | 6.817882 | 0.00557 |
| 2.537237 | NA | -0.81223 | 0.136391 | NA | 0.251987 | 4 | -255.59 | 519.2792 | 6.961131 | 0.005185 |
| 2.760953 | NA | -0.67533 | NA | NA | NA | 2 | -257.872 | 519.7742 | 7.456144 | 0.004048 |
| 2.524513 | NA | -0.70064 | 0.077973 | NA | NA | 3 | -257.817 | 521.6927 | 9.374657 | 0.001551 |
| 1.524412 | NA | NA | NA | -0.34779 | NA | 2 | -261.821 | 527.6724 | 15.35428 | 7.80E-05 |
| 1.494988 | NA | NA | NA | -0.35593 | 0.120855 | 3 | -261.259 | 528.5776 | 16.25951 | 4.96E-05 |
| 1.900435 | NA | NA | -0.10139 | -0.33308 | NA | 3 | -261.733 | 529.5262 | 17.20809 | 3.09E-05 |
| 1.536244 | -0.02156 | NA | NA | -0.34397 | NA | 3 | -261.741 | 529.5418 | 17.22374 | 3.06E-05 |
| 1.513285 | -0.05106 | NA | NA | -0.34971 | 0.160187 | 4 | -260.879 | 529.8566 | 17.53856 | 2.62E-05 |
| 1.843533 | NA | NA | -0.09385 | -0.34223 | 0.119169 | 4 | -261.184 | 530.4671 | 18.14902 | 1.93E-05 |
| 1.828846 | -0.01632 | NA | -0.07972 | -0.33327 | NA | 4 | -261.691 | 531.482 | 19.16386 | 1.16E-05 |
| 1.609941 | -0.04892 | NA | -0.02629 | -0.34608 | 0.158082 | 5 | -260.874 | 531.896 | 19.57789 | 9.44E-06 |
| 2.039769 | NA | NA | -0.36147 | NA | NA | 2 | -266.794 | 537.618 | 25.29988 | 5.40E-07 |
| 0.556735 | NA | NA | NA | NA | NA | 1 | -268.244 | 538.4984 | 26.18029 | 3.48E-07 |
| 2.003249 | NA | NA | -0.36176 | NA | 0.08745 | 3 | -266.491 | 539.0416 | 26.72354 | 2.65E-07 |
| 1.973557 | -0.01626 | NA | -0.34126 | NA | NA | 3 | -266.752 | 539.5628 | 27.24473 | 2.04E-07 |
| 0.60053 | -0.04242 | NA | NA | NA | NA | 2 | -267.927 | 539.8833 | 27.5652 | 1.74E-07 |
| 0.519089 | NA | NA | NA | NA | 0.087901 | 2 | -267.944 | 539.9184 | 27.60035 | 1.71E-07 |
| 0.566224 | -0.06887 | NA | NA | NA | 0.141953 | 3 | -267.237 | 540.5339 | 28.21584 | 1.26E-07 |
| 1.825052 | -0.04067 | NA | -0.31162 | NA | 0.119607 | 4 | -266.272 | 540.6421 | 28.32396 | 1.19E-07 |

## **Table S12 – Model selection for forbs under irrigation, expansion, mean**

| (Intercept) | I((log(size))^2) | log(Nf) | log(Ng) | log(Nl + exp(-1)) | log(size) | df | logLik | AICc | delta | weight |
| --- | --- | --- | --- | --- | --- | --- | --- | --- | --- | --- |
| 0.07814 | 0.126839685 | 0.12239 | NA | -0.05157 | NA | 5 | -195.651 | 401.5383 | 0 | 0.143891 |
| 0.06455 | 0.112379681 | 0.122216 | NA | -0.05177 | 0.061359 | 6 | -194.621 | 401.5736 | 0.035394 | 0.141367 |
| 0.03544 | 0.128952984 | 0.092852 | NA | NA | NA | 4 | -197.358 | 402.8723 | 1.334084 | 0.073848 |
| 0.021772 | 0.11459699 | 0.092563 | NA | NA | 0.060953 | 5 | -196.354 | 402.9452 | 1.406908 | 0.071208 |
| 0.306626 | 0.147126336 | NA | NA | NA | NA | 3 | -198.486 | 403.0664 | 1.528153 | 0.067019 |
| 0.292064 | 0.132663209 | NA | NA | NA | 0.061167 | 4 | -197.485 | 403.1263 | 1.588062 | 0.065041 |
| **0.401734** | **0.149831244** | **NA** | **NA** | **-0.03803** | **NA** | **4** | **-197.507** | **403.1702** | **1.631969** | **0.063629** |
| 0.387646 | 0.135301013 | NA | NA | -0.03825 | 0.061518 | 5 | -196.486 | 403.2079 | 1.669665 | 0.062441 |
| 0.189016 | 0.128092033 | 0.126308 | -0.03454 | -0.04538 | NA | 6 | -195.539 | 403.4092 | 1.870968 | 0.056462 |
| 0.304998 | 0.131273356 | 0.110199 | -0.08026 | NA | NA | 5 | -196.625 | 403.4859 | 1.947626 | 0.054339 |
| 0.126629 | 0.113478577 | 0.124401 | -0.01922 | -0.04832 | 0.059653 | 7 | -194.587 | 403.6175 | 2.079264 | 0.050878 |
| 0.254474 | 0.118005405 | 0.10748 | -0.06888 | NA | 0.054937 | 6 | -195.821 | 403.9733 | 2.435085 | 0.042585 |
| 0.522385 | 0.150977335 | NA | -0.05408 | NA | NA | 4 | -198.139 | 404.4351 | 2.896888 | 0.033805 |
| 0.463945 | 0.136595186 | NA | -0.04286 | NA | 0.057445 | 5 | -197.269 | 404.7738 | 3.235581 | 0.028539 |
| 0.457466 | 0.15074531 | NA | -0.01588 | -0.03498 | NA | 5 | -197.483 | 405.202 | 3.663704 | 0.023039 |
| 0.388987 | 0.135330823 | NA | -0.00038 | -0.03818 | 0.061484 | 6 | -196.486 | 405.3037 | 3.765434 | 0.021897 |
| -0.33056 | NA | 0.27632 | NA | -0.05857 | 0.150005 | 5 | -205.744 | 421.7243 | 20.18603 | 5.95E-06 |
| -0.46099 | NA | 0.268048 | 0.043064 | -0.06615 | 0.151884 | 6 | -205.581 | 423.4937 | 21.95548 | 2.46E-06 |
| -0.38792 | NA | 0.246137 | NA | NA | 0.151528 | 4 | -207.784 | 423.7243 | 22.18603 | 2.19E-06 |
| -0.31592 | NA | 0.252498 | -0.02249 | NA | 0.150443 | 5 | -207.731 | 425.6975 | 24.15928 | 8.16E-07 |
| -0.44242 | NA | 0.336387 | NA | -0.06059 | NA | 4 | -212.534 | 433.2247 | 31.68649 | 1.89E-08 |
| -0.50323 | NA | 0.332919 | 0.019864 | -0.0641 | NA | 5 | -212.501 | 435.2378 | 33.69954 | 6.92E-09 |
| -0.50295 | NA | 0.305785 | NA | NA | NA | 3 | -214.606 | 435.3055 | 33.76724 | 6.69E-09 |
| -0.36224 | NA | 0.317252 | -0.04346 | NA | NA | 4 | -214.417 | 436.99 | 35.45173 | 2.88E-09 |
| 0.352814 | NA | NA | NA | NA | 0.203718 | 3 | -217.376 | 440.8457 | 39.30745 | 4.19E-10 |
| -0.04758 | NA | NA | 0.1285 | -0.04757 | 0.206169 | 5 | -215.675 | 441.5863 | 40.04809 | 2.90E-10 |
| 0.040937 | NA | NA | 0.076957 | NA | 0.202814 | 4 | -216.725 | 441.6072 | 40.06891 | 2.87E-10 |
| 0.407565 | NA | NA | NA | -0.02164 | 0.20552 | 4 | -217.1 | 442.3571 | 40.81885 | 1.97E-10 |
| 0.447402 | NA | NA | NA | NA | NA | 2 | -229.612 | 463.2712 | 61.73298 | 5.66E-15 |
| 0.107841 | NA | NA | 0.083675 | NA | NA | 3 | -228.912 | 463.9169 | 62.37862 | 4.10E-15 |
| 0.038329 | NA | NA | 0.124752 | -0.03783 | NA | 4 | -228.306 | 464.7687 | 63.23048 | 2.68E-15 |
| 0.479978 | NA | NA | NA | -0.01268 | NA | 3 | -229.526 | 465.1453 | 63.60708 | 2.22E-15 |

## **Table S13 – Model selection for forbs under irrigation, expansion, s.d.**

| (Intercept) | I((log(size))^2) | log(Nf) | log(Ng) | log(Nl + exp(-1)) | log(size) | df | logLik | AICc | delta | weight |
| --- | --- | --- | --- | --- | --- | --- | --- | --- | --- | --- |
| 0.492926 | 0.036814 | NA | NA | -0.03007 | -0.03294 | 5 | -29.479 | 69.19416 | 0 | 0.119297 |
| 0.485382 | 0.029033 | NA | NA | -0.03019 | NA | 4 | -30.5366 | 69.23013 | 0.035972 | 0.117171 |
| **0.366867** | **0.027871** | **0.047684** | **NA** | **-0.03535** | **-0.03301** | **6** | **-28.4564** | **69.24483** | **0.05067** | **0.116313** |
| 0.359557 | 0.020093 | 0.04759 | NA | -0.03546 | NA | 5 | -29.5264 | 69.28898 | 0.094826 | 0.113773 |
| 0.277094 | NA | 0.08149 | NA | -0.03688 | NA | 4 | -31.14 | 70.43693 | 1.242772 | 0.064086 |
| 0.443629 | 0.02923 | 0.050385 | -0.02377 | -0.03108 | -0.03511 | 7 | -28.2681 | 70.9807 | 1.786544 | 0.04883 |
| 0.549891 | 0.038081 | NA | -0.01614 | -0.02697 | -0.03437 | 6 | -29.3914 | 71.1148 | 1.920641 | 0.045663 |
| 0.406905 | 0.020628 | 0.049263 | -0.01475 | -0.03281 | NA | 6 | -29.4528 | 71.23765 | 2.043494 | 0.042943 |
| 0.511607 | 0.029463 | NA | -0.00747 | -0.02876 | NA | 5 | -30.5176 | 71.2714 | 2.07724 | 0.042224 |
| 0.417784 | 0.03474 | NA | NA | NA | -0.03322 | 4 | -31.6993 | 71.55538 | 2.361225 | 0.036635 |
| 0.409876 | 0.026886 | NA | NA | NA | NA | 3 | -32.7565 | 71.60682 | 2.412664 | 0.035705 |
| 0.602844 | 0.038974 | NA | -0.04614 | NA | -0.03723 | 5 | -30.8009 | 71.83803 | 2.643874 | 0.031807 |
| 0.268877 | NA | 0.085903 | NA | -0.03703 | -0.01102 | 5 | -30.9958 | 72.22787 | 3.033717 | 0.026174 |
| 0.564974 | 0.029654 | NA | -0.03887 | NA | NA | 4 | -32.1139 | 72.38458 | 3.190422 | 0.024201 |
| 0.295427 | NA | 0.082535 | -0.00599 | -0.03583 | NA | 5 | -31.1279 | 72.49196 | 3.297807 | 0.022936 |
| 0.525857 | 0.032141 | 0.039502 | -0.05571 | NA | -0.03815 | 6 | -30.099 | 72.53001 | 3.335854 | 0.022504 |
| 0.337661 | 0.029385 | 0.027439 | NA | NA | -0.03328 | 5 | -31.3448 | 72.92581 | 3.731654 | 0.018463 |
| 0.330198 | 0.021546 | 0.027281 | NA | NA | NA | 4 | -32.409 | 72.97481 | 3.780655 | 0.018017 |
| 0.490773 | 0.022928 | 0.037614 | -0.04781 | NA | NA | 5 | -31.4833 | 73.20278 | 4.008618 | 0.016076 |
| 0.292269 | NA | 0.087386 | -0.00772 | -0.03567 | -0.01136 | 6 | -30.9757 | 74.28344 | 5.08928 | 0.009365 |
| 0.240242 | NA | 0.062859 | NA | NA | NA | 3 | -34.2276 | 74.54899 | 5.354831 | 0.008201 |
| 0.374233 | NA | 0.073778 | -0.04138 | NA | NA | 4 | -33.5388 | 75.23442 | 6.040259 | 0.005821 |
| 0.232607 | NA | 0.066818 | NA | NA | -0.01006 | 4 | -34.1103 | 76.37747 | 7.183313 | 0.003287 |
| 0.500544 | NA | NA | NA | -0.02528 | NA | 3 | -35.3702 | 76.83421 | 7.640052 | 0.002616 |
| 0.370497 | NA | 0.079001 | -0.04307 | NA | -0.01213 | 5 | -33.3682 | 76.97263 | 7.778478 | 0.002441 |
| 0.435601 | NA | NA | NA | NA | NA | 2 | -36.8921 | 77.83097 | 8.636811 | 0.001589 |
| 0.429687 | NA | NA | 0.020015 | -0.02931 | NA | 4 | -35.231 | 78.61886 | 9.424703 | 0.001072 |
| 0.498346 | NA | NA | NA | -0.02555 | 0.006238 | 4 | -35.3216 | 78.80008 | 9.605919 | 0.000979 |
| 0.483552 | NA | NA | -0.01182 | NA | NA | 3 | -36.8308 | 79.75525 | 10.5611 | 0.000607 |
| 0.433692 | NA | NA | NA | NA | 0.00411 | 3 | -36.8712 | 79.83615 | 10.64199 | 0.000583 |
| 0.427045 | NA | NA | 0.02013 | -0.02961 | 0.00634 | 5 | -35.1807 | 80.59767 | 11.40352 | 0.000398 |
| 0.48215 | NA | NA | -0.01196 | NA | 0.004251 | 4 | -36.8084 | 81.77357 | 12.57942 | 0.000221 |

## **Table S14 – Model selection for forbs under irrigation, colonisation, mean**

| (Intercept) | log(Nf) | log(Ng) | log(Nl + exp(-1)) | df | logLik | AICc | delta | weight |
| --- | --- | --- | --- | --- | --- | --- | --- | --- |
| **-0.2914** | **0.178925** | **NA** | **NA** | **3** | **-99.7923** | **205.797** | **0** | **0.297998** |
| -0.47799 | 0.16185 | NA | 0.085702 | 4 | -98.9079 | 206.173 | 0.375983 | 0.246928 |
| -0.66855 | 0.161774 | 0.10457 | NA | 4 | -99.4887 | 207.3346 | 1.537581 | 0.138144 |
| -0.76205 | 0.149468 | 0.081982 | 0.080362 | 5 | -98.7222 | 207.9848 | 2.187815 | 0.099801 |
| -0.03818 | NA | NA | 0.109461 | 3 | -101.204 | 208.6205 | 2.823445 | 0.072629 |
| 0.268377 | NA | NA | NA | 2 | -102.626 | 209.3582 | 3.561157 | 0.050225 |
| -0.61977 | NA | 0.150074 | 0.096359 | 4 | -100.559 | 209.4745 | 3.677483 | 0.047387 |
| -0.49151 | NA | 0.184445 | NA | 3 | -101.642 | 209.4957 | 3.698643 | 0.046888 |

## **Table S15 – Model selection for forbs under irrigation, colonisation, s.d.**

| (Intercept) | log(Nf) | log(Ng) | log(Nl + exp(-1)) | df | logLik | AICc | delta | weight |
| --- | --- | --- | --- | --- | --- | --- | --- | --- |
| **-0.11691** | **0.162892** | **NA** | **NA** | **3** | **-56.9149** | **120.0422** | **0** | **0.434437** |
| -0.22797 | 0.15273 | NA | 0.051008 | 4 | -56.2642 | 120.8855 | 0.843316 | 0.284972 |
| -0.08889 | 0.164166 | -0.00777 | NA | 4 | -56.9114 | 122.18 | 2.137795 | 0.14918 |
| -0.14994 | 0.156131 | -0.02252 | 0.052475 | 5 | -56.2351 | 123.0108 | 2.968676 | 0.098466 |
| 0.18706 | NA | NA | 0.073429 | 3 | -60.4345 | 127.0814 | 7.039232 | 0.012864 |
| 0.392703 | NA | NA | NA | 2 | -61.721 | 127.5473 | 7.505135 | 0.010191 |
| -0.00131 | NA | 0.048608 | 0.069185 | 4 | -60.2992 | 128.9555 | 8.913332 | 0.00504 |
| 0.090776 | NA | 0.073286 | NA | 3 | -61.41 | 129.0323 | 8.990138 | 0.00485 |

## **Table S16 – Model selection for grasses under control, persistence**

| (Intercept) | I((log(size))^2) | log(Nf) | log(Ng) | log(Nl + exp(-1)) | log(size) | df | logLik | AICc | delta | weight |
| --- | --- | --- | --- | --- | --- | --- | --- | --- | --- | --- |
| **6.253228** | **NA** | **-0.41792** | **-0.61073** | **-0.677673566** | **0.510313** | **5** | **-310.31** | **630.729** | **0** | **0.340088** |
| 4.046939 | NA | -0.46167 | NA | -0.707951238 | 0.498471 | 4 | -311.509 | 631.0906 | 0.361608 | 0.283837 |
| 6.331535 | 0.013507 | -0.42011 | -0.6272 | -0.678728611 | 0.480332 | 6 | -310.278 | 632.7097 | 1.980722 | 0.126323 |
| 4.046544 | -0.0003 | -0.4616 | NA | -0.70791079 | 0.499143 | 5 | -311.509 | 633.127 | 2.398071 | 0.102532 |
| 5.444339 | NA | NA | -0.7628 | -0.66108201 | 0.495845 | 4 | -312.834 | 633.7402 | 3.011175 | 0.075461 |
| 2.484742 | NA | NA | NA | -0.692600076 | 0.480358 | 3 | -314.72 | 635.4831 | 4.754124 | 0.031568 |
| 5.486988 | 0.007759 | NA | -0.77285 | -0.6616212 | 0.478715 | 5 | -312.823 | 635.7544 | 5.0254 | 0.027564 |
| 2.481078 | -0.00916 | NA | NA | -0.691524481 | 0.500519 | 4 | -314.703 | 637.4794 | 6.750429 | 0.011635 |
| 7.103238 | 0.147864 | -0.42566 | -0.76021 | -0.685308491 | NA | 5 | -316.652 | 643.4122 | 12.68318 | 0.000599 |
| 4.336559 | 0.137243 | -0.47378 | NA | -0.720727734 | NA | 4 | -318.523 | 645.1192 | 14.39025 | 0.000255 |
| 6.227156 | 0.141215 | NA | -0.90407 | -0.666390261 | NA | 4 | -319.324 | 646.7214 | 15.99239 | 0.000115 |
| 2.722921 | 0.128256 | NA | NA | -0.702014514 | NA | 3 | -322.002 | 650.0475 | 19.31852 | 2.17E-05 |
| 5.764781 | NA | -0.40987 | -1.05367 | NA | 0.496959 | 4 | -324.496 | 657.0653 | 26.33633 | 6.50E-07 |
| 5.814225 | 0.010513 | -0.41139 | -1.06463 | NA | 0.473854 | 5 | -324.475 | 659.0599 | 28.33088 | 2.40E-07 |
| 4.898968 | NA | NA | -1.17149 | NA | 0.483874 | 3 | -326.917 | 659.8777 | 29.14875 | 1.59E-07 |
| 4.924392 | 0.005742 | NA | -1.17782 | NA | 0.471314 | 4 | -326.91 | 661.8936 | 31.16459 | 5.81E-08 |
| 4.283448 | NA | -0.39364 | NA | -0.676029778 | NA | 3 | -328.981 | 664.0055 | 33.27651 | 2.02E-08 |
| 1.811524 | NA | -0.49143 | NA | NA | 0.480712 | 3 | -329.356 | 664.7549 | 34.02593 | 1.39E-08 |
| 5.757138 | NA | -0.36184 | -0.41222 | -0.65047091 | NA | 4 | -328.381 | 664.8349 | 34.10596 | 1.34E-08 |
| 1.80273 | -0.01337 | -0.48868 | NA | NA | 0.509759 | 4 | -329.32 | 666.7118 | 35.98286 | 5.22E-09 |
| 5.071255 | NA | NA | -0.55022 | -0.636688593 | NA | 3 | -330.447 | 666.9382 | 36.20917 | 4.67E-09 |
| 2.94959 | NA | NA | NA | -0.667340639 | NA | 2 | -331.537 | 667.0961 | 36.36712 | 4.31E-09 |
| 0.203131 | NA | NA | NA | NA | 0.466367 | 2 | -332.961 | 669.944 | 39.21499 | 1.04E-09 |
| 6.444964 | 0.142633 | -0.41343 | -1.17249 | NA | NA | 4 | -331.162 | 670.3974 | 39.66838 | 8.27E-10 |
| 0.204737 | -0.02041 | NA | NA | NA | 0.510324 | 3 | -332.871 | 671.7848 | 41.05579 | 4.13E-10 |
| 5.549179 | 0.136792 | NA | -1.28601 | NA | NA | 3 | -333.693 | 673.43 | 42.70107 | 1.82E-10 |
| 2.039414 | 0.127511 | -0.49755 | NA | NA | NA | 3 | -337.232 | 680.5074 | 49.77841 | 5.28E-12 |
| 0.411347 | 0.120239 | NA | NA | NA | NA | 2 | -341.07 | 686.1612 | 55.43217 | 3.12E-13 |
| 5.582846 | NA | -0.35498 | -0.91201 | NA | NA | 3 | -342.932 | 691.908 | 61.17899 | 1.76E-14 |
| 4.86341 | NA | NA | -1.02441 | NA | NA | 2 | -344.917 | 693.8548 | 63.12586 | 6.67E-15 |
| 2.174474 | NA | -0.43488 | NA | NA | NA | 2 | -346.97 | 697.9621 | 67.23309 | 8.55E-16 |
| 0.736632 | NA | NA | NA | NA | NA | 1 | -350.089 | 702.1861 | 71.45708 | 1.03E-16 |

## **Table S17 – Model selection for grasses under control, expansion, mean**

| (Intercept) | I((log(size))^2) | log(Nf) | log(Ng) | log(Nl + exp(-1)) | log(size) | df | logLik | AICc | delta | weight |
| --- | --- | --- | --- | --- | --- | --- | --- | --- | --- | --- |
| 2.700579908 | 0.070355 | -0.14237 | -0.39858 | NA | 0.399754 | 6 | -468.644 | 949.5156 | 0 | 0.361764 |
| **2.345211743** | **0.073569** | **NA** | **-0.42451** | **NA** | **0.389544** | **5** | **-470.014** | **950.1894** | **0.673807** | **0.258291** |
| 2.748943579 | 0.071338 | -0.14258 | -0.4212 | 0.01424 | 0.397234 | 7 | -468.585 | 951.4743 | 1.958666 | 0.135864 |
| 2.391464249 | 0.074523 | NA | -0.44641 | 0.013764 | 0.387095 | 6 | -469.959 | 952.1455 | 2.629812 | 0.097133 |
| 2.562872441 | NA | -0.15641 | -0.36863 | NA | 0.609053 | 5 | -471.587 | 953.3367 | 3.821074 | 0.053541 |
| 2.163768751 | NA | NA | -0.39574 | NA | 0.608336 | 4 | -473.221 | 954.5494 | 5.033722 | 0.029199 |
| 2.576564808 | NA | -0.15653 | -0.37518 | 0.004199 | 0.609172 | 6 | -471.582 | 955.392 | 5.876355 | 0.01916 |
| 1.217169906 | 0.062342 | -0.17268 | NA | NA | 0.414812 | 5 | -473.088 | 956.339 | 6.823315 | 0.011933 |
| 2.173907319 | NA | NA | -0.40071 | 0.003179 | 0.608426 | 5 | -473.218 | 956.5979 | 7.082271 | 0.010484 |
| 1.312798894 | 0.060932 | -0.16754 | NA | -0.03802 | 0.419256 | 6 | -472.576 | 957.3798 | 7.864198 | 0.007091 |
| 0.661392677 | 0.065653 | NA | NA | NA | 0.40346 | 4 | -475.08 | 958.2688 | 8.753125 | 0.004547 |
| 1.193693337 | NA | -0.18319 | NA | NA | 0.600863 | 4 | -475.369 | 958.8457 | 9.330017 | 0.003408 |
| 0.786184577 | 0.063975 | NA | NA | -0.0423 | 0.40878 | 5 | -474.451 | 959.0634 | 9.547777 | 0.003056 |
| 1.2993644 | NA | -0.17728 | NA | -0.04178 | 0.601122 | 5 | -474.756 | 959.6745 | 10.15884 | 0.002251 |
| 0.600857675 | NA | NA | NA | NA | 0.599298 | 3 | -477.59 | 961.2437 | 11.7281 | 0.001027 |
| 0.739806336 | NA | NA | NA | -0.04652 | 0.599643 | 4 | -476.836 | 961.7791 | 12.26347 | 0.000786 |
| 2.638973689 | 0.182877 | NA | -0.44978 | NA | NA | 4 | -478.381 | 964.8707 | 15.35509 | 0.000168 |
| 2.941072205 | 0.182587 | -0.11847 | -0.42875 | NA | NA | 5 | -477.472 | 965.1055 | 15.58988 | 0.000149 |
| 2.726485904 | 0.183404 | NA | -0.49262 | 0.027125 | NA | 5 | -478.177 | 966.5157 | 17.00009 | 7.36E-05 |
| 3.032580828 | 0.183126 | -0.11916 | -0.47256 | 0.027816 | NA | 6 | -477.255 | 966.7385 | 17.22286 | 6.58E-05 |
| 1.350572047 | 0.178504 | -0.15019 | NA | NA | NA | 4 | -482.386 | 972.8806 | 23.36501 | 3.05E-06 |
| 0.862271471 | 0.178619 | NA | NA | NA | NA | 3 | -483.828 | 973.7202 | 24.2046 | 2.01E-06 |
| 1.42809159 | 0.178372 | -0.14589 | NA | -0.03036 | NA | 5 | -482.075 | 974.3119 | 24.7963 | 1.49E-06 |
| 0.965526721 | 0.178466 | NA | NA | -0.03427 | NA | 4 | -483.432 | 974.9727 | 25.4571 | 1.07E-06 |
| 1.472597804 | NA | NA | NA | NA | NA | 2 | -569.349 | 1142.731 | 193.2152 | 4.00E-43 |
| 1.984258076 | NA | -0.1575 | NA | NA | NA | 3 | -568.345 | 1142.754 | 193.2382 | 3.96E-43 |
| 2.391893792 | NA | NA | -0.23083 | NA | NA | 3 | -568.44 | 1142.944 | 193.4283 | 3.60E-43 |
| 2.75534626 | NA | -0.14234 | -0.20598 | NA | NA | 4 | -567.626 | 1143.36 | 193.8445 | 2.92E-43 |
| 1.599417804 | NA | NA | NA | -0.04231 | NA | 3 | -568.967 | 1143.998 | 194.4828 | 2.12E-43 |
| 2.08126623 | NA | -0.15209 | NA | -0.03823 | NA | 4 | -568.032 | 1144.172 | 194.6562 | 1.95E-43 |
| 2.337781423 | NA | NA | -0.20452 | -0.01691 | NA | 4 | -568.39 | 1144.888 | 195.3726 | 1.36E-43 |
| 2.702998805 | NA | -0.1419 | -0.18115 | -0.01601 | NA | 5 | -567.582 | 1145.325 | 195.8098 | 1.09E-43 |

## **Table S18 – Model selection for grasses under control, expansion, s.d.**

| (Intercept) | I((log(size))^2) | log(Nf) | log(Ng) | log(Nl + exp(-1)) | log(size) | df | logLik | AICc | delta | weight |
| --- | --- | --- | --- | --- | --- | --- | --- | --- | --- | --- |
| 0.148751 | NA | 0.144297 | NA | NA | 0.040827 | 4 | -270.952 | 550.0114 | 0 | 0.18764 |
| **0.141062** | **-0.02042** | **0.140855** | **NA** | **NA** | **0.101766** | **5** | **-270.229** | **550.6202** | **0.608857** | **0.138393** |
| 0.202468 | NA | 0.146042 | NA | NA | NA | 3 | -272.592 | 551.2481 | 1.236707 | 0.101106 |
| 0.126647 | NA | 0.143061 | NA | 0.008739 | 0.040773 | 5 | -270.872 | 551.9069 | 1.895507 | 0.072731 |
| 0.173789 | 0.008079 | 0.146373 | NA | NA | NA | 4 | -271.909 | 551.9263 | 1.914865 | 0.072031 |
| 0.236321 | NA | 0.146009 | -0.02358 | NA | 0.041351 | 5 | -270.906 | 551.9749 | 1.963478 | 0.070301 |
| 0.122206 | -0.02014 | 0.139842 | NA | 0.007496 | 0.10089 | 6 | -270.171 | 552.5687 | 2.557294 | 0.052242 |
| 0.196944 | -0.02012 | 0.141997 | -0.01502 | NA | 0.101199 | 6 | -270.211 | 552.6491 | 2.63766 | 0.050184 |
| 0.179682 | NA | 0.14477 | NA | 0.00898 | NA | 4 | -272.509 | 553.1251 | 3.113748 | 0.039553 |
| 0.249389 | NA | 0.146965 | -0.01253 | NA | NA | 4 | -272.579 | 553.2658 | 3.254399 | 0.036867 |
| 0.283199 | NA | 0.145605 | -0.04599 | 0.014375 | 0.041759 | 6 | -270.732 | 553.6925 | 3.681138 | 0.029784 |
| 0.14995 | 0.008119 | 0.145052 | NA | 0.009338 | NA | 5 | -271.819 | 553.8002 | 3.788806 | 0.028223 |
| 0.257826 | 0.008294 | 0.148049 | -0.02265 | NA | NA | 5 | -271.868 | 553.8976 | 3.886225 | 0.026881 |
| 0.236532 | -0.01931 | 0.141829 | -0.03353 | 0.011656 | 0.099137 | 7 | -270.097 | 554.4985 | 4.487084 | 0.019905 |
| 0.291867 | NA | 0.146608 | -0.03269 | 0.01299 | NA | 5 | -272.438 | 555.0384 | 5.027002 | 0.015196 |
| 0.307318 | 0.008585 | 0.147674 | -0.04635 | 0.015044 | NA | 6 | -271.679 | 555.5855 | 5.574065 | 0.011559 |
| 0.615722 | NA | NA | NA | NA | 0.04206 | 3 | -275.019 | 556.1022 | 6.090798 | 0.008927 |
| 0.594404 | -0.02312 | NA | NA | NA | 0.111026 | 4 | -274.109 | 556.3252 | 6.313799 | 0.007986 |
| 0.676902 | NA | NA | NA | NA | NA | 2 | -276.722 | 557.4771 | 7.465684 | 0.004489 |
| 0.57819 | NA | NA | NA | 0.012566 | 0.041966 | 4 | -274.858 | 557.8228 | 7.811441 | 0.003777 |
| 0.561747 | -0.02268 | NA | NA | 0.011069 | 0.109634 | 5 | -273.983 | 558.1286 | 8.11724 | 0.003241 |
| 0.60889 | NA | NA | 0.00173 | NA | 0.04202 | 4 | -275.019 | 558.145 | 8.133614 | 0.003215 |
| 0.649683 | 0.007966 | NA | NA | NA | NA | 3 | -276.073 | 558.2109 | 8.199549 | 0.00311 |
| 0.551369 | -0.02332 | NA | 0.01085 | NA | 0.111381 | 5 | -274.099 | 558.3605 | 8.349123 | 0.002886 |
| 0.638351 | NA | NA | NA | 0.012861 | NA | 3 | -276.555 | 559.1745 | 9.163074 | 0.001921 |
| 0.624648 | NA | NA | 0.013121 | NA | NA | 3 | -276.709 | 559.4816 | 9.470219 | 0.001648 |
| 0.657763 | NA | NA | -0.02223 | 0.015323 | 0.042454 | 5 | -274.825 | 559.8126 | 9.801177 | 0.001396 |
| 0.609847 | 0.008025 | NA | NA | 0.013222 | NA | 4 | -275.896 | 559.899 | 9.887646 | 0.001337 |
| 0.592132 | -0.02248 | NA | -0.00845 | 0.01213 | 0.109223 | 6 | -273.979 | 560.1849 | 10.17348 | 0.001159 |
| 0.635364 | 0.007932 | NA | 0.003625 | NA | NA | 4 | -276.072 | 560.2521 | 10.24074 | 0.001121 |
| 0.669198 | NA | NA | -0.00854 | 0.013922 | NA | 4 | -276.55 | 561.2082 | 11.19685 | 0.000695 |
| 0.686662 | 0.00824 | NA | -0.02149 | 0.0159 | NA | 5 | -275.866 | 561.8939 | 11.8825 | 0.000493 |

## **Table S19 – Model selection for grasses under control, colonisation, mean**

| (Intercept) | log(Nf) | log(Ng) | log(Nl + exp(-1)) | df | logLik | AICc | delta | weight |
| --- | --- | --- | --- | --- | --- | --- | --- | --- |
| **-0.86403** | **NA** | **0.385529** | **NA** | **3** | **-139.431** | **285.0805** | **0** | **0.256266** |
| 0.676877 | NA | NA | NA | 2 | -140.596 | 285.3005 | 0.219966 | 0.229575 |
| 0.488775 | NA | NA | 0.063755 | 3 | -140.014 | 286.2456 | 1.165035 | 0.143122 |
| -0.74482 | NA | 0.329563 | 0.035412 | 4 | -139.273 | 286.9135 | 1.832994 | 0.102485 |
| 0.463509 | 0.064283 | NA | NA | 3 | -140.453 | 287.1232 | 2.042694 | 0.092284 |
| -0.93731 | 0.036691 | 0.373394 | NA | 4 | -139.385 | 287.1362 | 2.055661 | 0.091687 |
| 0.416295 | 0.025417 | NA | 0.059727 | 4 | -139.993 | 288.3537 | 3.273166 | 0.049881 |
| -0.79205 | 0.018863 | 0.327811 | 0.032573 | 5 | -139.262 | 289.0795 | 3.998934 | 0.0347 |

## **Table S20 – Model selection for grasses under control, colonisation, s.d.**

| (Intercept) | log(Nf) | log(Ng) | log(Nl + exp(-1)) | df | logLik | AICc | delta | weight |
| --- | --- | --- | --- | --- | --- | --- | --- | --- |
| -0.21245 | **NA** | **0.222441** | **NA** | **3** | **-73.7291** | **153.6764** | **0** | **0.289056** |
| 0.676614 | NA | NA | NA | 2 | -74.9566 | 154.0213 | 0.344859 | 0.243275 |
| 0.60469 | NA | NA | 0.024378 | 3 | -74.6879 | 155.594 | 1.91763 | 0.110809 |
| -0.19201 | NA | 0.212844 | 0.006073 | 4 | -73.7144 | 155.7958 | 2.119418 | 0.100174 |
| -0.20311 | -0.00468 | 0.223989 | NA | 4 | -73.7267 | 155.8204 | 2.143997 | 0.098951 |
| 0.637205 | 0.011873 | NA | NA | 3 | -74.9411 | 156.1004 | 2.42396 | 0.086025 |
| 0.617377 | -0.00445 | NA | 0.025083 | 4 | -74.686 | 157.7389 | 4.062502 | 0.037916 |
| -0.17017 | -0.00872 | 0.213654 | 0.007385 | 5 | -73.7068 | 157.9691 | 4.292715 | 0.033793 |

## **Table S21 – Model selection for legumes under control, persistence**

| (Intercept) | I((log(size))^2) | log(Nf) | log(Ng) | log(Nl + exp(-1)) | log(size) | df | logLik | AICc | delta | weight |
| --- | --- | --- | --- | --- | --- | --- | --- | --- | --- | --- |
| **4.469706** | **-0.0812** | **NA** | **NA** | **-1.00824** | **0.336324** | **4** | **-147.4** | **302.942** | **0** | **0.148848** |
| 4.590801 | NA | NA | NA | -1.0037 | NA | 2 | -149.476 | 302.9951 | 0.053129 | 0.144946 |
| 4.696086 | NA | NA | NA | -1.08468 | 0.147559 | 3 | -148.506 | 303.0963 | 0.154277 | 0.137797 |
| 2.350687 | -0.08791 | NA | 0.547846 | -1.03201 | 0.353337 | 5 | -146.937 | 304.0891 | 1.147107 | 0.083879 |
| 2.944322 | NA | NA | 0.430459 | -1.02813 | NA | 3 | -149.182 | 304.4498 | 1.507795 | 0.070037 |
| 3.022462 | NA | NA | 0.435722 | -1.10769 | 0.148113 | 4 | -148.208 | 304.558 | 1.616038 | 0.066347 |
| 4.788265 | NA | -0.05338 | NA | -1.00992 | NA | 3 | -149.454 | 304.9923 | 2.050285 | 0.053398 |
| 4.586503 | -0.08066 | -0.03132 | NA | -1.01236 | 0.335228 | 5 | -147.392 | 304.9987 | 2.056681 | 0.053228 |
| 4.61452 | 0.004715 | NA | NA | -1.015 | NA | 3 | -149.467 | 305.0193 | 2.077276 | 0.052683 |
| 4.929037 | NA | -0.06273 | NA | -1.09248 | 0.148205 | 4 | -148.475 | 305.0914 | 2.149397 | 0.050817 |
| 2.534318 | -0.08694 | -0.08488 | 0.58428 | -1.04684 | 0.351515 | 6 | -146.884 | 306.069 | 3.127037 | 0.031168 |
| 3.145485 | NA | -0.09904 | 0.475887 | -1.04452 | NA | 4 | -149.108 | 306.3576 | 3.415593 | 0.026981 |
| 3.248649 | NA | -0.10826 | 0.483946 | -1.12613 | 0.149125 | 5 | -148.12 | 306.4535 | 3.5115 | 0.025718 |
| 2.970637 | 0.002975 | NA | 0.427385 | -1.03498 | NA | 4 | -149.179 | 306.4997 | 3.55767 | 0.025131 |
| 4.822812 | 0.005182 | -0.05564 | NA | -1.02264 | NA | 4 | -149.442 | 307.0273 | 4.085252 | 0.019304 |
| 3.179817 | 0.003572 | -0.10021 | 0.472659 | -1.05296 | NA | 5 | -149.102 | 308.419 | 5.476963 | 0.009626 |
| 1.169823 | -0.13297 | NA | NA | NA | 0.316395 | 3 | -156.985 | 320.0558 | 17.1138 | 2.86E-05 |
| 1.278027 | -0.05219 | NA | NA | NA | NA | 2 | -158.999 | 322.0403 | 19.09831 | 1.06E-05 |
| 1.015753 | -0.13345 | 0.047008 | NA | NA | 0.317812 | 4 | -156.968 | 322.0788 | 19.13677 | 1.04E-05 |
| 0.85043 | -0.13418 | NA | 0.079878 | NA | 0.318573 | 4 | -156.972 | 322.0862 | 19.14423 | 1.04E-05 |
| 1.107958 | NA | NA | NA | NA | NA | 1 | -160.276 | 322.5665 | 19.6245 | 8.15E-06 |
| 1.190618 | -0.05224 | 0.026687 | NA | NA | NA | 3 | -158.993 | 324.0717 | 21.12966 | 3.84E-06 |
| 1.267721 | -0.05221 | NA | 0.002581 | NA | NA | 3 | -158.999 | 324.083 | 21.14099 | 3.82E-06 |
| 0.75661 | -0.13443 | 0.04207 | 0.06885 | NA | 0.319544 | 5 | -156.958 | 324.1313 | 21.18925 | 3.73E-06 |
| 1.450117 | NA | NA | -0.08517 | NA | NA | 2 | -160.26 | 324.5633 | 21.6213 | 3.00E-06 |
| 1.048877 | NA | 0.018015 | NA | NA | NA | 2 | -160.274 | 324.5897 | 21.64769 | 2.96E-06 |
| 1.108159 | NA | NA | NA | NA | -0.00017 | 2 | -160.276 | 324.5948 | 21.65281 | 2.96E-06 |
| 1.209252 | -0.0522 | 0.027053 | -0.00497 | NA | NA | 4 | -158.993 | 326.1288 | 23.1868 | 1.37E-06 |
| 1.395327 | NA | 0.024294 | -0.09136 | NA | NA | 3 | -160.256 | 326.5968 | 23.65483 | 1.09E-06 |
| 1.44998 | NA | NA | -0.08537 | NA | 0.000841 | 3 | -160.26 | 326.6059 | 23.66394 | 1.08E-06 |
| 1.049198 | NA | 0.018029 | NA | NA | -0.00032 | 3 | -160.274 | 326.6324 | 23.69038 | 1.07E-06 |
| 1.395255 | NA | 0.024272 | -0.09153 | NA | 0.000706 | 4 | -160.256 | 328.654 | 25.71203 | 3.89E-07 |

## **Table S22 – Model selection for legumes under control, expansion, mean**

| (Intercept) | I((log(size))^2) | log(Nf) | log(Ng) | log(Nl + exp(-1)) | log(size) | df | logLik | AICc | delta | weight |
| --- | --- | --- | --- | --- | --- | --- | --- | --- | --- | --- |
| 0.556798 | 0.109686 | NA | NA | NA | 0.320588 | 4 | -276.436 | 561.0624 | 0 | 0.307612 |
| 0.868072 | 0.104611 | -0.09782 | NA | NA | 0.341483 | 5 | -276.014 | 562.3144 | 1.251978 | 0.16449 |
| **0.731415** | **0.113056** | **NA** | **NA** | **-0.05933** | **0.32565** | **5** | **-276.131** | **562.5483** | **1.48589** | **0.146335** |
| 0.955207 | 0.110855 | NA | -0.09969 | NA | 0.319255 | 5 | -276.309 | 562.9051 | 1.842663 | 0.122426 |
| 1.04939 | 0.107975 | -0.099 | NA | -0.06032 | 0.346884 | 6 | -275.697 | 563.7974 | 2.735044 | 0.07836 |
| 1.155384 | 0.105797 | -0.09222 | -0.07635 | NA | 0.339266 | 6 | -275.94 | 564.2844 | 3.222012 | 0.061426 |
| 0.952357 | 0.113384 | NA | -0.06019 | -0.05268 | 0.324277 | 6 | -276.088 | 564.58 | 3.517567 | 0.052988 |
| 1.161685 | 0.108281 | -0.09653 | -0.03275 | -0.05668 | 0.345606 | 7 | -275.684 | 565.9099 | 4.847504 | 0.027251 |
| 0.682666 | 0.196126 | NA | NA | NA | NA | 3 | -280.891 | 567.8963 | 6.833918 | 0.010093 |
| 0.81717 | 0.199732 | NA | NA | -0.04519 | NA | 4 | -280.721 | 569.6321 | 8.569738 | 0.004237 |
| 1.139581 | 0.197056 | NA | -0.11448 | NA | NA | 4 | -280.731 | 569.6515 | 8.589115 | 0.004197 |
| 0.773251 | 0.196284 | -0.02774 | NA | NA | NA | 4 | -280.857 | 569.905 | 8.842638 | 0.003697 |
| 0.995021 | NA | -0.15532 | NA | NA | 0.65116 | 4 | -281.022 | 570.2347 | 9.172296 | 0.003135 |
| 0.496311 | NA | NA | NA | NA | 0.641565 | 3 | -282.065 | 570.2443 | 9.181872 | 0.00312 |
| 1.139616 | 0.199677 | NA | -0.08798 | -0.03555 | NA | 5 | -280.634 | 571.5541 | 10.49174 | 0.001621 |
| 0.908009 | 0.199892 | -0.0278 | NA | -0.04521 | NA | 5 | -280.687 | 571.6603 | 10.59793 | 0.001537 |
| 1.186313 | 0.197132 | -0.02035 | -0.10954 | NA | NA | 5 | -280.712 | 571.712 | 10.64964 | 0.001498 |
| 1.082326 | NA | -0.15675 | NA | -0.0284 | 0.658393 | 5 | -280.954 | 572.1951 | 11.13269 | 0.001176 |
| 0.567202 | NA | NA | NA | -0.02435 | 0.647688 | 4 | -282.016 | 572.2218 | 11.15939 | 0.001161 |
| 0.699925 | NA | NA | -0.05103 | NA | 0.642634 | 4 | -282.034 | 572.2576 | 11.19516 | 0.00114 |
| 1.054374 | NA | -0.1543 | -0.01569 | NA | 0.651426 | 5 | -281.019 | 572.3253 | 11.26293 | 0.001102 |
| 1.190708 | 0.199809 | -0.02225 | -0.08208 | -0.03621 | NA | 6 | -280.612 | 573.6278 | 12.56542 | 0.000575 |
| 0.696569 | NA | NA | -0.03532 | -0.02038 | 0.647431 | 5 | -282.002 | 574.2906 | 13.22823 | 0.000413 |
| 1.056402 | NA | -0.15728 | 0.007555 | -0.02927 | 0.658484 | 6 | -280.953 | 574.3106 | 13.24819 | 0.000409 |
| 0.237067 | NA | NA | NA | 0.312548 | NA | 3 | -337.501 | 681.1158 | 120.0534 | 2.62E-27 |
| 0.635293 | NA | NA | -0.1086 | 0.324325 | NA | 4 | -337.423 | 683.0357 | 121.9733 | 1.00E-27 |
| 0.16896 | NA | 0.020947 | NA | 0.312346 | NA | 4 | -337.49 | 683.1696 | 122.1072 | 9.39E-28 |
| 0.569671 | NA | 0.028768 | -0.11622 | 0.324873 | NA | 5 | -337.401 | 685.0897 | 124.0273 | 3.60E-28 |
| 1.238067 | NA | NA | NA | NA | NA | 2 | -342.975 | 690.0063 | 128.9439 | 3.08E-29 |
| 0.565982 | NA | NA | 0.167432 | NA | NA | 3 | -342.78 | 691.6728 | 130.6104 | 1.34E-29 |
| 1.147625 | NA | 0.027555 | NA | NA | NA | 3 | -342.956 | 692.0257 | 130.9633 | 1.12E-29 |
| 0.528996 | NA | 0.016185 | 0.163411 | NA | NA | 4 | -342.773 | 693.7367 | 132.6743 | 4.77E-30 |

## **Table S23 – Model selection for legumes under control, expansion, s.d.**

| (Intercept) | I((log(size))^2) | log(Nf) | log(Ng) | log(Nl + exp(-1)) | log(size) | df | logLik | AICc | delta | weight |
| --- | --- | --- | --- | --- | --- | --- | --- | --- | --- | --- |
| **0.598447** | **NA** | **0.087334** | **NA** | **-0.07379** | **0.05569** | **5** | **-154.569** | **319.4251** | **0** | **0.077678** |
| 0.885448 | NA | NA | NA | -0.07605 | 0.061654 | 4 | -155.637 | 319.4642 | 0.039061 | 0.076175 |
| 0.847533 | -0.0261 | NA | NA | -0.06798 | 0.13601 | 5 | -154.643 | 319.5732 | 0.14808 | 0.072134 |
| 0.380286 | NA | 0.101413 | NA | NA | NA | 3 | -156.905 | 319.9245 | 0.499394 | 0.060514 |
| 0.647462 | -0.02996 | NA | NA | NA | 0.13021 | 4 | -155.879 | 319.948 | 0.522928 | 0.059806 |
| 0.605228 | -0.02223 | 0.075444 | NA | -0.06722 | 0.119829 | 6 | -153.862 | 320.1276 | 0.70253 | 0.054669 |
| 0.371638 | NA | 0.09105 | NA | NA | 0.036899 | 4 | -156.049 | 320.2878 | 0.862724 | 0.050461 |
| 0.403167 | -0.02598 | 0.076768 | NA | NA | 0.113811 | 5 | -155.079 | 320.445 | 1.019925 | 0.046647 |
| 0.663986 | NA | NA | NA | NA | 0.042524 | 3 | -157.195 | 320.5028 | 1.077707 | 0.045319 |
| 0.713151 | NA | NA | NA | NA | NA | 2 | -158.339 | 320.7338 | 1.308709 | 0.040375 |
| 0.521189 | NA | 0.102365 | NA | -0.04497 | NA | 4 | -156.28 | 320.7504 | 1.325263 | 0.040042 |
| 0.801259 | NA | NA | 0.022983 | -0.07863 | 0.061822 | 5 | -155.618 | 321.5227 | 2.097562 | 0.027216 |
| 0.601322 | NA | 0.087393 | -0.00084 | -0.0737 | 0.05568 | 6 | -154.569 | 321.5418 | 2.116715 | 0.026956 |
| 0.854022 | NA | NA | NA | -0.04399 | NA | 3 | -157.748 | 321.6101 | 2.184985 | 0.026052 |
| 0.742017 | -0.02626 | NA | 0.028743 | -0.07115 | 0.136665 | 6 | -154.613 | 321.6299 | 2.204766 | 0.025795 |
| 0.371565 | 0.004572 | 0.100125 | NA | NA | NA | 4 | -156.761 | 321.7121 | 2.287007 | 0.024756 |
| 0.556389 | 0.009521 | 0.100043 | NA | -0.062 | NA | 5 | -155.738 | 321.764 | 2.338938 | 0.024121 |
| 0.565642 | NA | 0.10482 | -0.04896 | NA | NA | 4 | -156.813 | 321.816 | 2.390891 | 0.023503 |
| 0.745867 | -0.02968 | NA | -0.02462 | NA | 0.129881 | 5 | -155.855 | 321.9971 | 2.572006 | 0.021468 |
| 0.596213 | NA | 0.0949 | -0.05938 | NA | 0.037905 | 5 | -155.912 | 322.111 | 2.685889 | 0.02028 |
| 0.579638 | -0.0223 | 0.07488 | 0.007464 | -0.06805 | 0.12012 | 7 | -153.86 | 322.2608 | 2.835754 | 0.018816 |
| 0.572073 | -0.02528 | 0.080061 | -0.04489 | NA | 0.112507 | 6 | -155.001 | 322.4056 | 2.98049 | 0.017502 |
| 0.698585 | 0.005144 | NA | NA | NA | NA | 3 | -158.158 | 322.4292 | 3.004123 | 0.017297 |
| 0.814205 | NA | NA | -0.03765 | NA | 0.043312 | 4 | -157.139 | 322.4692 | 3.044093 | 0.016954 |
| 0.883349 | 0.010097 | NA | NA | -0.06207 | NA | 4 | -157.146 | 322.4832 | 3.0581 | 0.016836 |
| 0.805178 | NA | NA | -0.02293 | NA | NA | 3 | -158.318 | 322.7502 | 3.325083 | 0.014732 |
| 0.560165 | NA | 0.103125 | -0.0113 | -0.04375 | NA | 5 | -156.275 | 322.838 | 3.412863 | 0.014099 |
| 0.582329 | 0.005005 | 0.103892 | -0.05589 | NA | NA | 5 | -156.641 | 323.5696 | 4.144472 | 0.00978 |
| 0.795408 | NA | NA | 0.015985 | -0.04572 | NA | 4 | -157.739 | 323.6687 | 4.24363 | 0.009307 |
| 0.589725 | 0.009511 | 0.100697 | -0.00968 | -0.06094 | NA | 6 | -155.735 | 323.8742 | 4.449068 | 0.008398 |
| 0.820875 | 0.005393 | NA | -0.03064 | NA | NA | 4 | -158.122 | 324.4338 | 5.008756 | 0.006348 |
| 0.820937 | 0.010108 | NA | 0.017029 | -0.06394 | NA | 5 | -157.136 | 324.5592 | 5.134064 | 0.005963 |

## **Table S24 – Model selection for legumes under control, colonisation, mean**

| (Intercept) | log(Nf) | log(Ng) | log(Nl + exp(-1)) | df | logLik | AICc | delta | weight |
| --- | --- | --- | --- | --- | --- | --- | --- | --- |
| **0.288878** | **NA** | **NA** | **NA** | **2** | **-26.1461** | **56.67934** | **0** | **0.302181** |
| -0.27656 | 0.181088 | NA | NA | 3 | -25.4004 | 57.60082 | 0.921482 | 0.19062 |
| -1.18743 | NA | 0.367064 | NA | 3 | -25.4031 | 57.6062 | 0.926861 | 0.190108 |
| 0.224877 | NA | NA | 0.026346 | 3 | -25.9979 | 58.79574 | 2.1164 | 0.104881 |
| -1.28303 | 0.139568 | 0.282479 | NA | 4 | -24.9856 | 59.35051 | 2.671168 | 0.079475 |
| -1.53865 | NA | 0.468912 | -0.02405 | 4 | -25.333 | 60.04524 | 3.365902 | 0.056153 |
| -0.27031 | 0.173574 | NA | 0.007085 | 4 | -25.3906 | 60.16045 | 3.481108 | 0.05301 |
| -1.84008 | 0.157509 | 0.429581 | -0.0373 | 5 | -24.8192 | 61.78125 | 5.101907 | 0.023572 |

## **Table S25 – Model selection for legumes under control, colonisation, s.d.**

| (Intercept) | log(Nf) | log(Ng) | log(Nl + exp(-1)) | df | logLik | AICc | delta | weight |
| --- | --- | --- | --- | --- | --- | --- | --- | --- |
| **0.407827** | **NA** | **NA** | **NA** | **2** | **-10.1296** | **24.64636** | **0** | **0.300418** |
| 0.046916 | 0.115586 | NA | NA | 3 | -9.34943 | 25.49886 | 0.852498 | 0.196159 |
| -0.50133 | NA | 0.226049 | NA | 3 | -9.40715 | 25.61429 | 0.967929 | 0.185158 |
| 0.363751 | NA | NA | 0.018144 | 3 | -9.94907 | 26.69814 | 2.051775 | 0.107693 |
| -0.56326 | 0.090415 | 0.171253 | NA | 4 | -8.95777 | 27.29485 | 2.648483 | 0.079913 |
| 0.052236 | 0.109193 | NA | 0.006027 | 4 | -9.33111 | 28.04154 | 3.395175 | 0.055014 |
| -0.66616 | NA | 0.273847 | -0.01129 | 4 | -9.3676 | 28.11451 | 3.468147 | 0.053043 |
| -0.85731 | 0.099885 | 0.248905 | -0.01969 | 5 | -8.8389 | 29.82065 | 5.174285 | 0.022602 |

## **Table S26 – Model selection for forbs under control, persistence**

| (Intercept) | I((log(size))^2) | log(Nf) | log(Ng) | log(Nl + exp(-1)) | log(size) | df | logLik | AICc | delta | weight |
| --- | --- | --- | --- | --- | --- | --- | --- | --- | --- | --- |
| 4.650234 | NA | -0.59584 | -0.5543 | NA | 0.470098 | 4 | -525.104 | 1058.255 | 0 | 0.433913 |
| **4.460571** | **NA** | **-0.59059** | **-0.48189** | **-0.03808** | **0.469091** | **5** | **-524.966** | **1060.003** | **1.748109** | **0.181053** |
| 4.722483 | 0.013908 | -0.61241 | -0.56113 | NA | 0.463647 | 5 | -525.062 | 1060.194 | 1.939047 | 0.164567 |
| 2.911628 | NA | -0.63037 | NA | -0.11546 | 0.477942 | 4 | -526.814 | 1061.676 | 3.420861 | 0.078446 |
| 4.532739 | 0.013672 | -0.60692 | -0.48899 | -0.0379 | 0.462763 | 6 | -524.926 | 1061.949 | 3.694643 | 0.06841 |
| 2.729579 | NA | -0.68079 | NA | NA | 0.487937 | 3 | -528.706 | 1063.44 | 5.185409 | 0.032464 |
| 2.928845 | 0.004815 | -0.63633 | NA | -0.11577 | 0.475786 | 5 | -526.809 | 1063.689 | 5.433993 | 0.02867 |
| 2.730654 | 0.000312 | -0.68118 | NA | NA | 0.487799 | 4 | -528.706 | 1065.459 | 7.204069 | 0.011832 |
| 3.44851 | NA | NA | -0.74213 | NA | 0.396921 | 3 | -533.665 | 1073.358 | 15.10329 | 0.000228 |
| 3.294233 | -0.05516 | NA | -0.69352 | NA | 0.426682 | 4 | -532.857 | 1073.76 | 15.50504 | 0.000186 |
| 3.191828 | NA | NA | -0.63595 | -0.05458 | 0.396056 | 4 | -533.37 | 1074.787 | 16.53201 | 0.000112 |
| 3.041124 | -0.0548 | NA | -0.58892 | -0.05378 | 0.425701 | 5 | -532.571 | 1075.212 | 16.95741 | 9.02E-05 |
| 0.996265 | -0.06954 | NA | NA | -0.14782 | 0.440269 | 4 | -535.368 | 1078.782 | 20.52716 | 1.51E-05 |
| 0.976146 | NA | NA | NA | -0.15769 | 0.404442 | 3 | -536.694 | 1079.417 | 21.16205 | 1.10E-05 |
| 0.559717 | -0.08136 | NA | NA | NA | 0.454257 | 3 | -538.666 | 1083.359 | 25.10441 | 1.53E-06 |
| 0.500804 | NA | NA | NA | NA | 0.414614 | 2 | -540.503 | 1085.021 | 26.7657 | 6.69E-07 |
| 5.024493 | 0.069916 | -0.52309 | -0.68128 | NA | NA | 4 | -539.909 | 1087.865 | 29.61065 | 1.61E-07 |
| 4.620948 | NA | -0.41321 | -0.65345 | NA | NA | 3 | -541.311 | 1088.651 | 30.39599 | 1.09E-07 |
| 4.807918 | 0.069466 | -0.51664 | -0.59875 | -0.04377 | NA | 5 | -539.724 | 1089.517 | 31.26242 | 7.06E-08 |
| 4.396644 | NA | -0.40727 | -0.56721 | -0.04571 | NA | 4 | -541.108 | 1090.262 | 32.00713 | 4.87E-08 |
| 2.841024 | 0.061904 | -0.55083 | NA | -0.13915 | NA | 4 | -542.632 | 1093.31 | 35.05557 | 1.06E-08 |
| 2.567309 | NA | -0.45163 | NA | -0.13653 | NA | 3 | -543.746 | 1093.52 | 35.26493 | 9.54E-09 |
| 3.777541 | NA | NA | -0.78745 | NA | NA | 2 | -545.917 | 1095.847 | 37.59249 | 2.98E-09 |
| 2.604686 | 0.05922 | -0.6044 | NA | NA | NA | 3 | -545.451 | 1096.93 | 38.67479 | 1.73E-09 |
| 2.347142 | NA | -0.50853 | NA | NA | NA | 2 | -546.475 | 1096.964 | 38.70908 | 1.71E-09 |
| 3.507683 | NA | NA | -0.67541 | -0.05801 | NA | 3 | -545.579 | 1097.187 | 38.93174 | 1.53E-09 |
| 3.796617 | 0.007671 | NA | -0.79427 | NA | NA | 3 | -545.896 | 1097.82 | 39.56481 | 1.11E-09 |
| 3.526945 | 0.007938 | NA | -0.68224 | -0.05814 | NA | 4 | -545.557 | 1099.161 | 40.90576 | 5.69E-10 |
| 1.158479 | NA | NA | NA | -0.16829 | NA | 2 | -549.422 | 1102.858 | 44.6029 | 8.95E-11 |
| 1.161762 | -0.00542 | NA | NA | -0.1675 | NA | 3 | -549.411 | 1104.85 | 46.59559 | 3.31E-11 |
| 0.653384 | NA | NA | NA | NA | NA | 1 | -553.837 | 1109.678 | 51.42303 | 2.96E-12 |
| 0.670144 | -0.01606 | NA | NA | NA | NA | 2 | -553.742 | 1111.498 | 53.24326 | 1.19E-12 |

## **Table S27 – Model selection for forbs under control, expansion, mean**

| (Intercept) | I((log(size))^2) | log(Nf) | log(Ng) | log(Nl + exp(-1)) | log(size) | df | logLik | AICc | delta | weight |
| --- | --- | --- | --- | --- | --- | --- | --- | --- | --- | --- |
| **-0.416982209** | **0.101694** | **NA** | **0.236052** | **-0.05655** | **0.164809** | **6** | **-532.917** | **1077.985** | **0** | **0.413991** |
| -0.605014525 | 0.094735 | 0.071291 | 0.228364 | -0.05832 | 0.160956 | 7 | -532.109 | 1078.418 | 0.433405 | 0.333333 |
| -0.097964745 | 0.101192 | NA | 0.11294 | NA | 0.167557 | 5 | -535.67 | 1081.447 | 3.461912 | 0.073324 |
| -0.25628126 | 0.094989 | 0.063404 | 0.102663 | NA | 0.164207 | 6 | -535.035 | 1082.219 | 4.234458 | 0.04983 |
| 0.341808137 | 0.106746 | NA | NA | NA | 0.160451 | 4 | -537.185 | 1082.442 | 4.456902 | 0.044585 |
| 0.104954636 | 0.098735 | 0.075718 | NA | NA | 0.157222 | 5 | -536.266 | 1082.638 | 4.653755 | 0.040406 |
| 0.132471027 | 0.100009 | 0.082471 | NA | -0.01715 | 0.153751 | 6 | -535.856 | 1083.861 | 5.876428 | 0.021925 |
| 0.380019072 | 0.108308 | NA | NA | -0.01347 | 0.15795 | 5 | -536.928 | 1083.964 | 5.979174 | 0.020827 |
| -0.530874612 | 0.141802 | 0.086731 | 0.208513 | -0.06126 | NA | 6 | -539.34 | 1090.83 | 12.84513 | 0.000673 |
| -0.298755617 | 0.151689 | NA | 0.217333 | -0.05917 | NA | 5 | -540.512 | 1091.13 | 13.14566 | 0.000579 |
| 0.104541718 | 0.14434 | 0.087487 | NA | NA | NA | 4 | -543.152 | 1094.375 | 16.39066 | 0.000114 |
| 0.379079065 | 0.154711 | NA | NA | NA | NA | 3 | -544.353 | 1094.749 | 16.76473 | 9.47E-05 |
| 0.142090511 | 0.144704 | 0.096345 | NA | -0.0234 | NA | 5 | -542.4 | 1094.908 | 16.92318 | 8.75E-05 |
| 0.037386158 | 0.152037 | NA | 0.088082 | NA | NA | 4 | -543.447 | 1094.966 | 16.98085 | 8.50E-05 |
| -0.16265271 | 0.143068 | 0.078766 | 0.075932 | NA | NA | 5 | -542.488 | 1095.083 | 17.09833 | 8.02E-05 |
| 0.432928102 | 0.155877 | NA | NA | -0.01928 | NA | 4 | -543.837 | 1095.745 | 17.76046 | 5.76E-05 |
| -0.989443626 | NA | 0.149084 | 0.268905 | -0.05896 | 0.292279 | 6 | -543.889 | 1099.927 | 21.94261 | 7.12E-06 |
| -0.637919204 | NA | 0.141321 | 0.141932 | NA | 0.295922 | 5 | -546.758 | 1103.622 | 25.63729 | 1.12E-06 |
| -0.626159489 | NA | NA | 0.293153 | -0.05503 | 0.32297 | 5 | -547.557 | 1105.222 | 27.23693 | 5.04E-07 |
| -0.151982789 | NA | 0.162919 | NA | NA | 0.293408 | 4 | -549.046 | 1106.163 | 28.17784 | 3.15E-07 |
| -0.137816413 | NA | 0.167531 | NA | -0.01004 | 0.292404 | 5 | -548.911 | 1107.928 | 29.94361 | 1.30E-07 |
| -0.314649482 | NA | NA | 0.173051 | NA | 0.324884 | 4 | -550.035 | 1108.14 | 30.15553 | 1.17E-07 |
| 0.360697391 | NA | NA | NA | NA | 0.327297 | 3 | -553.506 | 1113.055 | 35.07051 | 1.00E-08 |
| 0.362292265 | NA | NA | NA | -0.00056 | 0.327294 | 4 | -553.506 | 1115.083 | 37.09821 | 3.64E-09 |
| -1.346274854 | NA | 0.314258 | 0.269791 | -0.06889 | NA | 5 | -580.395 | 1170.897 | 92.91194 | 2.76E-21 |
| -0.939900112 | NA | 0.307579 | 0.121138 | NA | NA | 4 | -583.842 | 1175.755 | 97.76999 | 2.44E-22 |
| -0.522448905 | NA | 0.324829 | NA | NA | NA | 3 | -585.308 | 1176.659 | 98.6742 | 1.55E-22 |
| -0.491994744 | NA | 0.332837 | NA | -0.01981 | NA | 4 | -584.844 | 1177.759 | 99.77394 | 8.94E-23 |
| -0.569063433 | NA | NA | 0.327855 | -0.062 | NA | 4 | -596.266 | 1200.603 | 122.6184 | 9.79E-28 |
| -0.217462392 | NA | NA | 0.192675 | NA | NA | 3 | -598.915 | 1203.873 | 125.8881 | 1.91E-28 |
| 0.535904101 | NA | NA | NA | NA | NA | 2 | -602.539 | 1209.1 | 131.1152 | 1.40E-29 |
| 0.539005176 | NA | NA | NA | -0.00109 | NA | 3 | -602.538 | 1211.119 | 133.1339 | 5.10E-30 |

## **Table S28 – Model selection for forbs under control, expansion, s.d.**

| (Intercept) | I((log(size))^2) | log(Nf) | log(Ng) | log(Nl + exp(-1)) | log(size) | df | logLik | AICc | delta | weight |
| --- | --- | --- | --- | --- | --- | --- | --- | --- | --- | --- |
| **-0.20337** | **0.029425** | **0.085946** | **0.124428** | **-0.04128** | **0.04593** | **7** | **-198.21** | **410.6213** | **0** | **0.562968** |
| -0.18221 | 0.042855 | 0.090352 | 0.118764 | -0.04212 | NA | 6 | -200.14 | 412.4309 | 1.809582 | 0.227792 |
| 0.198464 | 0.032298 | 0.092037 | NA | -0.01885 | 0.042004 | 6 | -201.823 | 415.7963 | 5.175049 | 0.042339 |
| -0.32277 | NA | 0.110108 | 0.13702 | -0.04148 | 0.086719 | 6 | -201.953 | 416.0569 | 5.435597 | 0.037167 |
| 0.023317 | 0.037814 | NA | 0.133696 | -0.03914 | 0.050576 | 6 | -202.006 | 416.1617 | 5.540439 | 0.035269 |
| 0.201092 | 0.044509 | 0.095828 | NA | -0.02056 | NA | 5 | -203.424 | 416.955 | 6.333724 | 0.023721 |
| 0.168222 | 0.030898 | 0.084615 | NA | NA | 0.045819 | 5 | -203.429 | 416.9657 | 6.344371 | 0.023595 |
| 0.043477 | 0.029604 | 0.080363 | 0.035452 | NA | 0.048231 | 6 | -202.955 | 418.06 | 7.438761 | 0.013651 |
| 0.168102 | 0.044188 | 0.088045 | NA | NA | NA | 4 | -205.338 | 418.7474 | 8.126091 | 0.009681 |
| 0.059598 | 0.053156 | NA | 0.127952 | -0.03995 | NA | 5 | -204.325 | 418.7576 | 8.136298 | 0.009632 |
| 0.070978 | 0.043726 | 0.084875 | 0.027601 | NA | NA | 5 | -205.049 | 420.2058 | 9.584512 | 0.004669 |
| 0.432909 | 0.03985 | NA | NA | NA | 0.049427 | 4 | -207.126 | 422.3227 | 11.70142 | 0.00162 |
| 0.474727 | 0.04156 | NA | NA | -0.01475 | 0.046691 | 5 | -206.139 | 422.3857 | 11.76441 | 0.00157 |
| 0.24414 | 0.037466 | NA | 0.048478 | NA | 0.052478 | 5 | -206.232 | 422.5716 | 11.95031 | 0.001431 |
| 0.111174 | NA | 0.119508 | NA | -0.01655 | 0.086782 | 5 | -206.314 | 422.7355 | 12.1142 | 0.001318 |
| 0.087817 | NA | 0.111904 | NA | NA | 0.088436 | 4 | -207.541 | 423.1526 | 12.53134 | 0.00107 |
| -0.07546 | NA | 0.104647 | 0.047691 | NA | 0.089281 | 5 | -206.681 | 423.4693 | 12.84806 | 0.000913 |
| 0.490367 | 0.055621 | NA | NA | -0.01646 | NA | 4 | -208.095 | 424.2617 | 13.64043 | 0.000614 |
| 0.44439 | 0.054626 | NA | NA | NA | NA | 3 | -209.324 | 424.6908 | 14.06956 | 0.000496 |
| 0.286531 | 0.05339 | NA | 0.040693 | NA | NA | 4 | -208.694 | 425.4589 | 14.83758 | 0.000338 |
| -0.05446 | NA | NA | 0.154929 | -0.03858 | 0.109385 | 5 | -208.603 | 427.3133 | 16.69207 | 0.000134 |
| 0.163913 | NA | NA | 0.070735 | NA | 0.110728 | 4 | -212.616 | 433.304 | 22.68268 | 6.68E-06 |
| 0.439961 | NA | NA | NA | NA | 0.111714 | 3 | -214.529 | 435.1 | 24.4787 | 2.72E-06 |
| 0.467925 | NA | NA | NA | -0.00979 | 0.111671 | 4 | -214.1 | 436.2703 | 25.64906 | 1.52E-06 |
| -0.42864 | NA | 0.159115 | 0.137283 | -0.04443 | NA | 5 | -213.185 | 436.4769 | 25.85557 | 1.37E-06 |
| 0.006058 | NA | 0.168569 | NA | -0.01945 | NA | 4 | -217.394 | 442.8584 | 32.23713 | 5.63E-08 |
| -0.02385 | NA | 0.160705 | NA | NA | NA | 3 | -219.027 | 444.0963 | 33.47505 | 3.03E-08 |
| -0.16657 | NA | 0.154808 | 0.041417 | NA | NA | 4 | -218.404 | 444.8788 | 34.25752 | 2.05E-08 |
| -0.03512 | NA | NA | 0.166682 | -0.04094 | NA | 4 | -228.07 | 464.211 | 53.58975 | 1.30E-12 |
| 0.197037 | NA | NA | 0.077423 | NA | NA | 3 | -232.291 | 470.6244 | 60.00314 | 5.26E-14 |
| 0.499762 | NA | NA | NA | NA | NA | 2 | -234.429 | 472.8798 | 62.25848 | 1.70E-14 |
| 0.528218 | NA | NA | NA | -0.00997 | NA | 3 | -234.014 | 474.0714 | 63.45011 | 9.39E-15 |

## **Table S29 – Model selection for forbs under control, colonisation, mean**

| (Intercept) | log(Nf) | log(Ng) | log(Nl + exp(-1)) | df | logLik | AICc | delta | weight |
| --- | --- | --- | --- | --- | --- | --- | --- | --- |
| -1.20115 | 0.16742 | 0.230141 | NA | 4 | -167.31 | 342.7867 | 0 | 0.63623 |
| **-1.25773** | **0.170522** | **0.249966** | **-0.011** | **5** | **-167.215** | **344.6791** | **1.892403** | **0.246993** |
| -0.34533 | 0.18402 | NA | NA | 3 | -170.524 | 347.1466 | 4.359885 | 0.071925 |
| -0.37172 | 0.177042 | NA | 0.016942 | 4 | -170.246 | 348.6586 | 5.871924 | 0.033771 |
| -0.81192 | NA | 0.269142 | NA | 3 | -172.753 | 351.6044 | 8.817758 | 0.007742 |
| -0.80822 | NA | 0.267603 | 0.000824 | 4 | -172.752 | 353.6702 | 10.88351 | 0.002756 |
| 0.251939 | NA | NA | NA | 2 | -177.011 | 358.0709 | 15.28427 | 0.000305 |
| 0.161321 | NA | NA | 0.031306 | 3 | -176.079 | 358.2568 | 15.47009 | 0.000278 |

## **Table S30 – Model selection for forbs under control, colonisation, s.d.**

| (Intercept) | log(Nf) | log(Ng) | log(Nl + exp(-1)) | df | logLik | AICc | delta | weight |
| --- | --- | --- | --- | --- | --- | --- | --- | --- |
| -1.06334 | **0.184727** | **0.207695** | **NA** | **4** | **-39.2382** | **86.64235** | **0** | **0.73006** |
| -1.08409 | 0.185865 | 0.214968 | -0.00404 | 5 | -39.2017 | 88.65335 | 2.011003 | 0.267101 |
| -0.32213 | 0.191472 | NA | 0.019996 | 4 | -45.4668 | 99.09959 | 12.45724 | 0.00144 |
| -0.29098 | 0.199708 | NA | NA | 3 | -46.529 | 99.15721 | 12.51486 | 0.001399 |
| -0.63388 | NA | 0.250728 | NA | 3 | -57.0706 | 120.2404 | 33.59803 | 3.70E-08 |
| -0.59414 | NA | 0.234191 | 0.008854 | 4 | -56.9155 | 121.9969 | 35.35457 | 1.54E-08 |
| 0.254353 | NA | NA | 0.035531 | 3 | -63.3664 | 132.8319 | 46.18958 | 6.81E-11 |
| 0.357199 | NA | NA | NA | 2 | -66.3429 | 136.7352 | 50.09281 | 9.68E-12 |

## **Table S31 – Model selection for grasses under drought, persistence**

| (Intercept) | I((log(size))^2) | log(Nf) | log(Ng) | log(Nl + exp(-1)) | log(size) | df | logLik | AICc | delta | weight |
| --- | --- | --- | --- | --- | --- | --- | --- | --- | --- | --- |
| 4.650234 | NA | -0.59584 | -0.5543 | NA | 0.470098 | 4 | -525.104 | 1058.255 | 0 | 0.433913 |
| **4.460571** | **NA** | **-0.59059** | **-0.48189** | **-0.03808** | **0.469091** | **5** | **-524.966** | **1060.003** | **1.748109** | **0.181053** |
| 4.722483 | 0.013908 | -0.61241 | -0.56113 | NA | 0.463647 | 5 | -525.062 | 1060.194 | 1.939047 | 0.164567 |
| 2.911628 | NA | -0.63037 | NA | -0.11546 | 0.477942 | 4 | -526.814 | 1061.676 | 3.420861 | 0.078446 |
| 4.532739 | 0.013672 | -0.60692 | -0.48899 | -0.0379 | 0.462763 | 6 | -524.926 | 1061.949 | 3.694643 | 0.06841 |
| 2.729579 | NA | -0.68079 | NA | NA | 0.487937 | 3 | -528.706 | 1063.44 | 5.185409 | 0.032464 |
| 2.928845 | 0.004815 | -0.63633 | NA | -0.11577 | 0.475786 | 5 | -526.809 | 1063.689 | 5.433993 | 0.02867 |
| 2.730654 | 0.000312 | -0.68118 | NA | NA | 0.487799 | 4 | -528.706 | 1065.459 | 7.204069 | 0.011832 |
| 3.44851 | NA | NA | -0.74213 | NA | 0.396921 | 3 | -533.665 | 1073.358 | 15.10329 | 0.000228 |
| 3.294233 | -0.05516 | NA | -0.69352 | NA | 0.426682 | 4 | -532.857 | 1073.76 | 15.50504 | 0.000186 |
| 3.191828 | NA | NA | -0.63595 | -0.05458 | 0.396056 | 4 | -533.37 | 1074.787 | 16.53201 | 0.000112 |
| 3.041124 | -0.0548 | NA | -0.58892 | -0.05378 | 0.425701 | 5 | -532.571 | 1075.212 | 16.95741 | 9.02E-05 |
| 0.996265 | -0.06954 | NA | NA | -0.14782 | 0.440269 | 4 | -535.368 | 1078.782 | 20.52716 | 1.51E-05 |
| 0.976146 | NA | NA | NA | -0.15769 | 0.404442 | 3 | -536.694 | 1079.417 | 21.16205 | 1.10E-05 |
| 0.559717 | -0.08136 | NA | NA | NA | 0.454257 | 3 | -538.666 | 1083.359 | 25.10441 | 1.53E-06 |
| 0.500804 | NA | NA | NA | NA | 0.414614 | 2 | -540.503 | 1085.021 | 26.7657 | 6.69E-07 |
| 5.024493 | 0.069916 | -0.52309 | -0.68128 | NA | NA | 4 | -539.909 | 1087.865 | 29.61065 | 1.61E-07 |
| 4.620948 | NA | -0.41321 | -0.65345 | NA | NA | 3 | -541.311 | 1088.651 | 30.39599 | 1.09E-07 |
| 4.807918 | 0.069466 | -0.51664 | -0.59875 | -0.04377 | NA | 5 | -539.724 | 1089.517 | 31.26242 | 7.06E-08 |
| 4.396644 | NA | -0.40727 | -0.56721 | -0.04571 | NA | 4 | -541.108 | 1090.262 | 32.00713 | 4.87E-08 |
| 2.841024 | 0.061904 | -0.55083 | NA | -0.13915 | NA | 4 | -542.632 | 1093.31 | 35.05557 | 1.06E-08 |
| 2.567309 | NA | -0.45163 | NA | -0.13653 | NA | 3 | -543.746 | 1093.52 | 35.26493 | 9.54E-09 |
| 3.777541 | NA | NA | -0.78745 | NA | NA | 2 | -545.917 | 1095.847 | 37.59249 | 2.98E-09 |
| 2.604686 | 0.05922 | -0.6044 | NA | NA | NA | 3 | -545.451 | 1096.93 | 38.67479 | 1.73E-09 |
| 2.347142 | NA | -0.50853 | NA | NA | NA | 2 | -546.475 | 1096.964 | 38.70908 | 1.71E-09 |
| 3.507683 | NA | NA | -0.67541 | -0.05801 | NA | 3 | -545.579 | 1097.187 | 38.93174 | 1.53E-09 |
| 3.796617 | 0.007671 | NA | -0.79427 | NA | NA | 3 | -545.896 | 1097.82 | 39.56481 | 1.11E-09 |
| 3.526945 | 0.007938 | NA | -0.68224 | -0.05814 | NA | 4 | -545.557 | 1099.161 | 40.90576 | 5.69E-10 |
| 1.158479 | NA | NA | NA | -0.16829 | NA | 2 | -549.422 | 1102.858 | 44.6029 | 8.95E-11 |
| 1.161762 | -0.00542 | NA | NA | -0.1675 | NA | 3 | -549.411 | 1104.85 | 46.59559 | 3.31E-11 |
| 0.653384 | NA | NA | NA | NA | NA | 1 | -553.837 | 1109.678 | 51.42303 | 2.96E-12 |
| 0.670144 | -0.01606 | NA | NA | NA | NA | 2 | -553.742 | 1111.498 | 53.24326 | 1.19E-12 |

## **Table S32 – Model selection for grasses under drought, expansion, mean**

| (Intercept) | I((log(size))^2) | log(Nf) | log(Ng) | log(Nl + exp(-1)) | log(size) | df | logLik | AICc | delta | weight |
| --- | --- | --- | --- | --- | --- | --- | --- | --- | --- | --- |
| **-0.41698** | **0.101694** | **NA** | **0.236052** | **-0.05655** | **0.164809** | **6** | **-532.917** | **1077.985** | **0** | **0.413991** |
| -0.60501 | 0.094735 | 0.071291 | 0.228364 | -0.05832 | 0.160956 | 7 | -532.109 | 1078.418 | 0.433405 | 0.333333 |
| -0.09796 | 0.101192 | NA | 0.11294 | NA | 0.167557 | 5 | -535.67 | 1081.447 | 3.461912 | 0.073324 |
| -0.25628 | 0.094989 | 0.063404 | 0.102663 | NA | 0.164207 | 6 | -535.035 | 1082.219 | 4.234458 | 0.04983 |
| 0.341808 | 0.106746 | NA | NA | NA | 0.160451 | 4 | -537.185 | 1082.442 | 4.456902 | 0.044585 |
| 0.104955 | 0.098735 | 0.075718 | NA | NA | 0.157222 | 5 | -536.266 | 1082.638 | 4.653755 | 0.040406 |
| 0.132471 | 0.100009 | 0.082471 | NA | -0.01715 | 0.153751 | 6 | -535.856 | 1083.861 | 5.876428 | 0.021925 |
| 0.380019 | 0.108308 | NA | NA | -0.01347 | 0.15795 | 5 | -536.928 | 1083.964 | 5.979174 | 0.020827 |
| -0.53087 | 0.141802 | 0.086731 | 0.208513 | -0.06126 | NA | 6 | -539.34 | 1090.83 | 12.84513 | 0.000673 |
| -0.29876 | 0.151689 | NA | 0.217333 | -0.05917 | NA | 5 | -540.512 | 1091.13 | 13.14566 | 0.000579 |
| 0.104542 | 0.14434 | 0.087487 | NA | NA | NA | 4 | -543.152 | 1094.375 | 16.39066 | 0.000114 |
| 0.379079 | 0.154711 | NA | NA | NA | NA | 3 | -544.353 | 1094.749 | 16.76473 | 9.47E-05 |
| 0.142091 | 0.144704 | 0.096345 | NA | -0.0234 | NA | 5 | -542.4 | 1094.908 | 16.92318 | 8.75E-05 |
| 0.037386 | 0.152037 | NA | 0.088082 | NA | NA | 4 | -543.447 | 1094.966 | 16.98085 | 8.50E-05 |
| -0.16265 | 0.143068 | 0.078766 | 0.075932 | NA | NA | 5 | -542.488 | 1095.083 | 17.09833 | 8.02E-05 |
| 0.432928 | 0.155877 | NA | NA | -0.01928 | NA | 4 | -543.837 | 1095.745 | 17.76046 | 5.76E-05 |
| -0.98944 | NA | 0.149084 | 0.268905 | -0.05896 | 0.292279 | 6 | -543.889 | 1099.927 | 21.94261 | 7.12E-06 |
| -0.63792 | NA | 0.141321 | 0.141932 | NA | 0.295922 | 5 | -546.758 | 1103.622 | 25.63729 | 1.12E-06 |
| -0.62616 | NA | NA | 0.293153 | -0.05503 | 0.32297 | 5 | -547.557 | 1105.222 | 27.23693 | 5.04E-07 |
| -0.15198 | NA | 0.162919 | NA | NA | 0.293408 | 4 | -549.046 | 1106.163 | 28.17784 | 3.15E-07 |
| -0.13782 | NA | 0.167531 | NA | -0.01004 | 0.292404 | 5 | -548.911 | 1107.928 | 29.94361 | 1.30E-07 |
| -0.31465 | NA | NA | 0.173051 | NA | 0.324884 | 4 | -550.035 | 1108.14 | 30.15553 | 1.17E-07 |
| 0.360697 | NA | NA | NA | NA | 0.327297 | 3 | -553.506 | 1113.055 | 35.07051 | 1.00E-08 |
| 0.362292 | NA | NA | NA | -0.00056 | 0.327294 | 4 | -553.506 | 1115.083 | 37.09821 | 3.64E-09 |
| -1.34627 | NA | 0.314258 | 0.269791 | -0.06889 | NA | 5 | -580.395 | 1170.897 | 92.91194 | 2.76E-21 |
| -0.9399 | NA | 0.307579 | 0.121138 | NA | NA | 4 | -583.842 | 1175.755 | 97.76999 | 2.44E-22 |
| -0.52245 | NA | 0.324829 | NA | NA | NA | 3 | -585.308 | 1176.659 | 98.6742 | 1.55E-22 |
| -0.49199 | NA | 0.332837 | NA | -0.01981 | NA | 4 | -584.844 | 1177.759 | 99.77394 | 8.94E-23 |
| -0.56906 | NA | NA | 0.327855 | -0.062 | NA | 4 | -596.266 | 1200.603 | 122.6184 | 9.79E-28 |
| -0.21746 | NA | NA | 0.192675 | NA | NA | 3 | -598.915 | 1203.873 | 125.8881 | 1.91E-28 |
| 0.535904 | NA | NA | NA | NA | NA | 2 | -602.539 | 1209.1 | 131.1152 | 1.40E-29 |
| 0.539005 | NA | NA | NA | -0.00109 | NA | 3 | -602.538 | 1211.119 | 133.1339 | 5.10E-30 |

## **Table S33 – Model selection for grasses under drought, expansion, s.d.**

| (Intercept) | I((log(size))^2) | log(Nf) | log(Ng) | log(Nl + exp(-1)) | log(size) | df | logLik | AICc | delta | weight |
| --- | --- | --- | --- | --- | --- | --- | --- | --- | --- | --- |
| **1.657017** | **NA** | **NA** | **-0.27601** | **NA** | **0.113981** | **4** | **-145.128** | **298.4714** | **0** | **0.326858** |
| 1.53731 | NA | 0.040507 | -0.2831 | NA | 0.112076 | 5 | -144.938 | 300.2 | 1.728648 | 0.137717 |
| 1.666376 | NA | NA | -0.27286 | -0.00746 | 0.112347 | 5 | -145.094 | 300.5127 | 2.041318 | 0.117786 |
| 1.647564 | -0.00568 | NA | -0.2745 | NA | 0.129751 | 5 | -145.095 | 300.5134 | 2.04206 | 0.117742 |
| 1.703659 | 0.030422 | NA | -0.27273 | NA | NA | 4 | -146.837 | 301.8884 | 3.41706 | 0.059204 |
| 1.538347 | NA | 0.044736 | -0.27929 | -0.01079 | 0.109513 | 6 | -144.869 | 302.1939 | 3.722567 | 0.050818 |
| 1.52753 | -0.00574 | 0.040583 | -0.28159 | NA | 0.128009 | 6 | -144.903 | 302.2634 | 3.792063 | 0.049082 |
| 1.656925 | -0.00561 | NA | -0.2714 | -0.00737 | 0.127946 | 6 | -145.061 | 302.5793 | 4.107899 | 0.041912 |
| 1.568165 | 0.029811 | 0.045525 | -0.2807 | NA | NA | 5 | -146.6 | 303.5246 | 5.053199 | 0.026126 |
| 1.718413 | 0.029676 | NA | -0.26745 | -0.01267 | NA | 5 | -146.739 | 303.803 | 5.331644 | 0.02273 |
| 1.528717 | -0.00565 | 0.044777 | -0.27783 | -0.0107 | 0.125209 | 7 | -144.835 | 304.2828 | 5.811456 | 0.017882 |
| 1.568622 | 0.028762 | 0.051784 | -0.27497 | -0.01639 | NA | 6 | -146.441 | 305.339 | 6.867658 | 0.010545 |
| 0.589091 | NA | NA | NA | NA | 0.097241 | 3 | -150.466 | 307.0613 | 8.589893 | 0.004457 |
| 1.641562 | NA | NA | -0.23197 | NA | NA | 3 | -150.914 | 307.9568 | 9.485473 | 0.002848 |
| 0.64913 | NA | NA | NA | -0.02078 | 0.09322 | 4 | -150.211 | 308.6381 | 10.16668 | 0.002026 |
| 0.582309 | -0.01072 | NA | NA | NA | 0.127175 | 4 | -150.353 | 308.9204 | 10.44903 | 0.001759 |
| 0.551817 | NA | 0.010261 | NA | NA | 0.09665 | 4 | -150.455 | 309.1245 | 10.65309 | 0.001589 |
| 1.461041 | NA | 0.061217 | -0.2438 | NA | NA | 4 | -150.501 | 309.2172 | 10.74582 | 0.001517 |
| 1.674087 | NA | NA | -0.22342 | -0.02532 | NA | 4 | -150.532 | 309.2783 | 10.80694 | 0.001471 |
| 0.644038 | 0.024703 | NA | NA | NA | NA | 3 | -151.938 | 310.0047 | 11.53334 | 0.001023 |
| 1.468756 | NA | 0.071637 | -0.2357 | -0.02993 | NA | 5 | -149.976 | 310.276 | 11.80464 | 0.000893 |
| 0.641713 | -0.01037 | NA | NA | -0.02049 | 0.12224 | 5 | -150.105 | 310.5338 | 12.06244 | 0.000785 |
| 0.581557 | NA | 0.01989 | NA | -0.0224 | 0.09176 | 5 | -150.169 | 310.6618 | 12.19044 | 0.000737 |
| 0.543375 | -0.01077 | 0.010709 | NA | NA | 0.126698 | 5 | -150.34 | 311.004 | 12.53266 | 0.000621 |
| 0.714645 | 0.023432 | NA | NA | -0.02537 | NA | 4 | -151.56 | 311.3355 | 12.86417 | 0.000526 |
| 0.586648 | 0.024435 | 0.015693 | NA | NA | NA | 4 | -151.911 | 312.0372 | 13.56581 | 0.00037 |
| 0.573014 | -0.01044 | 0.020208 | NA | -0.02213 | 0.120944 | 6 | -150.061 | 312.5777 | 14.10629 | 0.000283 |
| 0.724452 | NA | NA | NA | NA | NA | 2 | -154.581 | 313.225 | 14.75364 | 0.000204 |
| 0.621058 | 0.022859 | 0.027222 | NA | -0.02752 | NA | 5 | -151.481 | 313.2863 | 14.81492 | 0.000198 |
| 0.813546 | NA | NA | NA | -0.03401 | NA | 3 | -153.907 | 313.9415 | 15.47014 | 0.000143 |
| 0.605046 | NA | 0.032161 | NA | NA | NA | 3 | -154.468 | 315.0652 | 16.59385 | 8.15E-05 |
| 0.650083 | NA | 0.046349 | NA | -0.0373 | NA | 4 | -153.678 | 315.5714 | 17.10007 | 6.33E-05 |

## **Table S34 – Model selection for grasses under drought, colonisation, mean**

| (Intercept) | log(Nf) | log(Ng) | log(Nl + exp(-1)) | df | logLik | AICc | delta | weight |
| --- | --- | --- | --- | --- | --- | --- | --- | --- |
| -1.20115 | 0.16742 | 0.230141 | NA | 4 | -167.31 | 342.7867 | 0 | 0.63623 |
| -1.25773 | 0.170522 | 0.249966 | -0.011 | 5 | -167.215 | 344.6791 | 1.892403 | 0.246993 |
| -0.34533 | 0.18402 | NA | NA | 3 | -170.524 | 347.1466 | 4.359885 | 0.071925 |
| -0.37172 | 0.177042 | NA | 0.016942 | 4 | -170.246 | 348.6586 | 5.871924 | 0.033771 |
| -0.81192 | NA | 0.269142 | NA | 3 | -172.753 | 351.6044 | 8.817758 | 0.007742 |
| -0.80822 | NA | 0.267603 | 0.000824 | 4 | -172.752 | 353.6702 | 10.88351 | 0.002756 |
| 0.251939 | NA | NA | NA | 2 | -177.011 | 358.0709 | 15.28427 | 0.000305 |
| 0.161321 | NA | NA | 0.031306 | 3 | -176.079 | 358.2568 | 15.47009 | 0.000278 |

## **Table S35 – Model selection for grasses under drought, colonisation, s.d.**

| (Intercept) | log(Nf) | log(Ng) | log(Nl + exp(-1)) | df | logLik | AICc | delta | weight |
| --- | --- | --- | --- | --- | --- | --- | --- | --- |
| **0.76469** | **NA** | **NA** | **NA** | **2** | **-46.4661** | **97.17216** | **0** | **0.313883** |
| 1.519376 | -0.20374 | NA | NA | 3 | -45.5688 | 97.62742 | 0.455259 | 0.249983 |
| 1.039492 | NA | -0.07409 | NA | 3 | -46.3508 | 99.1914 | 2.019241 | 0.114366 |
| 0.793456 | NA | NA | -0.01322 | 3 | -46.4201 | 99.33005 | 2.157896 | 0.106706 |
| 1.584339 | -0.19783 | -0.02342 | NA | 4 | -45.5577 | 99.94871 | 2.776548 | 0.078316 |
| 1.519165 | -0.20352 | NA | -0.00029 | 4 | -45.5688 | 99.97091 | 2.798754 | 0.077451 |
| 1.034316 | NA | -0.06726 | -0.00926 | 4 | -46.3291 | 101.4916 | 4.319436 | 0.036209 |
| 1.586166 | -0.19833 | -0.02387 | 0.000786 | 5 | -45.5575 | 102.3917 | 5.2195 | 0.023087 |

## **Table S36 – Model selection for legumes under drought, persistence**

| (Intercept) | I((log(size))^2) | log(Nf) | log(Ng) | log(Nl + exp(-1)) | log(size) | df | logLik | AICc | delta | weight |
| --- | --- | --- | --- | --- | --- | --- | --- | --- | --- | --- |
| 6.694131 | NA | NA | -1.49037 | NA | NA | 2 | -70.457 | 145.0193 | 0 | 0.155425 |
| **5.699385** | **NA** | **0.496715** | **-1.70306** | **NA** | **NA** | **3** | **-69.492** | **145.1963** | **0.177006** | **0.14226** |
| 7.116145 | NA | 0.541325 | -1.85027 | -0.31827 | NA | 4 | -68.8482 | 146.0535 | 1.034162 | 0.092673 |
| 7.955481 | NA | NA | -1.5899 | -0.27778 | NA | 3 | -69.9639 | 146.1402 | 1.120904 | 0.08874 |
| 5.883208 | -0.05704 | 0.546837 | -1.75675 | NA | NA | 4 | -69.0066 | 146.3703 | 1.35106 | 0.079094 |
| 6.916535 | -0.04548 | NA | -1.51522 | NA | NA | 3 | -70.1442 | 146.5008 | 1.481557 | 0.074098 |
| 6.802476 | NA | NA | -1.49125 | NA | -0.08962 | 3 | -70.3335 | 146.8794 | 1.86015 | 0.061319 |
| 5.777075 | NA | 0.522031 | -1.71153 | NA | -0.11828 | 4 | -69.2823 | 146.9217 | 1.902397 | 0.060037 |
| 6.924032 | -0.03734 | 0.565739 | -1.85268 | -0.2504 | NA | 5 | -68.6688 | 147.8782 | 2.858933 | 0.037214 |
| 7.849848 | -0.0271 | NA | -1.58516 | -0.22652 | NA | 4 | -69.8691 | 148.0954 | 3.076064 | 0.033386 |
| 7.026496 | NA | 0.547909 | -1.84104 | -0.29192 | -0.04661 | 5 | -68.8198 | 148.1802 | 3.160947 | 0.031998 |
| 7.920061 | NA | NA | -1.58497 | -0.2638 | -0.02384 | 4 | -69.9564 | 148.2699 | 3.250592 | 0.030596 |
| 5.895835 | -0.09732 | 0.55131 | -1.77946 | NA | 0.145886 | 5 | -68.9249 | 148.3903 | 3.371014 | 0.028808 |
| 6.925145 | -0.08289 | NA | -1.5319 | NA | 0.135087 | 4 | -70.0751 | 148.5074 | 3.48808 | 0.02717 |
| 7.003704 | -0.08591 | 0.572475 | -1.88694 | -0.26539 | 0.180283 | 6 | -68.5461 | 149.8558 | 4.836557 | 0.013844 |
| 7.915397 | -0.07207 | NA | -1.60956 | -0.24012 | 0.166326 | 5 | -69.7661 | 150.0727 | 5.053427 | 0.012422 |
| 0.693147 | NA | NA | NA | NA | NA | 1 | -74.4722 | 150.9791 | 5.959809 | 0.007895 |
| -0.2816 | NA | 0.260575 | NA | NA | NA | 2 | -74.1859 | 152.477 | 7.45773 | 0.003733 |
| 0.765359 | -0.02597 | NA | NA | NA | NA | 2 | -74.351 | 152.8072 | 7.787944 | 0.003165 |
| 1.055642 | NA | NA | NA | -0.11655 | NA | 2 | -74.3599 | 152.825 | 7.805704 | 0.003137 |
| 0.765838 | NA | NA | NA | NA | -0.06056 | 2 | -74.4091 | 152.9234 | 7.904121 | 0.002986 |
| -0.26865 | -0.02998 | 0.279369 | NA | NA | NA | 3 | -74.0254 | 154.2632 | 9.243876 | 0.001528 |
| 0.078484 | NA | 0.264788 | NA | -0.12088 | NA | 3 | -74.0641 | 154.3407 | 9.321369 | 0.00147 |
| -0.24704 | NA | 0.275115 | NA | NA | -0.07412 | 3 | -74.0931 | 154.3986 | 9.379295 | 0.001428 |
| 0.999639 | -0.01901 | NA | NA | -0.08155 | NA | 3 | -74.3048 | 154.8219 | 9.802642 | 0.001156 |
| 0.740216 | -0.04151 | NA | NA | NA | 0.056952 | 3 | -74.3379 | 154.8881 | 9.868811 | 0.001118 |
| 1.036564 | NA | NA | NA | -0.09737 | -0.0338 | 3 | -74.3432 | 154.8987 | 9.879423 | 0.001112 |
| -0.03794 | -0.02328 | 0.278139 | NA | -0.0787 | NA | 4 | -73.9818 | 156.3208 | 11.30155 | 0.000546 |
| -0.28854 | -0.04422 | 0.278509 | NA | NA | 0.052281 | 4 | -74.0147 | 156.3865 | 11.36722 | 0.000529 |
| 0.020688 | NA | 0.273474 | NA | -0.09416 | -0.04813 | 4 | -74.0308 | 156.4187 | 11.39946 | 0.00052 |
| 0.985381 | -0.03755 | NA | NA | -0.08731 | 0.069734 | 4 | -74.2853 | 156.9278 | 11.90854 | 0.000403 |
| -0.04733 | -0.0404 | 0.2769 | NA | -0.08374 | 0.064393 | 5 | -73.9658 | 158.4722 | 13.45287 | 0.000186 |

## **Table S37 – Model selection for legumes under drought, expansion, mean**

| (Intercept) | I((log(size))^2) | log(Nf) | log(Ng) | log(Nl + exp(-1)) | log(size) | df | logLik | AICc | delta | weight |
| --- | --- | --- | --- | --- | --- | --- | --- | --- | --- | --- |
| 0.725033 | 0.194524 | NA | NA | NA | NA | 3 | -96.356 | 199.0363 | 0 | 0.148441 |
| -0.10803 | 0.192919 | 0.221306 | NA | NA | NA | 4 | -95.4309 | 199.4098 | 0.373417 | 0.12316 |
| 0.60469 | 0.121688 | NA | NA | NA | 0.267402 | 4 | -95.687 | 199.922 | 0.885632 | 0.095333 |
| -0.0523 | 0.201486 | NA | 0.194873 | NA | NA | 4 | -95.8624 | 200.2727 | 1.236316 | 0.08 |
| 0.471343 | NA | NA | NA | NA | 0.655802 | 3 | -97.291 | 200.9064 | 1.870043 | 0.058275 |
| **-0.07027** | **0.137617** | **0.186893** | **NA** | **NA** | **0.203943** | **5** | **-95.0567** | **200.9467** | **1.910324** | **0.057113** |
| 0.916458 | 0.200144 | NA | NA | -0.06708 | NA | 4 | -96.2066 | 200.9612 | 1.924866 | 0.056699 |
| -0.61483 | 0.198431 | 0.197537 | 0.149483 | NA | NA | 5 | -95.1451 | 201.1235 | 2.087177 | 0.052279 |
| 0.083895 | 0.198587 | 0.221639 | NA | -0.06769 | NA | 5 | -95.2751 | 201.3836 | 2.347225 | 0.045905 |
| -0.04584 | 0.135164 | NA | 0.166208 | NA | 0.239726 | 5 | -95.3296 | 201.4925 | 2.456184 | 0.043471 |
| 0.809703 | 0.126325 | NA | NA | -0.07268 | 0.272734 | 5 | -95.5088 | 201.851 | 2.814618 | 0.036339 |
| 0.137236 | 0.205147 | NA | 0.182421 | -0.04901 | NA | 5 | -95.7837 | 202.4007 | 3.364401 | 0.027605 |
| 0.035826 | NA | 0.117552 | NA | NA | 0.647866 | 4 | -97.0422 | 202.6323 | 3.595969 | 0.024587 |
| -0.52658 | 0.146876 | 0.168324 | 0.133718 | NA | 0.187982 | 6 | -94.8282 | 202.8396 | 3.803229 | 0.022166 |
| 0.134582 | 0.142155 | 0.186324 | NA | -0.0719 | 0.209411 | 6 | -94.8795 | 202.942 | 3.905667 | 0.02106 |
| 0.162552 | NA | NA | 0.077144 | NA | 0.662919 | 4 | -97.2132 | 202.9744 | 3.938092 | 0.020721 |
| 0.599407 | NA | NA | NA | -0.04655 | 0.668696 | 4 | -97.22 | 202.988 | 3.951645 | 0.020581 |
| -0.4123 | 0.202444 | 0.200089 | 0.135113 | -0.05425 | NA | 6 | -95.047 | 203.2772 | 4.240813 | 0.01781 |
| 0.175541 | 0.137569 | NA | 0.150867 | -0.0572 | 0.246477 | 6 | -95.2214 | 203.6259 | 4.589515 | 0.014961 |
| 0.163432 | NA | 0.115803 | NA | -0.04403 | 0.66018 | 5 | -96.9783 | 204.79 | 5.753618 | 0.008359 |
| -0.13612 | NA | 0.108657 | 0.051188 | NA | 0.653189 | 5 | -97.0092 | 204.8517 | 5.81532 | 0.008105 |
| -0.29979 | 0.149522 | 0.17013 | 0.117296 | -0.05993 | 0.1945 | 7 | -94.7079 | 205.0159 | 5.979552 | 0.007466 |
| 0.315258 | NA | NA | 0.065661 | -0.0388 | 0.672608 | 5 | -97.1656 | 205.1644 | 6.128098 | 0.006932 |
| 0.018117 | NA | 0.10914 | 0.039376 | -0.03953 | 0.663016 | 6 | -96.9594 | 207.1019 | 8.065588 | 0.002631 |
| 0.338511 | NA | NA | NA | 0.290928 | NA | 3 | -117.572 | 241.4673 | 42.431 | 9.07E-11 |
| -0.68671 | NA | 0.274407 | NA | 0.28672 | NA | 4 | -116.745 | 242.0389 | 43.00254 | 6.82E-11 |
| 1.23233 | NA | NA | NA | NA | NA | 2 | -119.441 | 243.0417 | 44.00533 | 4.13E-11 |
| 0.864361 | NA | NA | -0.12086 | 0.273028 | NA | 4 | -117.46 | 243.4681 | 44.43174 | 3.34E-11 |
| 0.159527 | NA | 0.283568 | NA | NA | NA | 3 | -118.599 | 243.5232 | 44.48684 | 3.25E-11 |
| 0.018456 | NA | 0.302723 | -0.18639 | 0.25868 | NA | 5 | -116.483 | 243.7996 | 44.7633 | 2.83E-11 |
| 2.14242 | NA | NA | -0.23361 | NA | NA | 3 | -119.016 | 244.3572 | 45.32087 | 2.14E-11 |
| 1.154868 | NA | 0.327417 | -0.29808 | NA | NA | 4 | -117.912 | 244.3711 | 45.33472 | 2.12E-11 |

## **Table S38 – Model selection for legumes under drought, expansion, s.d.**

| (Intercept) | I((log(size))^2) | log(Nf) | log(Ng) | log(Nl + exp(-1)) | log(size) | df | logLik | AICc | delta | weight |
| --- | --- | --- | --- | --- | --- | --- | --- | --- | --- | --- |
| -0.34308 | NA | NA | 0.230221 | NA | 0.097414 | 4 | -48.9458 | 106.4395 | 0 | 0.142448 |
| -0.25721 | -0.0557 | NA | 0.19352 | NA | 0.271803 | 5 | -47.8541 | 106.5416 | 0.102145 | 0.135355 |
| **0.500219** | **-0.07139** | **NA** | **NA** | **NA** | **0.304026** | **4** | **-49.4648** | **107.4775** | **1.037995** | **0.084773** |
| -0.05961 | -0.06051 | -0.06919 | 0.206874 | NA | 0.293072 | 6 | -47.5688 | 108.3207 | 1.881203 | 0.055611 |
| -0.05215 | NA | NA | 0.184556 | NA | NA | 3 | -51.0619 | 108.4481 | 2.008637 | 0.052178 |
| -0.22048 | NA | -0.0446 | 0.240876 | NA | 0.101409 | 5 | -48.8273 | 108.488 | 2.048527 | 0.051147 |
| -0.31167 | NA | NA | 0.227859 | -0.00798 | 0.099407 | 5 | -48.9388 | 108.711 | 2.271483 | 0.045752 |
| -0.26453 | 0.019498 | NA | 0.226021 | NA | NA | 4 | -50.1167 | 108.7813 | 2.341831 | 0.044171 |
| 0.578447 | NA | NA | NA | NA | 0.076172 | 3 | -51.266 | 108.8564 | 2.416905 | 0.042543 |
| -0.25513 | -0.05568 | NA | 0.193375 | -0.00054 | 0.271867 | 6 | -47.8541 | 108.8913 | 2.451846 | 0.041806 |
| 0.666836 | NA | NA | NA | NA | NA | 2 | -52.554 | 109.268 | 2.828473 | 0.034631 |
| 0.646336 | -0.07484 | -0.04046 | NA | NA | 0.317764 | 5 | -49.3688 | 109.5709 | 3.131427 | 0.029763 |
| 0.557716 | -0.07009 | NA | NA | -0.02038 | 0.305522 | 5 | -49.419 | 109.6713 | 3.231835 | 0.028306 |
| -0.23052 | NA | NA | 0.200292 | 0.038105 | NA | 4 | -50.8918 | 110.3315 | 3.892063 | 0.020347 |
| -0.02005 | NA | -0.01064 | 0.186652 | NA | NA | 4 | -51.0553 | 110.6586 | 4.219112 | 0.017278 |
| -0.06178 | -0.06054 | -0.0692 | 0.207031 | 0.000572 | 0.293009 | 7 | -47.5688 | 110.7375 | 4.298031 | 0.016609 |
| 0.637044 | 0.011424 | NA | NA | NA | NA | 3 | -52.2186 | 110.7616 | 4.32211 | 0.01641 |
| 0.674393 | NA | NA | NA | -0.03488 | 0.085833 | 4 | -51.1362 | 110.8204 | 4.380877 | 0.015935 |
| -0.19049 | NA | -0.04451 | 0.238579 | -0.00769 | 0.103319 | 6 | -48.8209 | 110.8248 | 4.385361 | 0.0159 |
| -0.1972 | 0.019863 | -0.02364 | 0.231454 | NA | NA | 5 | -50.0838 | 111.0009 | 4.561387 | 0.01456 |
| -0.29738 | 0.018863 | NA | 0.228179 | 0.008494 | NA | 5 | -50.1091 | 111.0514 | 4.611965 | 0.014196 |
| 0.588639 | NA | -0.00275 | NA | NA | 0.076358 | 4 | -51.2656 | 111.0791 | 4.639642 | 0.014001 |
| 0.603218 | NA | 0.016816 | NA | NA | NA | 3 | -52.5377 | 111.3997 | 4.960258 | 0.011927 |
| 0.640904 | NA | NA | NA | 0.008441 | NA | 3 | -52.5454 | 111.4152 | 4.97572 | 0.011836 |
| 0.704902 | -0.07354 | -0.04062 | NA | -0.02055 | 0.319327 | 6 | -49.3221 | 111.8274 | 5.387875 | 0.009631 |
| -0.19043 | NA | -0.01434 | 0.203397 | 0.038785 | NA | 5 | -50.8799 | 112.593 | 6.153568 | 0.006568 |
| 0.677306 | 0.012606 | NA | NA | -0.01411 | NA | 4 | -52.1982 | 112.9443 | 6.504817 | 0.00551 |
| 0.587507 | 0.011328 | 0.01316 | NA | NA | NA | 4 | -52.2086 | 112.9652 | 6.525679 | 0.005453 |
| 0.689978 | NA | -0.00414 | NA | -0.03497 | 0.086137 | 5 | -51.1352 | 113.1037 | 6.664258 | 0.005088 |
| -0.23126 | 0.019189 | -0.02407 | 0.233871 | 0.009124 | NA | 6 | -50.075 | 113.333 | 6.893555 | 0.004537 |
| 0.579056 | NA | 0.016554 | NA | 0.008187 | NA | 4 | -52.5297 | 113.6073 | 7.167815 | 0.003955 |
| 0.627611 | 0.012513 | 0.013229 | NA | -0.01414 | NA | 5 | -52.188 | 115.2094 | 8.76993 | 0.001775 |

## **Table S39 – Model selection for legumes under drought, colonisation, mean**

| (Intercept) | log(Nf) | log(Ng) | log(Nl + exp(-1)) | df | logLik | AICc | delta | weight |
| --- | --- | --- | --- | --- | --- | --- | --- | --- |
| 0.808066 | NA | NA | -0.14503 | 3 | -24.6456 | 56.55444 | 0 | 0.238435 |
| **-0.57963** | **NA** | **0.380077** | **-0.14611** | **4** | **-23.4161** | **57.05448** | **0.500032** | **0.18569** |
| 0.514641 | NA | NA | NA | 2 | -26.3062 | 57.21246 | 0.658012 | 0.171587 |
| -0.85934 | NA | 0.375732 | NA | 3 | -25.2749 | 57.81298 | 1.258541 | 0.127081 |
| -0.77815 | 0.440984 | NA | -0.16737 | 4 | -23.8289 | 57.88001 | 1.325571 | 0.122893 |
| -1.66965 | 0.351333 | 0.332498 | -0.16377 | 5 | -22.8666 | 59.26268 | 2.70824 | 0.061558 |
| -0.49316 | 0.272416 | NA | NA | 3 | -26.0285 | 59.32013 | 2.765684 | 0.059815 |
| -1.44037 | 0.181641 | 0.350862 | NA | 4 | -25.1455 | 60.51315 | 3.958707 | 0.032942 |

## **Table S40 – Model selection for legumes under drought, colonisation, s.d.**

| (Intercept) | log(Nf) | log(Ng) | log(Nl + exp(-1)) | df | logLik | AICc | delta | weight |
| --- | --- | --- | --- | --- | --- | --- | --- | --- |
| 0.779809 | NA | NA | -0.10969 | 3 | -5.31827 | 17.8997 | 0 | 0.495113 |
| 0.334969 | NA | 0.121838 | -0.11004 | 4 | -4.65624 | 19.5347 | 1.635005 | 0.218609 |
| **0.230013** | **0.152849** | **NA** | **-0.11744** | **4** | **-4.79814** | **19.8185** | **1.918799** | **0.189689** |
| -0.05144 | 0.124546 | 0.104971 | -0.1163 | 5 | -4.30638 | 22.14217 | 4.242467 | 0.059356 |
| 0.557885 | NA | NA | NA | 2 | -9.80415 | 24.2083 | 6.308606 | 0.021126 |
| 0.124315 | NA | 0.118565 | NA | 3 | -9.38408 | 26.03132 | 8.131618 | 0.008491 |
| 0.429972 | 0.034576 | NA | NA | 3 | -9.78557 | 26.8343 | 8.934604 | 0.005683 |
| 0.111379 | 0.004044 | 0.118011 | NA | 4 | -9.38383 | 28.98987 | 11.09017 | 0.001934 |

## **Table S41 – Model selection for forbs under drought, persistence**

| (Intercept) | I((log(size))^2) | log(Nf) | log(Ng) | log(Nl + exp(-1)) | log(size) | df | logLik | AICc | delta | weight |
| --- | --- | --- | --- | --- | --- | --- | --- | --- | --- | --- |
| 3.821004 | 0.108906 | -0.829216197 | NA | -0.269851238 | 0.191941 | 5 | -251.085 | 512.3181 | 0 | 0.168396 |
| 2.292898 | NA | -0.798952725 | 0.406474 | -0.328069746 | 0.276588 | 5 | -251.086 | 512.3206 | 0.002515 | 0.168184 |
| 2.778272 | 0.096427 | -0.920293787 | 0.360219 | -0.318481809 | 0.222769 | 6 | -250.17 | 512.548 | 0.22989 | 0.15011 |
| **3.42127** | **NA** | **-0.677721031** | **NA** | **-0.273677524** | **0.250657** | **4** | **-252.283** | **512.6652** | **0.347152** | **0.141562** |
| 3.800954 | 0.138702 | -0.812236661 | NA | -0.264170764 | NA | 4 | -252.284 | 512.6677 | 0.349632 | 0.141387 |
| 2.997597 | 0.132809 | -0.879872919 | 0.275839 | -0.299984819 | NA | 5 | -251.731 | 513.6102 | 1.292103 | 0.088258 |
| 3.223914 | NA | -0.587916815 | NA | -0.268163561 | NA | 3 | -254.503 | 515.0648 | 2.74671 | 0.042647 |
| 2.316704 | NA | -0.677219314 | 0.320458 | -0.309291782 | NA | 4 | -253.74 | 515.5794 | 3.261351 | 0.032971 |
| 3.363025 | 0.113288 | -0.919976919 | NA | NA | 0.18176 | 4 | -254.451 | 517.0006 | 4.682535 | 0.016201 |
| 3.354631 | 0.141712 | -0.902447368 | NA | NA | NA | 3 | -255.532 | 517.1225 | 4.804403 | 0.015243 |
| 2.94552 | NA | -0.765415254 | NA | NA | 0.244221 | 3 | -255.758 | 517.5754 | 5.257292 | 0.012154 |
| 3.072429 | 0.110037 | -0.947748178 | 0.093273 | NA | 0.189141 | 5 | -254.374 | 518.8974 | 6.579354 | 0.006275 |
| 3.235141 | 0.140854 | -0.913540244 | 0.038284 | NA | NA | 4 | -255.518 | 519.136 | 6.817882 | 0.00557 |
| 2.537237 | NA | -0.812234927 | 0.136391 | NA | 0.251987 | 4 | -255.59 | 519.2792 | 6.961131 | 0.005185 |
| 2.760953 | NA | -0.675333034 | NA | NA | NA | 2 | -257.872 | 519.7742 | 7.456144 | 0.004048 |
| 2.524513 | NA | -0.70063646 | 0.077973 | NA | NA | 3 | -257.817 | 521.6927 | 9.374657 | 0.001551 |
| 1.524412 | NA | NA | NA | -0.347790198 | NA | 2 | -261.821 | 527.6724 | 15.35428 | 7.80E-05 |
| 1.494988 | NA | NA | NA | -0.355930796 | 0.120855 | 3 | -261.259 | 528.5776 | 16.25951 | 4.96E-05 |
| 1.900435 | NA | NA | -0.10139 | -0.333081345 | NA | 3 | -261.733 | 529.5262 | 17.20809 | 3.09E-05 |
| 1.536244 | -0.02156 | NA | NA | -0.343967539 | NA | 3 | -261.741 | 529.5418 | 17.22374 | 3.06E-05 |
| 1.513285 | -0.05106 | NA | NA | -0.349713852 | 0.160187 | 4 | -260.879 | 529.8566 | 17.53856 | 2.62E-05 |
| 1.843533 | NA | NA | -0.09385 | -0.342233371 | 0.119169 | 4 | -261.184 | 530.4671 | 18.14902 | 1.93E-05 |
| 1.828846 | -0.01632 | NA | -0.07972 | -0.333270511 | NA | 4 | -261.691 | 531.482 | 19.16386 | 1.16E-05 |
| 1.609941 | -0.04892 | NA | -0.02629 | -0.346077142 | 0.158082 | 5 | -260.874 | 531.896 | 19.57789 | 9.44E-06 |
| 2.039769 | NA | NA | -0.36147 | NA | NA | 2 | -266.794 | 537.618 | 25.29988 | 5.40E-07 |
| 0.556735 | NA | NA | NA | NA | NA | 1 | -268.244 | 538.4984 | 26.18029 | 3.48E-07 |
| 2.003249 | NA | NA | -0.36176 | NA | 0.08745 | 3 | -266.491 | 539.0416 | 26.72354 | 2.65E-07 |
| 1.973557 | -0.01626 | NA | -0.34126 | NA | NA | 3 | -266.752 | 539.5628 | 27.24473 | 2.04E-07 |
| 0.60053 | -0.04242 | NA | NA | NA | NA | 2 | -267.927 | 539.8833 | 27.5652 | 1.74E-07 |
| 0.519089 | NA | NA | NA | NA | 0.087901 | 2 | -267.944 | 539.9184 | 27.60035 | 1.71E-07 |
| 0.566224 | -0.06887 | NA | NA | NA | 0.141953 | 3 | -267.237 | 540.5339 | 28.21584 | 1.26E-07 |
| 1.825052 | -0.04067 | NA | -0.31162 | NA | 0.119607 | 4 | -266.272 | 540.6421 | 28.32396 | 1.19E-07 |

## **Table S42 – Model selection for forbs under drought, expansion, mean**

| (Intercept) | I((log(size))^2) | log(Nf) | log(Ng) | log(Nl + exp(-1)) | log(size) | df | logLik | AICc | delta | weight |
| --- | --- | --- | --- | --- | --- | --- | --- | --- | --- | --- |
| **-0.09385** | **0.116875** | **NA** | **0.162862** | **-0.07483** | **0.218547** | **6** | **-358.809** | **729.8689** | **0** | **0.442293** |
| 0.194516 | 0.126714 | -0.10147 | 0.179365 | -0.06993 | 0.213184 | 7 | -358.08 | 730.497 | 0.628141 | 0.323081 |
| 0.498875 | 0.12271 | NA | NA | -0.05984 | 0.20802 | 5 | -361.218 | 732.6141 | 2.745255 | 0.112095 |
| 0.721816 | 0.129384 | -0.06493 | NA | -0.05574 | 0.203906 | 6 | -360.914 | 734.0791 | 4.210243 | 0.053885 |
| 0.36303 | 0.128929 | -0.14039 | 0.128417 | NA | 0.212877 | 6 | -362.033 | 736.3168 | 6.447978 | 0.017601 |
| 0.355384 | 0.119268 | NA | NA | NA | 0.213216 | 4 | -364.39 | 736.8984 | 7.02953 | 0.01316 |
| -0.03017 | 0.115161 | NA | 0.099883 | NA | 0.220471 | 5 | -363.43 | 737.0386 | 7.169748 | 0.012269 |
| 0.737664 | 0.130616 | -0.10662 | NA | NA | 0.205876 | 5 | -363.544 | 737.2681 | 7.399198 | 0.010939 |
| 0.040226 | 0.185321 | NA | 0.144818 | -0.07561 | NA | 5 | -364.299 | 738.7777 | 8.908849 | 0.005143 |
| 0.377448 | 0.194974 | -0.12003 | 0.164863 | -0.0698 | NA | 6 | -363.307 | 738.8657 | 8.996853 | 0.004921 |
| 0.564338 | 0.18759 | NA | NA | -0.06218 | NA | 4 | -366.156 | 740.432 | 10.56315 | 0.002249 |
| 0.856485 | 0.194696 | -0.08558 | NA | -0.05671 | NA | 5 | -365.64 | 741.4597 | 11.59082 | 0.001345 |
| 0.545378 | 0.197086 | -0.15885 | 0.114033 | NA | NA | 5 | -367.127 | 744.4331 | 14.5642 | 0.000304 |
| 0.873935 | 0.196592 | -0.12821 | NA | NA | NA | 4 | -368.289 | 744.698 | 14.82912 | 0.000266 |
| 0.416792 | 0.185696 | NA | NA | NA | NA | 3 | -369.485 | 745.0421 | 15.17328 | 0.000224 |
| 0.105772 | 0.184198 | NA | 0.081012 | NA | NA | 4 | -368.869 | 745.8578 | 15.98892 | 0.000149 |
| -0.24298 | NA | NA | 0.198131 | -0.07236 | 0.459904 | 5 | -369.019 | 748.2176 | 18.34875 | 4.58E-05 |
| -0.29773 | NA | 0.020142 | 0.194266 | -0.07338 | 0.456935 | 6 | -368.99 | 750.2305 | 20.36166 | 1.68E-05 |
| 0.476837 | NA | NA | NA | -0.05378 | 0.461777 | 4 | -372.404 | 752.9272 | 23.05839 | 4.35E-06 |
| -0.17925 | NA | NA | 0.136698 | NA | 0.45834 | 4 | -373.098 | 754.3152 | 24.44638 | 2.17E-06 |
| 0.26313 | NA | 0.062574 | NA | -0.05805 | 0.452441 | 5 | -372.11 | 754.3994 | 24.53056 | 2.08E-06 |
| 0.34807 | NA | NA | NA | NA | 0.460045 | 3 | -374.816 | 755.7026 | 25.83379 | 1.09E-06 |
| -0.1298 | NA | -0.0185 | 0.141037 | NA | 0.461086 | 5 | -373.073 | 756.325 | 26.45614 | 7.96E-07 |
| 0.275092 | NA | 0.020392 | NA | NA | 0.456958 | 4 | -374.783 | 757.6858 | 27.8169 | 4.03E-07 |
| -0.75663 | NA | 0.282165 | 0.158135 | -0.08298 | NA | 5 | -420.067 | 850.3122 | 120.4433 | 3.10E-27 |
| -0.29557 | NA | 0.314667 | NA | -0.07041 | NA | 4 | -421.609 | 851.3371 | 121.4682 | 1.86E-27 |
| -0.28781 | NA | 0.266457 | NA | NA | NA | 3 | -424.554 | 855.1798 | 125.3109 | 2.72E-28 |
| -0.57121 | NA | 0.241112 | 0.097494 | NA | NA | 4 | -423.944 | 856.0074 | 126.1385 | 1.80E-28 |
| 0.035501 | NA | NA | 0.213688 | -0.06844 | NA | 4 | -424.728 | 857.5747 | 127.7058 | 8.22E-29 |
| 0.094885 | NA | NA | 0.155528 | NA | NA | 3 | -427.371 | 860.8127 | 130.9438 | 1.63E-29 |
| 0.813306 | NA | NA | NA | -0.04837 | NA | 3 | -427.573 | 861.2174 | 131.3485 | 1.33E-29 |
| 0.696313 | NA | NA | NA | NA | NA | 2 | -428.989 | 862.0141 | 132.1452 | 8.93E-30 |

## **Table S43 – Model selection for forbs under drought, expansion, s.d.**

| (Intercept) | I((log(size))^2) | log(Nf) | log(Ng) | log(Nl + exp(-1)) | log(size) | df | logLik | AICc | delta | weight |
| --- | --- | --- | --- | --- | --- | --- | --- | --- | --- | --- |
| 0.530842 | NA | NA | NA | -0.02008 | 0.090307 | 4 | -180.254 | 368.627 | 0 | 0.14819 |
| 0.482772 | NA | NA | NA | NA | 0.08966 | 3 | -181.295 | 368.662 | 0.035057 | 0.145615 |
| 0.330261 | NA | 0.058731 | NA | -0.02409 | 0.081544 | 5 | -179.454 | 369.0863 | 0.459301 | 0.117783 |
| **0.335224** | **NA** | **0.041229** | **NA** | **NA** | **0.083418** | **4** | **-180.883** | **369.8858** | **1.258851** | **0.07897** |
| 0.452125 | NA | NA | 0.021667 | -0.02211 | 0.090102 | 5 | -180.13 | 370.4395 | 1.812563 | 0.059872 |
| 0.531765 | 0.005134 | NA | NA | -0.02033 | 0.079689 | 5 | -180.195 | 370.57 | 1.943029 | 0.056091 |
| 0.483016 | 0.003965 | NA | NA | NA | 0.081455 | 4 | -181.261 | 370.6403 | 2.01333 | 0.054154 |
| 0.471596 | NA | NA | 0.002897 | NA | 0.089624 | 4 | -181.293 | 370.7051 | 2.078111 | 0.052428 |
| 0.298931 | NA | 0.056361 | 0.010852 | -0.02494 | 0.081795 | 6 | -179.424 | 371.0989 | 2.471951 | 0.043057 |
| 0.3267 | -0.001 | 0.059721 | NA | -0.02411 | 0.083473 | 6 | -179.452 | 371.1546 | 2.52765 | 0.041874 |
| 0.356018 | NA | 0.043226 | -0.00724 | NA | 0.083206 | 5 | -180.869 | 371.9179 | 3.290913 | 0.02859 |
| 0.333553 | -0.00047 | 0.041688 | NA | NA | 0.084325 | 5 | -180.883 | 371.945 | 3.318023 | 0.028205 |
| 0.457746 | 0.004406 | NA | 0.020338 | -0.0222 | 0.081004 | 6 | -180.088 | 372.4266 | 3.799662 | 0.022168 |
| 0.476641 | 0.003897 | NA | 0.001652 | NA | 0.081575 | 5 | -181.26 | 372.6988 | 4.071854 | 0.019348 |
| 0.556842 | 0.029989 | NA | NA | -0.02123 | NA | 4 | -182.309 | 372.7363 | 4.109326 | 0.018988 |
| 0.506475 | 0.029342 | NA | NA | NA | NA | 3 | -183.456 | 372.9839 | 4.356907 | 0.016778 |
| 0.294393 | -0.00117 | 0.057482 | 0.010989 | -0.02498 | 0.084042 | 7 | -179.421 | 373.1783 | 4.55133 | 0.015223 |
| 0.381829 | 0.025732 | 0.051267 | NA | -0.0245 | NA | 5 | -181.764 | 373.7071 | 5.080165 | 0.011686 |
| 0.354576 | -0.00038 | 0.043583 | -0.00721 | NA | 0.083932 | 6 | -180.869 | 373.9897 | 5.362741 | 0.010146 |
| 0.389369 | 0.026551 | 0.032845 | NA | NA | NA | 4 | -183.223 | 374.5656 | 5.938678 | 0.007608 |
| 0.507442 | 0.029775 | NA | 0.01365 | -0.02249 | NA | 5 | -182.26 | 374.6998 | 6.072893 | 0.007114 |
| 0.526941 | 0.029441 | NA | -0.00533 | NA | NA | 4 | -183.448 | 375.0158 | 6.388881 | 0.006074 |
| 0.366509 | 0.025741 | 0.050165 | 0.005272 | -0.02492 | NA | 6 | -181.757 | 375.7656 | 7.138621 | 0.004175 |
| 0.426471 | 0.026495 | 0.036305 | -0.01288 | NA | NA | 5 | -183.18 | 376.5382 | 7.911241 | 0.002837 |
| 0.229566 | NA | 0.104166 | NA | -0.02632 | NA | 4 | -185.133 | 378.3848 | 9.757869 | 0.001127 |
| 0.232466 | NA | 0.086148 | NA | NA | NA | 3 | -186.786 | 379.6428 | 11.01588 | 0.000601 |
| 0.216783 | NA | 0.103265 | 0.004384 | -0.02666 | NA | 5 | -185.128 | 380.4354 | 11.80848 | 0.000404 |
| 0.550643 | NA | NA | NA | NA | NA | 2 | -188.67 | 381.3747 | 12.74773 | 0.000253 |
| 0.276362 | NA | 0.090074 | -0.0151 | NA | NA | 4 | -186.727 | 381.5728 | 12.94583 | 0.000229 |
| 0.596644 | NA | NA | NA | -0.01902 | NA | 3 | -187.775 | 381.6205 | 12.99351 | 0.000224 |
| 0.506683 | NA | NA | 0.024715 | -0.02134 | NA | 4 | -187.62 | 383.36 | 14.73309 | 9.37E-05 |
| 0.5252 | NA | NA | 0.006579 | NA | NA | 3 | -188.658 | 383.3868 | 14.75983 | 9.24E-05 |

## **Table S44 – Model selection for forbs under drought, colonisation, mean**

| (Intercept) | log(Nf) | log(Ng) | log(Nl + exp(-1)) | df | logLik | AICc | delta | weight |
| --- | --- | --- | --- | --- | --- | --- | --- | --- |
| -0.97488 | 0.358523 | NA | NA | 3 | -111.234 | 228.6906 | 0 | 0.426188 |
| **-1.40088** | **0.341071** | **0.130061** | **NA** | **4** | **-110.509** | **229.3916** | **0.70099** | **0.300181** |
| -0.97784 | 0.356305 | NA | 0.00426 | 4 | -111.228 | 230.8308 | 2.140226 | 0.146169 |
| -1.41922 | 0.335259 | 0.133477 | 0.010283 | 5 | -110.476 | 231.5176 | 2.826994 | 0.103688 |
| -0.29758 | NA | 0.173886 | NA | 3 | -115.121 | 236.4644 | 7.773788 | 0.008741 |
| 0.35875 | NA | NA | NA | 2 | -116.331 | 236.7715 | 8.080933 | 0.007496 |
| -0.42142 | NA | 0.182787 | 0.034292 | 4 | -114.767 | 237.9073 | 9.216729 | 0.004248 |
| 0.28517 | NA | NA | 0.027961 | 3 | -116.098 | 238.4189 | 9.728295 | 0.003289 |

## **Table S45 – Model selection for forbs under drought, colonisation, s.d.**

| (Intercept) | log(Nf) | log(Ng) | log(Nl + exp(-1)) | df | logLik | AICc | delta | weight |
| --- | --- | --- | --- | --- | --- | --- | --- | --- |
| -0.5861 | 0.279935 | NA | NA | 3 | -65.7019 | 137.6259 | 0 | 0.496858 |
| **-0.78721** | **0.271697** | **0.0614** | **NA** | **4** | **-65.3385** | **139.0509** | **1.424998** | **0.243668** |
| -0.58192 | 0.283065 | NA | -0.00601 | 4 | -65.6763 | 139.7264 | 2.100502 | 0.173826 |
| -0.78134 | 0.273556 | 0.060307 | -0.00329 | 5 | -65.331 | 141.228 | 3.602031 | 0.082047 |
| 0.455204 | NA | NA | NA | 2 | -72.5945 | 149.299 | 11.67307 | 0.00145 |
| 0.091682 | NA | 0.09631 | NA | 3 | -71.787 | 149.7963 | 12.17035 | 0.001131 |
| 0.421472 | NA | NA | 0.012819 | 3 | -72.4879 | 151.1981 | 13.57214 | 0.000561 |
| 0.032814 | NA | 0.100541 | 0.016301 | 4 | -71.6137 | 151.6013 | 13.97535 | 0.000459 |

## **Table S46. Selected models for persistence, expansion and colonisation of each treatment group** $\boldsymbol{\times}$ **functional group.**

This table presents all the community models which were used to construct our multi-species IPMs. Cells contain the parameter values used to construct each community model. 0 parameter values are omitted for the sake of readability. Cell colours vary from orange (for negative values) to cyan (for positive values) for overall abundance terms $N_{k}$ to indicate, for a given community vital rate, whether the overall abundance of *k* has a facilitative or competitive effect. The increasing presence of cyan cells further to the right of the table indicates that interactions shift from being primarily competitive to facilitative as water availability decreases (from irrigation to control to drought). Coefficients with p < 0.001 are in bold and italics. Coefficients with p < 0.01 are in bold. Marginally significant coefficients (i.e., with p < 0.1) are in italics. Table 1 is a summary of this table, which presents only the inter-functional group interaction coefficients.

|  | **Treatment** | Irrigation | | | Control | | | Drought | | |
| --- | --- | --- | --- | --- | --- | --- | --- | --- | --- | --- |
| **Community vital rate** | **Func. group**  **Model term** | Grasses | Legumes | Forbs | Grasses | Legumes | Forbs | Grasses | Legumes | Forbs |
| Persistence | Intercept | ***4.008*** | ***6.861*** | ***3.421*** | ***6.253*** | ***4.47*** | ***4.461*** | **3.212** | **5.699** | ***3.873*** |
|  | Log (cover) | ***0.941*** | **1.433** | **0.251** | ***0.51*** | **0.336** | ***0.469*** | ***0.386*** |  | ***0.519*** |
|  | Log (cover)^2^ | *-0.157* | **-0.409** |  |  | -0.081 |  |  |  |  |
|  | log (N_g_) |  |  |  | -0.611 |  | *-0.481* | *-0.56* | ***-1.703*** | -0.147 |
|  | Log (N_l_ + e^-1^) | ***-1.01*** | ***-1.491*** | **-0.274** | ***-0.678*** | ***-0.008*** | -0.038 | *-0.22* |  |  |
|  | Log (N_f_) | -0.299 | -0.475 | ***-0.678*** | **-0.418** |  | ***-0.591*** |  | 0.497 | ***-0.728*** |
| Expansion, mean | Intercept | ***1.943*** | ***1.045*** | ***0.402*** | ***2.345*** | ***0.731*** | ***-0.417*** | ***1.915*** | -0.07 | -0.094 |
|  | Log (cover) | ***0.618*** | ***0.598*** |  | ***0.39*** | ***0.326*** | ***0.165*** | ***0.316*** | 0.204 | ***0.219*** |
|  | Log (cover)^2^ |  |  | ***0.15*** | **0.074** | ***0.113*** | ***0.102*** | **0.088** | *0.138* | ***0.117*** |
|  | Log (N_g_) | *-0.347* |  |  | ***-0.425*** |  | ***0.236*** | -0.13 |  | **0.163** |
|  | Log (N_l_ + e^-1^) |  | -0.193 | -0.038 |  | -0.059 | **-0.057** | -0.19 |  | ***-0.075*** |
|  | Log (N_f_) |  |  |  |  |  |  |  | 0.187 |  |
| Expansion, s.d. | Intercept | ***0.641*** | ***0.575*** | ***0.367*** | **0.141** | **0.598** | -0.203 | ***1.618*** | ***0.5*** | **0.335** |
|  | log(cover) | *0.065* | **0.329** | -0.033 | *0.102* | *0.056* | *0.046* | ***0.109*** | **0.304** | ***0.083*** |
|  | log(cover)^2^ |  | -0.086 | **0.028** | -0.02 |  | ***0.029*** |  | *-0.071* |  |
|  | Log (N_g_) |  |  |  |  |  | ***0.124*** | ***-0.265*** |  |  |
|  | Log (N_l_ + e^-1^) |  |  | **-0.035** |  | *-0.074* | ***-0.041*** |  |  |  |
|  | Log (N_f_) |  |  | 0.048 | ***0.141*** | 0.087 | ***0.086*** |  |  | 0.041 |
| Colonisation, mean | Intercept | ***0.729*** | ***0.428*** | -0.291 | -0.864 | ***0.289*** | ***-1.258*** | ***0.757*** | -0.58 | **-1.401** |
|  | Log (N_g_) |  |  |  | 0.386 |  | **0.25** |  | 0.38 | 0.13 |
|  | Log (N_l_ + e^-1^) |  |  |  |  |  | -0.011 |  | *-0.146* |  |
|  | Log (N_f_) |  |  | **0.179** |  |  | ***0.171*** |  |  | ***0.341*** |
| Colonisation, s.d. | Intercept | ***0.783*** | ***0.45*** | 0.116 | -0.213 | ***0.409*** | ***-1.063*** | ***0.765*** | 0.23 | **-0.787** |
|  | Log (N_g_) |  |  |  | 0.222 |  | ***0.208*** |  |  | 0.061 |
|  | Log (N_l_ + e^-1^) |  |  |  |  |  |  |  | **-0.117** |  |
|  | Log (N_f_) |  |  | ***0.112*** |  |  | ***0.185*** |  | 0.153 | ***0.272*** |

# **Details of model implementation to find overall abundance equilibrium**

We ran our IPMs for 300 time-steps, since the values of *N_g_*, *N_l_*, and *N_f_* for each functional group (and therefore, their corresponding IPMs) did not change significantly when running for further time-steps (changes in overall abundance and the value of the dominant eigenvalue were <10^-6^ units for the last 10 timesteps for each functional group $\times$ treatment). The percentage-cover stage-structure of each functional group *k* at time-step *t* was represented as a 200-entry vector *V_k, t,_* where the *i-*th entry is the number of species of functional group *k* at time *t* with cover between i/2 – 0.5% and *i/2%*. For each treatment, *V_k,0_* was calculated from the distribution of percentage covers of species in each replicate block and taking the element-wise mean. At *t*, to extract the overall abundance values *N_k, t_* from the percentage-cover distribution vectors *V_k, t_* we summed all entries of *V_k, t_*, with each entry weighted by the corresponding midpoint. Then, we used the values of *N_g, t_*, *N_l, t_* and *N_f, t_* to generate *K_t_*, the projection kernel that operated from time *t* to *t+1*. Finally, the new percentage-cover distributions *V_k, t + 1_* were calculated as *V_k, t +1_* = *K_t_* ⋅ *V_k, t_* (Ellner et al. 2016), thus starting the next time-step.

# **Mathematical details of our pseudospectral analysis**

Let *M* be a square matrix, such as an IPM after it has been discretised (Easterling et al. 2010), or a community matrix containing species competition coefficients (Kot 2001). The ϵ-pseudospectrum of *M* is the region consisting of the eigenvalues $M$ has when its entries are subjected to noise of magnitude $\epsilon$, given by

$$\begin{aligned} \Lambda_{\epsilon}=\left\{ z\mathbb{\in C:}\left\| \left( zI-M \right)^{-1} \right\|\geq\epsilon^{-1} \right\}\#\left( 9 \right) \end{aligned}$$

Biologically, the ϵ-pseudospectrum Λ_ϵ_ of *M* measures the extent to which the *response* of a community to some dynamical perturbation (change of cover, in this case) is altered by a structural perturbation of size $\epsilon$ to the entries of *M* (Trefethen & Embree 2005). Crucially, for a discrete-time matrix model *M* representing a stable system with all eigenvalues within the unit circle of the complex plane, its ϵ-pseudospectrum illuminates its transient behaviour after being perturbed out of equilibrium. Let ρ_ϵ_ be the maximum absolute value attained by the points in the $\epsilon$-pseudospectrum Λ_ϵ_ of *M*, and let T_ϵ_ = (ρ_ϵ_ - 1) / ϵ. Then we have the following inequality (Trefethen & Embree 2005):

$$\begin{aligned} sup_{t\geq0}\left| \left| M^{t} \right| \right|\geq sup_{\epsilon\geq0}T_{\epsilon}=sup_{\epsilon\geq0}\frac{\rho_{\epsilon}(M) - 1}{\epsilon}\#\left( 10 \right) \end{aligned}$$

If ρ_ϵ_ < 1 + ϵ, then *T_ϵ_* < 1 for all ϵ, and thus so are all matrix norms $\left| \left| M^{t} \right| \right|$ for *t* ≥ 0. But if ρ_ϵ_ > 1 + ϵ, or equivalently if *T_ϵ_* > 1, then although the system is asymptotically stable, it is transiently unstable — *i.e.*, perturbations are initially amplified by the system.

# **Construction of the overall community matrices for each treatment**

The discretized form of equation 8 in the main text is

$$\begin{aligned} n_{k,y}\left( t+1 \right)=\sum_{x} K_{k,yx}\left( t \right)n_{k,x}\left( t \right),\#\left( S1 \right) \end{aligned}$$

where *n _k, x_* (*t*) is the number of species from functional group *k* with percentage-cover *x* at time *t*, and *K_k, yx_* (*t*) is the (y, x)-element of the discretized IPM kernel for group *k* at time-step *t*. To obtain the community matrix, we compute the Jacobian matrix and evaluate it at equilibrium. For a given treatment, the entries of the Jacobian *J*  are defined as

$$\begin{aligned} J_{\left( k,y \right),\left( j,z \right)}=\frac{\partial n_{k,y}\left( t+1 \right)}{\partial n_{j,z}\left( t \right)}.\#\left( S2 \right) \end{aligned}$$

Substituting *n_k, y_* (*t* + 1) from equation S1into equation S2 gives

$$\begin{aligned} J_{\left( k,y \right),\left( j,z \right)}=\frac{\partial}{\partial n_{j,z}\left( t \right)}\sum_{x} K_{k,yx}\left( t \right) n_{k,x}\left( t \right)=\sum_{x} \frac{\partial K_{k,yx}\left( t \right)}{\partial n_{j,z}\left( t \right)}n_{k,x}\left( t \right)+\sum_{x} K_{k,yx}\left( t \right)\delta_{kj}\delta_{xz},\#\left( S3 \right) \end{aligned}$$

where *ẟ_kj_* is the Kronecker delta (equal to 1 if *k = j*, and equal to 0 if *k ≠ j*). Calculating the second sum in equation S3, we then obtain

$$\begin{aligned} J_{\left( k,y \right),\left( j,z \right)}=\sum_{x} \left[ {\frac{\partial K_{k,yx}\left( t \right)}{\partial n_{j,z}\left( t \right)}n}_{k,x}\left( t \right) \right]+K_{k,yz}\left( t \right)\delta_{kj}\#\left( S4 \right) \end{aligned}$$

Then, at equilibrium, substituting *n_j, z_(t) = n_j, z_* and *K_k, yz_* *(t) = K_k, yz_* into equation S4 leads us to the community matrix *C _(k, y), (j, z)_* for the treatment in question, given by

$$\begin{aligned} C_{\left( k,y \right),\left( j,z \right)}=\sum_{x} \left[ {\frac{\partial K_{k,yx}}{\partial n_{j,z}}n}_{k,x} \right]+K_{k,yz}\delta_{kj},\#\left( S5 \right) \end{aligned}$$

where all quantities are understood to be evaluated at equilibrium. We now seek the analytic form of *∂K_k, yx_/∂_nj, z_*. For any functional group *k*, by definition, at equilibrium

$$\begin{aligned} N_{k}=\log\left( \sum_{x} \left[ e^{x}n_{k,x} \right] + {e^{-1}\delta}_{kl} \right)\#\left( S6 \right) \end{aligned}$$

Here, the Kronecker delta term represents the *e^-^*^1^ augmentation applied to the overall abundance for legumes. Then,

$$\begin{aligned} \frac{\partial N_{k}}{\partial n_{j,z}}=\frac{\partial}{\partial n_{j,z}}\left( \sum_{x} \left[ e^{x}n_{k,x} \right] + {e^{-1}\delta}_{kl} \right)\frac{1}{\sum_{x} \left[ e^{x}n_{k,x} \right] + {e^{-1}\delta}_{kl}}=\frac{1}{e^{N_{k}}}\sum_{x} e^{x}\delta_{kj}\delta_{xz}=\frac{e^{z}}{e^{N_{k}}}\delta_{kj}\#\left( S7 \right) \end{aligned}$$

By using the generalised chain rule and substituting equation S7 into S5, we obtain

$$\begin{aligned} C_{\left( k,y \right),\left( j,z \right)}=\sum_{x} \left[ {\frac{\partial K_{k,yx}}{\partial N_{j}}\frac{\partial N_{j}}{\partial n_{j,z}}n}_{k,x} \right]+K_{k,yz}\delta_{kj}=\sum_{x} \left[ {\frac{\partial K_{k,yx}}{\partial N_{j}}\frac{e^{z}}{e^{N_{j}}}n}_{k,x} \right]+K_{k,yz}\delta_{kj}\#\left( S8 \right) \end{aligned}$$

Then, using the expressions for *s_k_*(*x*), *g_k_*(*x, y*) and *f_k_*(*y*) from equations (1–4) in the main text, we obtain the following partial derivatives

$$\begin{aligned} \frac{\partial s_{k}\left( x \right)}{\partial N_{j}}=\beta_{{k, s, N}_{j}}s_{k}\left( x \right)\left[ 1-s_{k}\left( x \right) \right]\#\left( S9 \right) \end{aligned}$$

$$\frac{\partial g_{k}\left( x,y \right)}{\partial N_{j}} =exp\left[ \frac{-\left( y-\mu_{g, k}\left( x \right) \right)^{2}}{2{\sigma_{g, k}\left( x \right)}^{2}} \right] \frac{\partial}{\partial N_{j}}\frac{1}{\sigma_{g, k}\left( x \right)\sqrt{2\pi}}+ \frac{1}{\sigma_{g, k}\left( x \right)\sqrt{2\pi}} \frac{\partial}{\partial N_{j}}exp\left[ \frac{-\left( y-\mu_{g, k}\left( x \right) \right)^{2}}{2{\sigma_{g, k}\left( x \right)}^{2}} \right]=-\frac{\beta_{k, \sigma_{g} ,N_{j}}}{{\sigma_{g, k}\left( x \right)}^{2}\sqrt{2\pi}}exp\left[ \frac{-\left( y-\mu_{g, k}\left( x \right) \right)^{2}}{2{\sigma_{g, k}\left( x \right)}^{2}} \right]+\frac{1}{\sigma_{g, k}\left( x \right)\sqrt{2\pi}} exp\left[ \frac{-\left( y-\mu_{g, k}\left( x \right) \right)^{2}}{2{\sigma_{g, k}\left( x \right)}^{2}} \right]\frac{\partial}{\partial N_{j}}\left[ \frac{-\left( y-\mu_{g, k}\left( x \right) \right)^{2}}{2{\sigma_{g, k}\left( x \right)}^{2}} \right]=- \frac{\beta_{k, \sigma_{g} ,N_{j}}}{\sigma_{g, k}\left( x \right)}g_{k}\left( x,y \right) + g_{k}\left( x,y \right) \frac{\partial}{\partial N_{j}}\left[ \frac{-\left( y-\mu_{g, k}\left( x \right) \right)^{2}}{2{\sigma_{g, k}\left( x \right)}^{2}} \right]$$

$$\begin{aligned} =g_{k}\left( x,y \right)\left[ - \frac{\beta_{k, \sigma_{g} ,N_{j}}}{\sigma_{g, k}\left( x \right)} +\frac{\beta_{k, \mu_{g} ,N_{j}}\sigma_{g, k}\left( x \right)\left( y-\mu_{g, k}\left( x \right) \right)+\beta_{k, \sigma_{g} ,N_{j}}\left( y-\mu_{g, k}\left( x \right) \right)^{2}}{{\sigma_{g, k}\left( x \right)}^{3}} \right]\#\left( S10 \right) \end{aligned}$$

By equations (5–7), and the same reasoning as S10, it follows that

$$\begin{aligned} \frac{\partial f_{k}\left( y \right)}{\partial N_{j}}=f_{k}\left( y \right)\left[ - \frac{\beta_{k, \sigma_{f} ,N_{j}}}{\sigma_{f, k}} +\frac{\beta_{k, \mu_{f} ,N_{j}}\sigma_{f, k}\left( y-\mu_{f, k} \right)+\beta_{k, \sigma_{f} ,N_{j}}\left( y-\mu_{f, k} \right)^{2}}{{\sigma_{f, k}}^{3}} \right]\#\left( S11 \right) \end{aligned}$$

By the product rule,

$$\begin{aligned} \frac{\partial K_{k,yx}}{\partial N_{j}}=\frac{\partial}{\partial N_{j}}\left[ s_{k}\left( x \right)g_{k}\left( x,y \right)+f_{k}\left( y \right) \right]=\frac{\partial s_{k}\left( x \right)}{\partial N_{j}}g_{k}\left( x,y \right)+s_{k}\left( x \right)\frac{\partial g_{k}\left( x,y \right)}{\partial N_{j}}+\frac{\partial f_{k}\left( y \right)}{\partial N_{j}}\#\left( S12 \right) \end{aligned}$$

Finally, substituting equations S9–S11 into S12, and then substituting that into S8, we obtain the analytic form of *C _(k, y), (j, z)_* for every treatment. The resulting matrix is a 600 × 600 matrix whose 200⋅ (*k* – 1) + *y*-th row and 200⋅ (*j* – 1) + *z*-th column is *C _(k, y), (j, z)_,* where *k* = 1 and *j* = 1 correspond to grasses, *k* = 2 and *j* = 2 to legumes, and *k* = 3 and *j* = 3 to forbs. Hence, for example, for a given treatment *K_1_* is the (equilibrium, and hence time-independent) kernel surface for grasses, and *n_2, z_* is the equilibrium number of legume species of cover *z*. Figure 5j-l in the main text show the eigenvalues and pseudospectra of the resulting community matrices for each treatment.

# **References**

Ellner, S. P., Childs, D. Z., & Rees, M. (2016). *Data-driven modelling of structured populations: A practical guide to the integral projection model*. Springer.

Fenollosa, E., Fernandes, P., Hector, A., King, H., Lawson, C. S., Jackson, J., & Salguero-Gómez, R. (2024). Differential responses of community-level functional traits to mid- and late-season experimental drought in a temperate grassland. *Journal of Ecology*, *112*(10), 2292–2306. <https://doi.org/10.1111/1365-2745.14395>

Jackson, J., Middleton, S. L., Lawson, C. S., Jardine, E., Hawes, N., Maseyk, K., Salguero-Gómez, R., & Hector, A. (2024). Experimental drought reduces the productivity and stability of a calcareous grassland. *Journal of Ecology*, *112*(4), 917–931. <https://doi.org/10.1111/1365-2745.14282>

Kot, M. (2001). *Elements of mathematical ecology*. Cambridge University Press.

Peel, M. C., Finlayson, B. L., & McMahon, T. A. (2007). Updated world map of the Köppen-Geiger climate classification. *Hydrol. Earth Syst. Sci.*, *11*(5), 1633–1644. <https://doi.org/10.5194/hess-11-1633-2007>

Török, P., Janišová, M., Kuzemko, A., Rūsiņa, S., & Dajić Stevanović, Z. (2018). Grasslands, their threats and management in Eastern Europe. In V. R. Squires, J. Dengler, H. Feng, & L. Hua (Eds.), Grasslands of the world: Diversity, management and conservation (pp. 64–88). CRC Press.

Trefethen, L. N. (Lloyd N., & Embree, M. (2005). *Spectra and pseudospectra: The behavior of nonnormal matrices and operators*. Princeton University Press.

**Figure S1. Values of *T_ϵ_* for ϵ = 10^-4^ to 10^-1^ for   each treatment, indicating degree of transient instability.** Values of *T_ϵ_* < 0 are not shown as they are uninformative. For every treatment (a – c), sup*_ϵ_*_≥0_ *T_ϵ_* > 1, since all points like above the dashed line representing *T_ϵ_* = 1. This implies every treatment is transiently unstable — *i.e.*, they all transiently amplify small perturbations. Then, since all treatments are asymptotically stable (Figure 5), as *t* → ∞*,* the perturbation is eventually suppressed.

**Figure S1**


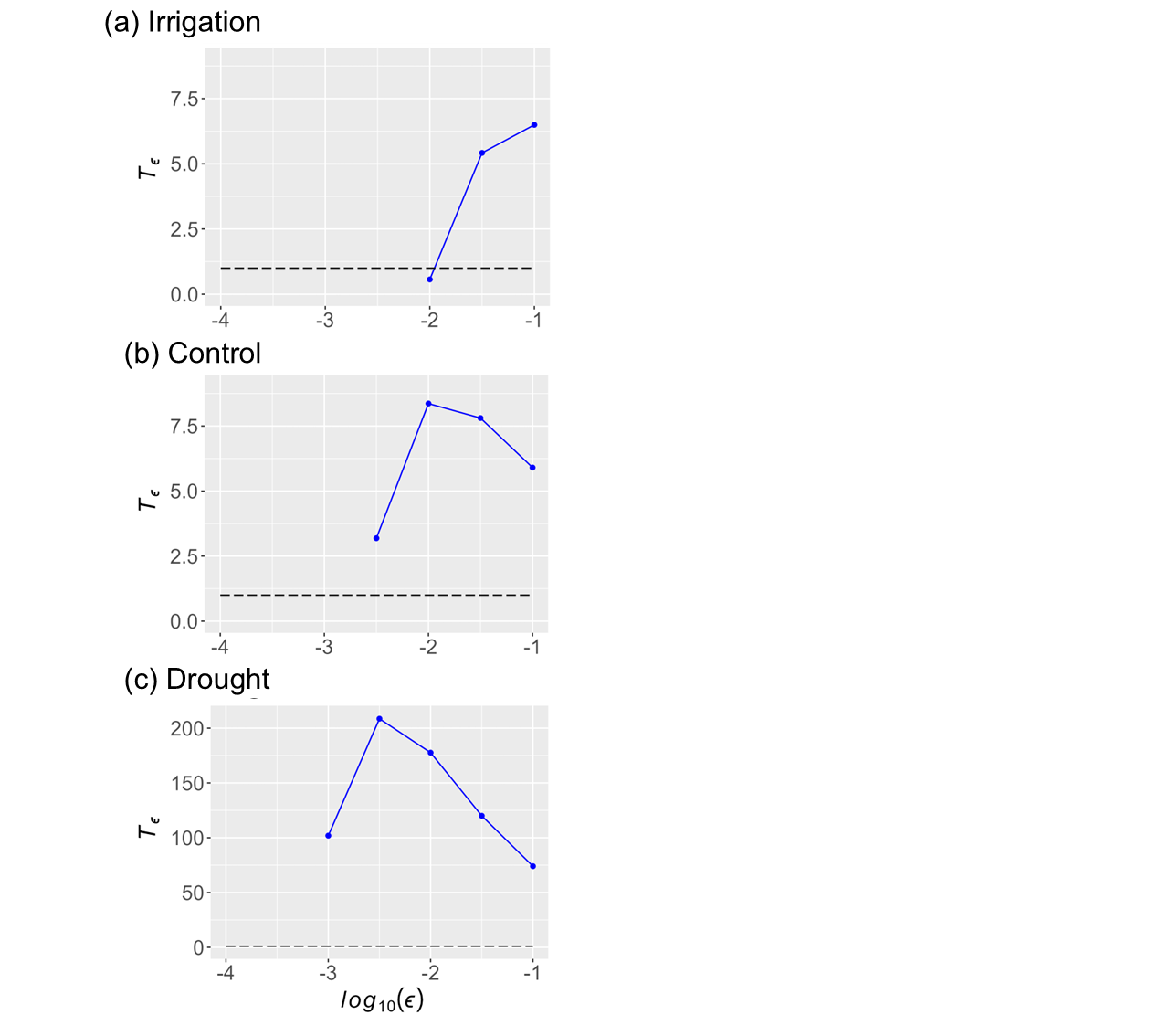

Supplement: Supplementary file 1 — Data S1. [file ELE-28-0-s001.docx]
